# Synthesis of Both **Enantiomers of** Chiral Phenylalanine Derivatives Catalyzed by Cinchona Alkaloid Quaternary Ammonium Salts as Asymmetric Phase Transfer Catalysts

**DOI:** 10.3390/molecules23061421

**Published:** 2018-06-12

**Authors:** Lei Jin, Shuai Zhao, Xin Chen

**Affiliations:** School of Pharmaceutical & Life Sciences, Changzhou University, Changzhou 213164, Jiangsu, China; 15102307@smail.cczu.edu.cn (L.J.); zhaoshuai@cczu.edu.cn (S.Z.)

**Keywords:** unnatural phenylalanine derivatives, phase transfer catalysts, asymmetric α-alkylation, glycine Schiff base

## Abstract

A practical synthesis of both enantiomers of unnatural phenylalanine derivatives by using two pseudoenantiomeric phase transfer catalysts is described. Through asymmetric α-alkylation of glycine Schiff base with substituted benzyl bromides and 1-(bromomethyl)naphthalene under the catalysis of *O*-allyl-*N*-(9-anthracenmethyl) cinchoninium bromide (**1f**) and *O*-allyl-*N*-(9-anthracenylmethyl)cinchonidium bromide (**1i**), respectively, a series of both (*R*)- and (*S*)-enantiomers of unnatural α-amino acid derivatives were obtained in excellent yields and enantioselectivity. The synthetic method is simple and scalable, and the stereochemistry of the products is fully predictable and controlled: the cinchonine-type phase transfer catalyst **1f** resulted in (*R*)-α-amino acid derivatives, and the cinchonidine-type phase transfer catalyst **1i** afforded (*S*)-α-amino acid derivatives.

## 1. Introduction

**Figure 1:**
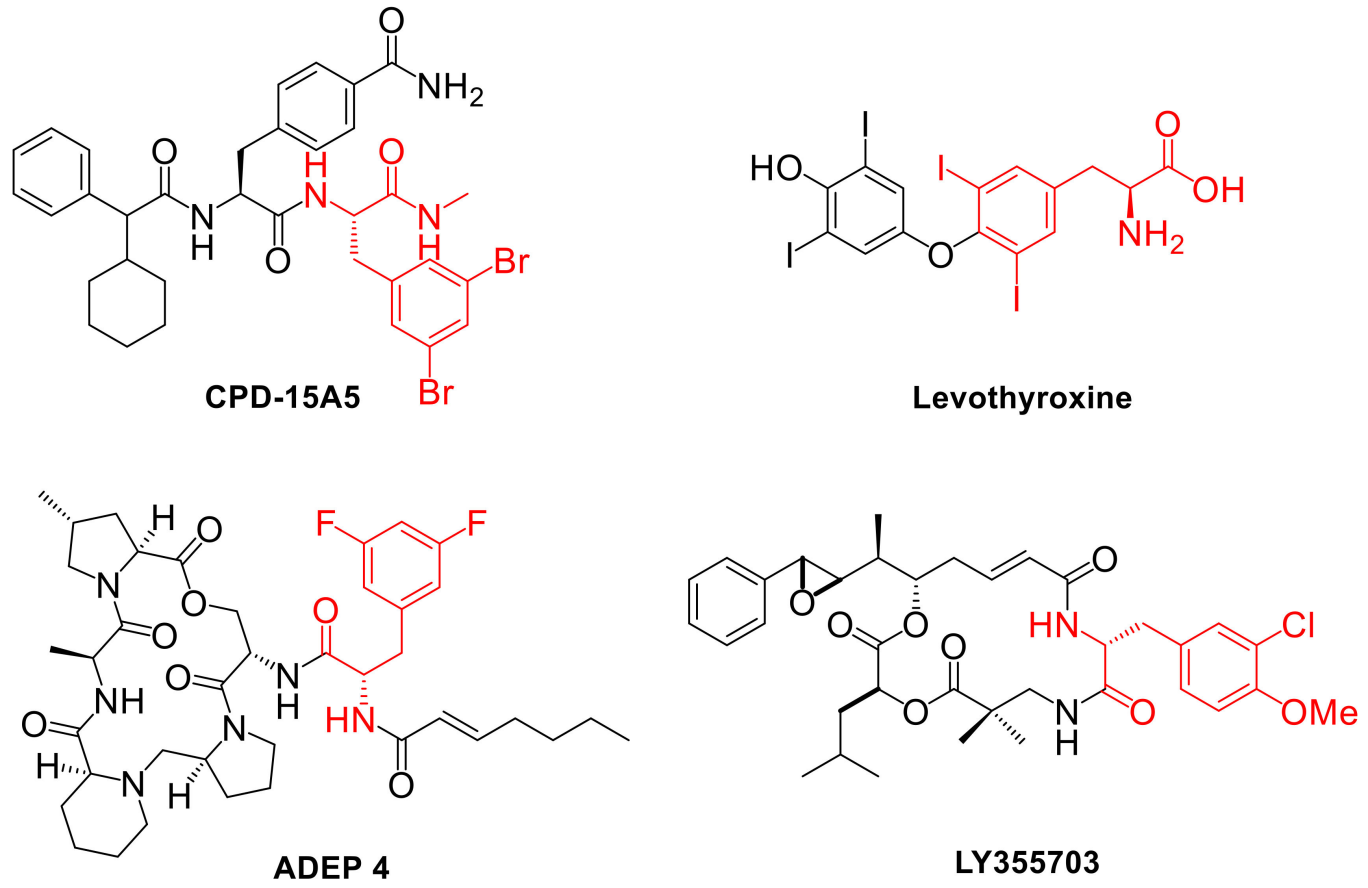
Chemical structures of some biologically active molecules containing α-phenylalanine subunits.

Unnatural α-amino acids are important building blocks for synthesis of peptides, pharmaceutical molecules and natural products. In particular, unnatural α-phenylalanine derivatives have been the subject of numerous investigations for their extensive distribution in biological active compounds. For example, CPD-15A5, which is a small-molecule negative allosteric modulator (antagonist) for the β_2_-adrenergic receptor (β_2_AR) [1], contains a (*S*)-3,5-dibromophenylalanine subunit (Figure 1). Levothyroxine, used for the treatment of hypothyroidism [2,3,4], has a (*S*)-3,5-diiodophenylalanine backbone. ADEP 4, which shows potent antibacterial activity against multidrug-resistant pathogens [5,6], has a (*S*)-3,5-difluorophenylalanine sidechain. In addition, LY355703, a potent and broad spectrum antitumor agent [7,8], is partially composed of (*R*)-(3-chloro-5-methoxy)phenylalanine. We are particularly interested in unnatural α-phenylalanine derivatives [9] because they are the key building blocks for synthesizing a series of dipeptides as allosteric antagonists of the β_2_-adrenergic receptor (β_2_AR) [1] in one of our ongoing research projects. We need both the (*R*)- and (*S*)-enantiomers of α-phenylalanine derivatives for structure-activity relationship studies.

Although many different methods for the synthesis of α-phenylalanine derivatives have been reported in the literature [10,11,12], these methods have significant drawbacks, such as the use of very costly catalysts, low yields, and/or poor enantioselectivity for some derivatives. Asymmetric phase-transfer catalysis has been widely used for the synthesis of chiral α-amino acids because of its operational simplicity, mild reaction conditions, and reduced environmental impact [13,14,15,16,17]. Quaternary cinchona alkaloid catalysts, discovered by O’Donnell et al*.* [18] and further improved by Lygo [19] and Corey [20], have been the most useful and practical chiral phase-transfer catalysts for the synthesis of α-amino acids. On the other hand, only a few examples have been reported for asymmetric synthesis of disubstituted α-phenylalanine derivatives by using quaternary cinchona alkaloid catalysts. Furthermore, some of the reported procedures are not suitable for wide range of substrates. For example, McAlister et al. prepared a series of substituted 2-nitrophenylalanine derivatives through asymmetric alkylation of *N*-(dibenzylidene)glycine *tert*-butyl ester with substituted 2-nitrobenzyl bromides using a cinchonidine phase transfter catalyst. 5-Methyl-2-nitrobenzyl bromide gave 5-methyl-2-nitrophenylalanine derivative with 100% ee; however, when the methyl group was replaced with a trifluoromethyl group, the ee value decreased to 90%, and with a chloro group replacement, the corresponding product has only 75% ee [21].

Our group recently synthesized some biologically important compounds via asymmetric phase transfer catalysis [1,22]. We predicted that both enantiomers of the disubstituted α-phenylalanine derivatives could be obtained by using two pseudoenantiomeric quaternary cinchona alkaloids as the phase transfer catalysts. Our objective was to develop a straightforward preparative-scale method for synthesizing the unnatural α-phenylalanine derivatives with high chemical and optical purities in sufficient quantities to permit rapid preparation of the dipeptides for laboratory bioassays and animal studies. Herein we report a convenient synthesis of both the (*R*)- and (*S*)-enantiomers of α-phenylalanine derivatives, including several disubstituted unnatural α-phenylalanine derivatives which have not been reported in literature, with excellent yield and excellent enantioselectivity through asymmetric phase-transfer catalysis. The described procedure is simple, mild and scalable, and its usefulness has been demonstrated with the synthesis of a dipeptide derivative of the β_2_AR allosteric antagonist CPD-15A5 by using an enantiomer-enriched 3-chlorophenylalanine derivative.

## 2. Results and Discussion

### 2.1. Condition Screening

**Figure 2:**
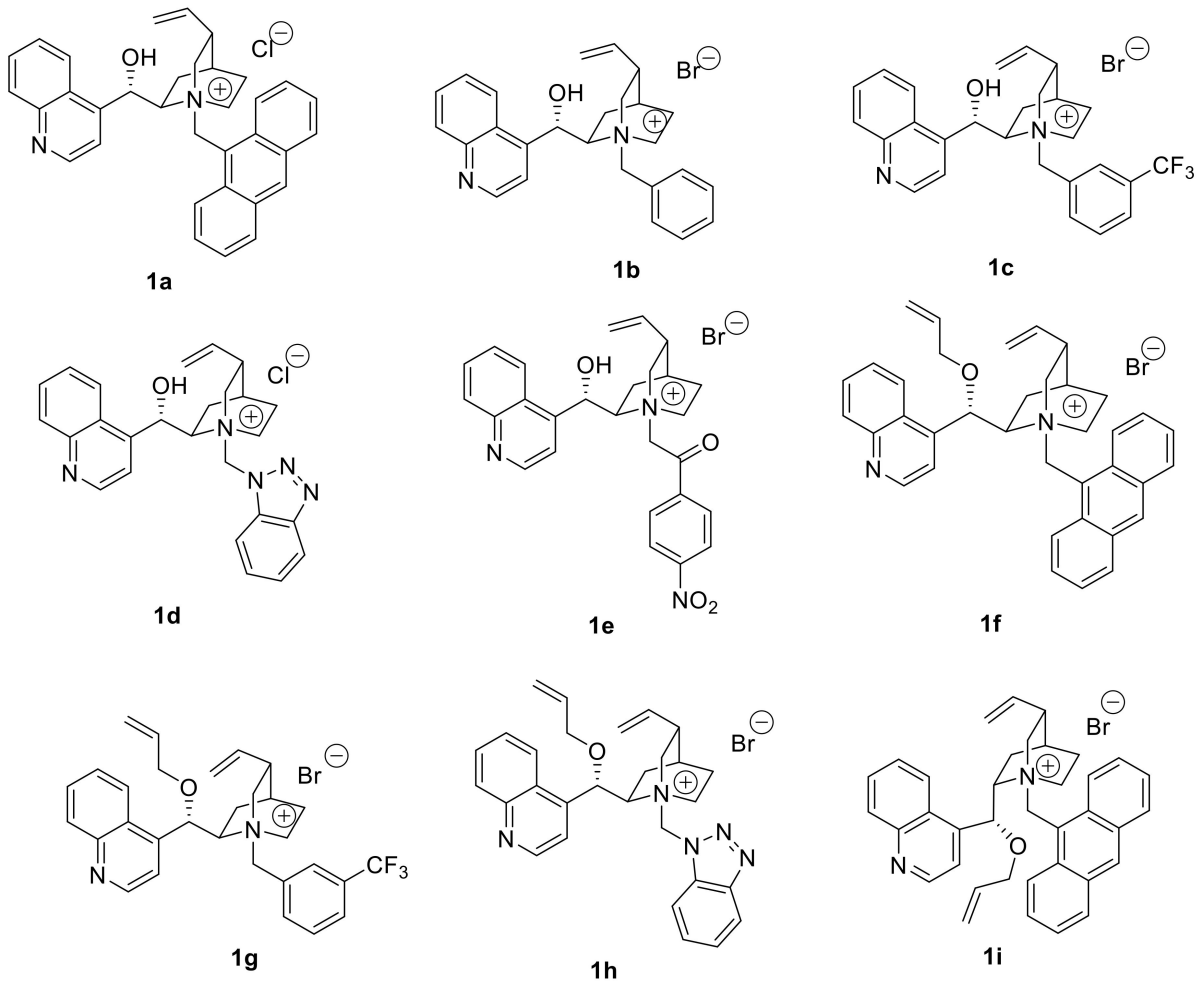
Catalysts **1a**–**1i**.

Chiral unnatural α-phenylalanine derivatives were synthesized through the asymmetric α-alkylation reaction of *N*-(dibenzylidene)glycine *tert*-butyl ester (**2**) [23,24] with substituted benzyl bromides catalyzed by a phase transfer catalyst. Compounds **1a**–**1h** (Figure 2) were chosen as phase transfer catalysts, and they were obtained from cinchonine according to the reported procedures [25,26,27,28,29,30]. 

Initially, we selected 3,5-dichlorophenyl bromide (**3a**) to react with **2** to optimize the asymmetric alkylation conditions similar to our previously described method [22]. Five equivalents of **3a** were reacted with **2** in the presence of 0.1 equivalents of the catalyst and by using 50% aqueous KOH solution as base and toluene/CHCl_3_ as solvent. A similar procedure was reported by Nájera et al*.* for preparing (*S*)-*tert*-butyl *N*-(diphenylmethylene)phenylalaninate [31]. The alkylation proceeded at room temperature for 24 h and afforded (*R*)-*tert*-butyl *N*-(diphenylmethylene)-(3,5-dichloro-phenyl)alaninate (**4a**). The results are summarized in Table 1. Catalyst **1h** gave the best yield (99%) and with moderate enantioselectivity (81% ee; Table 1, entry 8) for the desired product, but catalyst **1f** gave the highest enantioselectivity (94%) (Table 1, entries 1 to 8). Clearly, catalyst **1f** was better than the other screened catalysts for the asymmetric α-alkylation of **2** with **3a**. For achieving better enantioselectivity, the reaction temperature was lowered to −20 °C, and the yield was slightly improved (from 94% to 97%) while the enantioselectivity also increased slightly (from 94% to 96%) when the reaction time was increased to 48 h. When the reaction temperature was lowered to −40 °C, the ee values improved a little bit (from 96% to 97%), but the yield decreased (from 97% to 95%) even after 72 h (Table 1, entry 10). After the reaction temperature was further lowered to −60 °C, both the ee value and yield decreased (Table 1, entry 9). In addition, increasing or decreasing the catalyst’s amount didn’t improve the enantioselectivity (no better than 96% ee). However, less amount of catalyst (5 mol%) resulted in lower yield (89%, Table 1, entry 12) whereas 20 mol% of catalyst only increased the yield a little bit (from 95% to 99%. Table 1, entry 13) with slightly shorter reaction time (60 h). Considering the reaction efficiency and enantioselectivity, the optimized conditions for asymmetric α-alkylation of **2** with **3a** were determined to be: **1f** (10 mol%), toluene/CHCl_3_, and 50% KOH (5 equivalents) at −40 °C.

### 2.2. Substrate Expansion

With the optimal reaction conditions in hand, we investigated the scope and limitations of the asymmetric α-alkylation of glycine Schiff base **2**. A variety of disubstituted and monosubstituted benzyl bromides **3a-o** as well as 1-(bromomethyl)naphthalene (**3o**) were tested for the alkylation reaction with **2**, and the results are outlined in Table 2. A variety of substituents, such as halo (F, Cl, Br and I), electron-withdrawing (nitro and difluoro groups), electron-donating (dimethoxy group) and α-naphthyl groups, were well tolerated under the alkylation conditions, affording the desired products **4a**–**4n**. Eight disubstituted unnatural α-phenylalanine derivatives **4a**–**4h** (Table 2, entries 1–8) were obtained with satisfactory yields and enantioselectivity. When the benzyl bromides containing strong electron-withdrawing groups were used, the corresponding α-phenylalanine derivatives were prepared with excellent enantioselectivity. Under the same alkylation conditions, 1-(bromomethyl)naphthalene was reacted smoothly with **2**, affording (*R*)-*tert*-butyl 2-((diphenylmethylene)amino)-3-(1-naphthyl)propanoate (**4o**) with 85% yield and 97% ee (Table 2, entry 15).

After derivatives **4a-o** were successfully obtained under the catalysis of cinchonine-type phase transfer catalyst **1f**, we tried to synthesize the enantiomers of **4a-o** by using a cinchonidine-type phase transfer catalyst, *O*-allyl-*N*-(9-anthracenemethyl) cinchonidium bromide (**1i**, Table 3), which is the pseudoenantiomer of **1f** and was prepared according to the same procedure used for **1f**. To our satisfaction, all the enantiomers of **4a**–**4o** were obtained with good to excellent yields (71% to 99%) and excellent enantioselectivity (93% to 99% ee) (Table 3) under the identical conditions for **4a**–**4o** except that catalyst **1f** was replaced with **1i**. Similar to the results in Table 2, alkylation of **2** with 2-chloro-6-fluorobenzyl bromide resulted in the highest enantioselectivity under the catalyst of **1h** (99% ee; Table 3, entry 5).

Finally, the absolute configuration of the newly synthesized α-amino acid derivatives **4a**–**4o** and **4a’**–**4o’** was established by comparison of their optical rotation values with those reported in the literature. For example, the (*S*)-configuration of **4l’** was confirmed by comparing its optical rotation value ([α${]}_{D}^{20}$ −105.4°, *c* = 1.09, CHCl_3_) with the reported result ([α${]}_{D}^{20}$ −110.1°, *c* = 1.09, CHCl_3_) [32]. Gratzer et al. synthesized the same compound through asymmetric α-alkylation of glycine Schiff base catalyzed by *p*-biphenyl-containing pyrrolidinium ammonium bromide in 79% yield and 80% ee [32]. The (*R*)-configuration of **4o** was established by comparison of its optical rotation value ([α${]}_{D}^{20}$ 331.6°, *c* = 1, CH_2_Cl_2_) with the reported value ([α${]}_{D}^{20}$ 343.7°, *c* = 1.19, CHCl_3_) [33]. After synthesizing **4o** via asymmetric α-alkylation of glycine Schiff base catalyzed by Maruoka catalyst [15], Ooi et al. cleaved the benzophenone imine and *tert*-butyl ester with 6 N HCl, protected the amino group with Boc, and then confirmed the (*R*)-configuration by comparing the HPLC retention time of the *N*-Boc protected amino acid with the literature value [33]. Therefore, (*R*)-configuration was assigned for **4a**–**4o**, and (*S*)-configuration for **4a’**–**4o’**.

### 2.3. Application

**Scheme 1:**
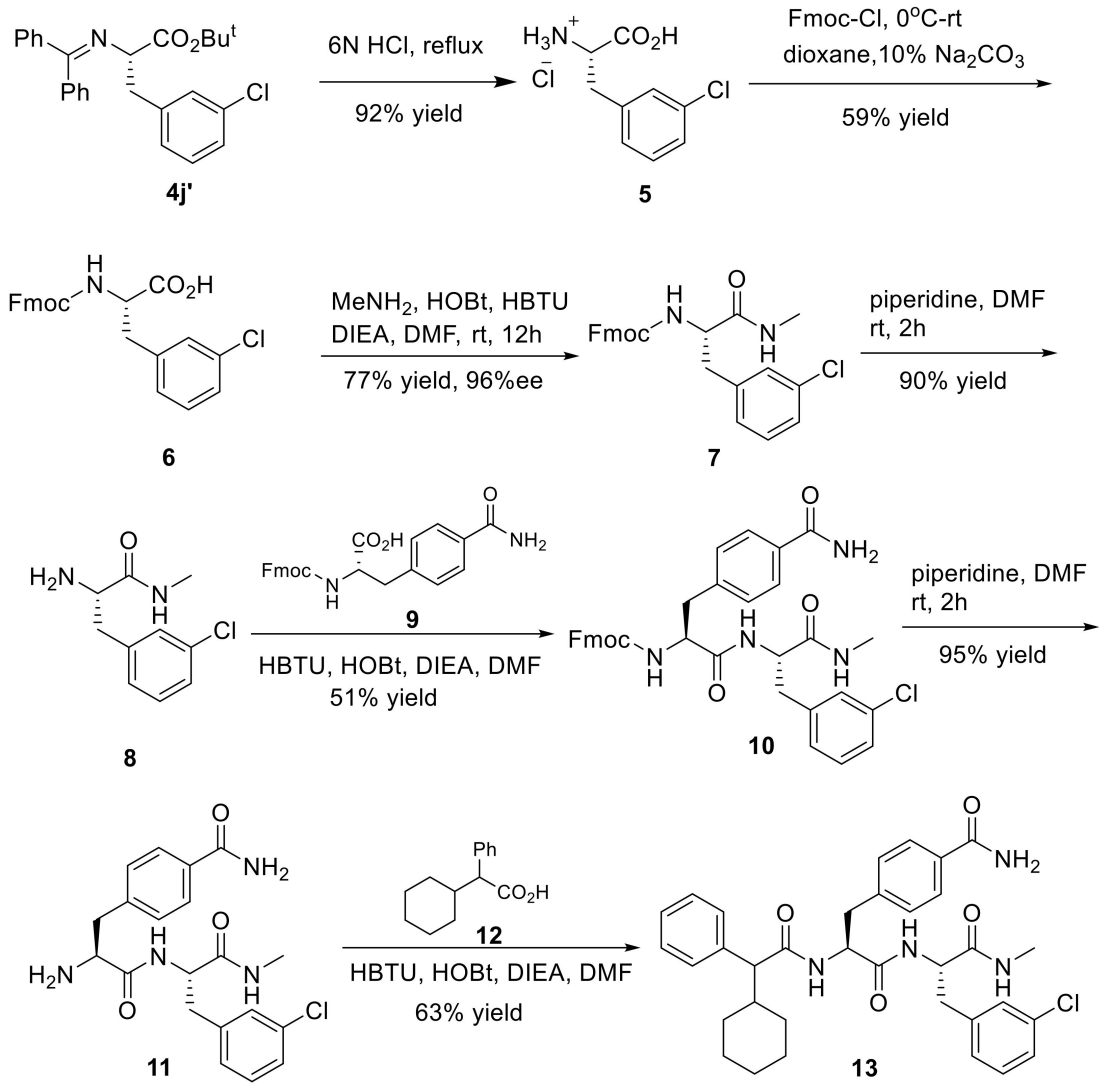
Synthetic route to **13**.

The asymmetric α-alkylation of glycine Schiff base with substituted benzyl bromides can be applied to the synthesis of new derivatives of CPD-15A5 as allosteric antagonists for the β_2_AR, such as (2*S*)-3-(3-chlorophenyl)-2-((2*S*)-2-(2-cyclohexyl-2-phenylacetamido)-3-phenylpropanamido)-*N*-methylpropanamide (**13**, Scheme 1). (*S*)-*tert*-Butyl *N*-(diphenylmethylene)-(3-chlorophenyl)alaninate (**4j’**) was hydrolyzed in refluxing hydrochloric acid to give (*S*)-3-chlorophenylalanine hydrochloride (**5**) in 92% yield, and then the amino group in **5** was protected with Fmoc-Cl, affording Fmoc-protected (*S*)-(3-chloro)phenylalanine (**6**). In the next step, the Fmoc-protected l-phenylalanine methylamide **7** was obtained by condensation of **6** with methylamine in the presence of *O*-benzo-triazol-1-yl-*N,N,N′,N′*-tetramethyluronium hexafluorophosphate (HBTU) and hydroxybenzotriazole (HOBt). It should be pointed out that the acidic hydrolysis of **4j’** didn’t racemize the amino acid, because the ee value of **7** is 96%. After Fmoc deprotection of **7** (piperidine/DMF), the resulting l-phenylalanine methylamide **8** was coupled in the presence of HBTU/HOBt with Fmoc-l-4-carbamoylphenylanine (**9**) to generate dipeptide **10** in 51% yield. Upon treatment with piperidine in DMF, the Fmoc group in **10** was removed smoothly at room temperature, giving the corresponding amine **11** in 95% yield. In the final step, **11** was reacted with 2-cyclohexyl-2-phenyl acetic acid (**12**) to afford the desired product **13** in 63% yield.

## 3. Materials and Methods

### 3.1. Instruments and Reagents

Melting points were measured on SGW X-4B melting point apparatus (Shenguang, Shanghai, China). ^1^H-NMR spectra were recorded on Avance 300 (300 MHz) and 400 (400 MHz) spectrometers (Bruker, Karlsruhe, Germany). Chemical shifts were reported in ppm from tetramethylsilane with the solvent resonance resulting from incomplete deuterium incorporation as the internal standard (CDCl_3_: δ 7.26 ppm). ^13^C-NMR spectra were recorded on Bruker Avance 300 (75 MHz) and 400 (100 MHz) spectrometers with complete proton decoupling. Chemical shifts were reported in ppm from tetramethylsilane with the solvent resonance as the internal standard. High-resolution mass spectrometry was performed on a Thermo Orbitrap Elite, instrument (Agilent, Palo Alto, CA, USA). Optical rotations were measured on an Autopol IV (d = 589 nm, Hg lamp, 50 mm cell) instrument (Rudolph, NJ, USA). The enantiomeric excess was determined by a 1260 infinity series HPLC (Agilent, Palo Alto, CA, USA) equipped with Chiralpak OD-H, AD-H and IA columns (4.6 mm × 250 mm, Daicel Chiral Technologies, Shanghai, China). Chemicals and solvents were purchased from Linfeng (Shanghai) and Annaiji (Shanghai) in China, and used as received. Purification of the products was carried out by flash column chromatography using silica gel (Yantai Jiangyou Company, Shandong, China, particle size 0.100–0.075 mm).

### 3.2. General Methods

*(R)-tert-Butyl N-(diphenylmethylene)-(3,5-dichlorophenyl)alaninate* (**4a**; R=3,5-Cl_2_-C_6_H_3_): A 10 mL reaction tube was charged with **2** (30 mg, 0.1 mmol), 3,5-dichlorobenzyl bromide (119 mg, 0.5 mmol, 5 equivalent), catalyst **1f** (6 mg, 0.01 mmol, 0.1 equivalent) and toluene and CHCl_3_ (1.5 mL, 2:1 *v*/*v*), and the mixture was cooled to −40 °C. After the mixture was stirred for 10 min, 50% aq. KOH (28 μL, 0.1 mmol, 5 equivalent) was added, and the whole reaction mixture was stirred at −40 °C for 72 h before being allowed to warm to ambient temperature. The reaction was quenched by adding H_2_O (2 mL), and the resulting mixture was extracted with EtOAc (3 × 10 mL). The combined extracts were washed with brine (10 mL) and dried (anhydrous Na_2_SO_4_), and the crude product was purified by flash column chromatography (eluting with hexane/EtOAc, 50:1) to afford **4a** (43 mg, 95% yield) as light yellow liquid. 97% ee; [α${]}_{D}^{20}$ 178.8° (*c* = 1.0, CH_2_Cl_2_); ^1^H-NMR (400 MHz, CDCl_3_): δ 7.57 (s, 1H), 7.55 (d, *J* = 1.4 Hz, 1H), 7.40–7.29 (m, 6H), 7.16 (t, *J* = 1.7 Hz, 1H), 6.95 (d, *J* = 1.7 Hz, 2H), 6.76 (d, *J* = 6.1 Hz, 2H), 4.12 (dd, *J* = 8.9, 4.6 Hz, 1H), 3.19–3.08 (m, 2H), 1.45 (s, 9H); ^13^C-NMR (100 MHz, CDCl_3_): δ 171.1, 170.2, 141.7, 139.2, 136.1, 134.4, 130.3, 128.7, 128.6, 128.3, 128.3, 128.0, 127.6, 126.4, 81.6, 66.9, 38.9, 28.0; HRMS (ESI, positive): Calcd. for C_26_H_26_Cl_2_NO_2_ [M + H]^+^ 454.1335, found: 454.1333. HPLC analysis: Daicel Chiralcel OD-H, *n*-hexane/isopropanol = 95:5, flow rate = 0.5 mL/min.

*(S)-tert-Butyl N-(*diphenylmethylene*)-(3,5-dichlorophenyl)alaninate* (**4a’**; R=3,5-Cl_2_-C_6_H_3_): Under the same reaction conditions for **4a** except that catalyst **1f** was replaced with **1i**, enantiomer **4a’** was obtained as light yellow liquid. 98% yield; 97% ee; [α${]}_{D}^{29}$ −183.0° (*c* = 1.0, CH_2_Cl_2_).

*(R)-tert-Butyl N-(diphenylmethylene)-(3,5-difluorophenyl)alaninate* (**4b**; R=3,5-F_2_-C_6_H_3_): Under the same reaction conditions for **4a** except that 3,5-dichlorophenyl bromide was replaced with 3,5-difluorobenzyl bromide, **4b** was obtained as white solid. M.p. 35–37 °C; 98% yield; 94% ee; [α${]}_{D}^{29}$ 150.2° (*c* = 1.0, CH_2_Cl_2_); ^1^H-NMR (400 MHz, CDCl_3_): δ 7.58 (s, 1H), 7.56 (d, *J* = 1.3 Hz, 1H), 7.39–7.25 (m, 6H), 6.78 (d, *J* = 6.4 Hz, 2H), 6.63–6.59 (m, 3H), 4.13 (dd, *J* = 8.9, 4.4 Hz, 1H), 3.23–3.10 (m, 2H), 1.44 (s, 9H); ^13^C-NMR (100 MHz, CDCl_3_): δ 170.9, 170.2, 163.9, 163.8, 161.5, 161.3, 142.4, 142.3, 142.2, 139.2, 136.1, 130.3, 128.7, 128.5, 128.2, 128.0, 127.6, 112.6, 112.6, 112.4, 112.3, 101.9, 101.6, 101.4, 81.5, 67.1, 39.3, 28.0; HRMS (ESI, positive): calcd. for C_26_H_26_F_2_NO_2_ [M + H]^+^ 422.1926, found: 422.1925. HPLC analysis: Daicel Chiralcel OD-H, *n*-hexane/isopropanol = 98:2, flow rate = 0.5 mL/min.

*(S)-tert-Butyl N-(diphenylmethylene)-(3,5-difluorophenyl)alaninate* (**4b’**; R=3,5-F_2_-C_6_H_3_): Under the same reaction conditions for **4b** except that catalyst **1f** was replaced with **1i**, enantiomer **4b’** was obtained as white solid. 99% yield; 94% ee; [α${]}_{D}^{29}$ −154.6° (*c* = 1.0, CH_2_Cl_2_).

*(R)-tert-Butyl N-(diphenylmethylene)-(3,5-dibromophenyl)alaninate* (**4c**; R=3,5-Br_2_-C_6_H_3_): Under the same reaction conditions for **4a** except that 3,5-dichlorophenyl bromide was replaced with 3,5-dibromobenzyl bromide, **4c** was obtained as colorless liquid. 95% yield; 93% ee; [α${]}_{D}^{29}$ 174.6° (*c* = 1.0, CH_2_Cl_2_); ^1^H-NMR (400 MHz, CDCl_3_): δ 7.56 (s, 1H), 7.54 (d, *J* = 1.2 Hz, 1H), 7.46 (s, 1H), 7.39–7.25 (m, 6H), 7.15 (d, *J* = 1.4 Hz, 2H), 6.76 (d, *J* = 5.3 Hz, 2H), 4.10 (dd, *J* = 8.8, 4.6 Hz, 1H), 3.18–3.07 (m, 1H), 1.46 (s, 1H); ^13^C-NMR (100 MHz, CDCl_3_): δ 171.1, 170.1, 142.3, 139.2, 136.1, 131.8, 131.6, 130.3, 128.7, 128.6, 128.3, 128.0, 127.6, 122.4, 81.6, 66.9, 38.8, 28.0; HRMS (ESI, positive): Calcd. for C_26_H_26_Br_2_NO_2_ [M + H]^+^ 542.0325, 544.0304, found: 542.0326, 544.0306. HPLC analysis: Daicel Chiralcel OD-H, *n*-hexane/isopropanol = 95:5, flow rate = 0.5 mL/min.

*(S)-tert-Butyl N-(diphenylmethylene)-(3,5-dibromophenyl)*alaninate (**4c’**; R=3,5-Br_2_-C_6_H_3_): Under the same reaction conditions for **4c** except that catalyst **1f** was replaced with **1i**, enantiomer **4c’** was obtained as colorless liquid. 83% yield, 95% ee; [α${]}_{D}^{29}$ −86° (*c* = 1.3, CH_2_Cl_2_).

*(R)-tert-Butyl N-(diphenylmethylene)-(3-chloro-5-fluorophenyl)alaninate* (**4d**; R=3-Cl-5-F-C_6_H_3_): Under the same reaction conditions for **4a** except that 3,5-dichlorophenyl bromide was replaced with 3**-**chloro-5-fluorophenyl bromide, **4d** was obtained as colorless liquid. 93% yield; 97% ee; [α${]}_{D}^{20}$ 169.0° (*c* = 1.0, CH_2_Cl_2_); ^1^H-NMR (400 MHz, CDCl_3_): δ 7.57 (d, *J* = 7.2 Hz, 2H), 7.41–7.28 (m, 6H), 6.89 (d, *J* = 8.4 Hz, 1H), 6.85 (s, 1H), 6.78 (d, *J* = 5.6 Hz, 2H), 6.71 (d, *J* = 9.2 Hz, 1H), 4.13 (q, *J* = 4.4 Hz, 1H), 3.31–3.10 (m, 2H), 1.45 (s, 9H); ^13^C-NMR (100 MHz, CDCl_3_): δ 171.1, 170.3, 163.7, 161.2, 142.3, 142.2, 139.3, 136.3, 134.5, 134.4, 130.5, 128.8, 128.7, 128.4, 128.1, 127.7, 125.9, 125.9, 115.4, 115.2, 114.2, 114.0, 81.7, 67.1, 39.2, 28.1; HRMS (ESI, positive): Calcd. for C_26_H_25_ClFNaNO_2_ [M + Na]^+^ 460.1450, found: 460.1450. HPLC analysis: Daicel Chiralcel OD-H, *n*-hexane/isopropanol = 95:5, flow rate = 0.5 mL/min.

*(S)-tert-Butyl N-(diphenylmethylene)-(3-chloro-5-fluorophenyl)alaninate* (**4d’**; R=3-Cl-5-F-C_6_H_3_): Under the same reaction conditions for **4d** except that catalyst **1f** was replaced with **1i**, enantiomer **4d’** was obtained as colorless liquid. 97% yield, 98% ee; [α${]}_{D}^{20}$ −168.2° (*c* = 1.0, CH_2_Cl_2_).

*(R)-tert-Butyl N-(diphenylmethylene)-(2-chloro-6-fluorophenyl)alaninate* (**4e**; R=2-Cl-6-F-C_6_H_3_): Under the same reaction conditions for **4a** except that 3,5-dichlorophenyl bromide was replaced with 2**-**chloro-6-fluorophenyl bromide, **4e** was obtained as colorless liquid. 68% yield; 98% ee; [α${]}_{D}^{20}$ 274.2° (*c* = 0.9, CH_2_Cl_2_); ^1^H-NMR (300 MHz, CDCl_3_): δ 7.60–7.57 (m, 2H), 7.36–7.26 (m, 6H), 7.11–7.01 (m, 2H), 6.88–6.81 (m, 1H), 6.68 (d, *J* = 6.9 Hz, 2H), 4.39–4.34 (m, 1H), 3.52–3.45 (m, 1H), 3.36–3.29 (m, 1H), 1.45 (s, 9H); ^13^C-NMR (75 MHz, CDCl_3_): δ 170.8, 170.6, 163.6, 160.3, 139.6, 136.2, 136.1, 136.0, 130.2, 129.0, 128.5, 128.2, 128.1, 128.0, 127.8, 125.1, 125.0, 124.7, 124.5, 114.0, 113.7, 81.4, 64.6, 30.1, 28.1; HRMS (ESI, positive): Calcd. for C_26_H_25_ClFNO_2_Na [M + Na]^+^ 460.1450, found: 460.1447. HPLC analysis: Daicel Chiralcel IC, *n*-hexane/isopropanol = 98:2, flow rate = 0.5 mL/min.

*(S)-tert-Butyl N-(diphenylmethylene)-(2-chloro-6-fluorophenyl)alaninate* (**4e’**; R=2-Cl-6-F-C_6_H_3_): Under the same reaction conditions for **4e** except that catalyst **1f** was replaced with **1i**, enantiomer **4e’** was obtained as colorless liquid. 74% yield, 99% ee; [α${]}_{D}^{20}$ −231.0° (*c* = 0.8, CH_2_Cl_2_).

*(R)-tert-Butyl N-(diphenylmethylene)-(3-chloro-4-fluorophenyl)alaninate* (**4f**; R=3-Cl-4-F-C_6_H_3_): Under the same reaction conditions for **4a** except that 3,5-dichlorophenyl bromide was replaced with 3**-**chloro-4-fluorophenyl bromide, **4f** was obtained as colorless liquid. 86% yield; 97% ee; [α${]}_{D}^{20}$ 178.5° (*c* = 1.1, CH_2_Cl_2_); ^1^H-NMR (300 MHz, CDCl_3_): δ 7.59–7.55 (m, 2H), 7.41–7.27 (m, 6H), 7.07 (d, *J* = 7.2 Hz, 1H), 6.97 (s, 1H), 6.94 (d, *J* = 1.2 Hz, 1H), 6.73 (d, *J* = 6.0 Hz, 1H), 4.10 (q, *J* = 4.9 Hz, 1H), 3.20–3.07 (m, 2H), 1.45 (s, 9H); ^13^C-NMR (75 MHz, CDCl_3_): δ 170.9, 170.5, 158.5, 155.2, 139.4, 136.3, 135.5, 131.8, 130.5, 129.7, 128.8, 128.6, 128.4, 128.1, 127.7, 120.5, 120.3, 116.2, 116.0, 81.6, 67.5, 38.6, 28.2; HRMS (ESI, positive): Calcd. for C_26_H_25_ClFNO_2_Na [M + Na]^+^ 460.1450, found: 460.1445. HPLC analysis: Daicel Chiralcel OD-H, *n*-hexane/isopropanol = 95:5, flow rate = 0.5 mL/min.

*(S)-tert-Butyl N-(diphenylmethylene)-(3-chloro-4-fluorophenyl)alaninate* (**4f’**; R=3-Cl-4-F-C_6_H_3_): Under the same reaction conditions for **4f** except that catalyst **1f** was replaced with **1i**, enantiomer **4f’** was obtained as colorless liquid. 88% yield, 97% ee; [α${]}_{D}^{20}$ −170.0° (*c* = 1.2, CH_2_Cl_2_).

*(R)-tert-Butyl N-(diphenylmethylene)-(3-bromo-5-fluorophenyl)alaninate* (**4g**; R=3-Br-5-F-C_6_H_3_): Under the same reaction conditions for **4a** except that 3,5-dichlorophenyl bromide was replaced with 3**-**bromo-5-fluorophenyl bromide, **4g** was obtained as colorless liquid. 83% yield; 96% ee; [α${]}_{D}^{20}$ 151.7° (*c* = 1.3, CH_2_Cl_2_); ^1^H-NMR (400 MHz, CDCl_3_): δ 7.57 (d, *J* = 7.6 Hz, 2H), 7.41–7.29 (m, 6H), 7.05 (d, *J* = 8.0 Hz, 1H), 7.00 (s, 1H), 6.75 (d, *J* = 8.4 Hz, 3H), 4.12 (q, *J* = 4.4 Hz, 1H), 3.21–3.09 (m, 2H), 1.45 (s, 9H); ^13^C-NMR (100 MHz, CDCl_3_): δ 171.1, 170.3, 163.4, 161.2, 142.7, 142.6, 139.3, 136.3, 130.5, 130.2, 128.9, 128.8, 128.8, 128.7, 128.4, 128.1, 127.7, 122.1, 122.0, 117.1, 116.8, 116.0, 115.8, 81.7, 67.1, 39.1, 28.1; HRMS (ESI, positive): Calcd. for C_26_H_26_BrFNO_2_ [M + H]^+^ 482.1125, found: 482.1119. HPLC analysis: Daicel Chiralcel OD-H, *n*-hexane/isopropanol = 95:5, flow rate = 0.5 mL/min.

*(S)-tert-Butyl N-(diphenylmethylene)-(3-bromo-5-fluorophenyl)alaninate* (**4g’**; R=3-Br-5-F-C_6_H_3_): Under the same reaction conditions for **4g** except that catalyst **1f** was replaced with **1i**, enantiomer **4g’** was obtained as colorless liquid. 86% yield, 98% ee; [α${]}_{D}^{20}$ −154.4° (*c* = 1.3, CH_2_Cl_2_).

*(R)-tert-Butyl N-(diphenylmethylene)-(3,5-dimethoxyphenyl)alaninate* (**4h**; R=3,5-(MeO)_2_-C_6_H_3_): Under the same reaction conditions for **4a** except that 3,5-dichlorophenyl bromide was replaced with 3,5-dimethoxybenzyl bromide, **4h** was obtained as colorless liquid. 85% yield; 96% ee; [α${]}_{D}^{29}$ 160.2° (*c* = 1.0, CH_2_Cl_2_); ^1^H-NMR (400 MHz, CDCl_3_): δ 7.60 (s, 1H), 7.58 (d, *J* = 1.4 Hz, 1H), 7.38–7.26 (m, 6H), 6.65 (d, *J* = 1.7 Hz, 2H), 6.27 (q, *J* = 2.2 Hz, 1H), 6.21 (d, *J* = 2.2 Hz, 2H), 4.12 (dd, *J* = 9.3, 4.2 Hz, 1H), 3.63 (s, 6H), 3.19–3.08 (m, 2H), 1.46 (s, 9H); ^13^C-NMR (100 MHz, CDCl_3_): δ 170.8, 170.2, 160.4, 140.5, 139.5, 136.3, 130.1, 128.7, 128.2, 127.9, 127.9, 127.7, 107.4, 99.1, 81.3, 67.7, 55.1, 39.8, 28.0; HRMS (ESI, positive): Calcd. for C_28_H_32_NO_4_ [M + H]^+^ 446.2326, found: 446.2327. HPLC analysis: Daicel Chiralcel IA, *n*-hexane/isopropanol = 95:5, flow rate = 0.5 mL/min.

*(S)-tert-Butyl N-(diphenylmethylene)-(3,5-dimethoxyphenyl)alaninate* (**4h’**; R=3,5-(MeO)_2_-C_6_H_3_): Under the same reaction conditions for **4h** except that catalyst **1f** was replaced with **1i**, enantiomer **4h’** was obtained as colorless liquid. 98% yield, 98% ee; [α${]}_{D}^{29}$ −211.4° (*c* = 1.0, CH_2_Cl_2_).

*(R)-tert-Butyl N-(diphenylmethylene)-(3-fluorophenyl)alaninate* (**4i**; R=3-F-C_6_H_4_): Under the same reaction conditions for **4a** except that 3,5-dichlorophenyl bromide was replaced with 3-fluorobenzyl bromide, **4i** was obtained as colorless liquid. 77% yield; 95% ee (Lit 90% ee [34]); [α${]}_{D}^{29}$ 169.2° (*c* = 1.1, CH_2_Cl_2_); ^1^H-NMR (400 MHz, CDCl_3_): δ 7.57 (d, *J* = 7.2 Hz, 2H), 7.38–7.24 (m, 6H), 7.14 (dd, *J* =14.1, 7.8 Hz, 1H), 6.86–6.83 (m, 2H), 6.75 (d, *J* = 9.9 Hz, 1H), 6.69 (d, *J* = 6.3 Hz, 2H), 4.12 (dd, *J* = 9.0, 4.4 Hz, 1H), 3.25–3.13 (m, 2H), 1.44 (s, 9H); ^13^C-NMR (75 MHz, CDCl_3_): δ 170.6, 170.5, 163.8, 161.4, 140.9, 140.8, 139.3, 136.2, 130.2, 129.4, 129.4, 128.7, 128.3, 128.1, 127.9, 127.6, 125.5, 125.5, 116.6, 116.4, 113.1, 112.9, 81.3, 67.5, 39.2, 28.0. HRMS (ESI, positive): Calcd. for C_26_H_26_FNNaO_2_ [M + Na]^+^ 426.1840, found: 426.1846. HPLC analysis: Daicel Chiralcel AD-H, *n*-hexane/isopropanol = 97:3, flow rate = 0.5 mL/min.

*(S)-tert-Butyl N-(diphenylmethylene)-(3-fluorophenyl)alaninate* (**4i’**; R=3-F-C_6_H_4_): Under the same reaction conditions for **4i** except that catalyst **1f** was replaced with **1i**, enantiomer **4i’** was obtained as colorless liquid. 82% yield; 95% ee (Lit 96% ee [35]); [α${]}_{D}^{29}$ −156.7° (*c* = 0.9, CH_2_Cl_2_).

*(R)-tert-Butyl N-(diphenylmethylene)-(3-chlorophenyl)alaninate* (**4j**; R=3-Cl-C_6_H_4_): Under the same reaction conditions for **4a** except that 3,5-dichlorophenyl bromide was replaced with 3-chlorobenzyl bromide, **4j** was obtained as light yellow liquid. 76% yield; 96% ee (Lit 95% ee [30]); [α${]}_{D}^{29}$ 227.4° (*c* = 1.0, CH_2_Cl_2_); ^1^H-NMR (400 MHz, CDCl_3_): δ 7.56 (d, *J* = 7.2 Hz, 2H), 7.38–7.25 (m, 6H), 7.15–7.09 (m, 2H), 7.02 (s, 1H), 6.97 (d, *J* = 7.0 Hz, 2H), 6.67 (d, *J* = 6.3 Hz, 2H), 4.11 (dd, *J* = 9.0, 4.4 Hz, 1H), 3.22–3.11 (m, 2H), 1.45 (s, 9H); ^13^C-NMR (100 MHz, CDCl_3_): δ 170.7, 170.5, 140.4, 139.3, 136.2, 133.8, 130.2, 129.8, 129.3, 128.7, 128.4, 128.1, 128.1, 128.0, 127.6, 126.3, 81.3, 67.4, 39.1, 28.0; HRMS (ESI, positive): Calcd. for C_26_H_27_ClNO_2_ [M + H]^+^ 420.1725, found: 420.1724. HPLC analysis: Daicel Chiralcel OD-H, *n*-hexane/isopropanol = 95:5, flow rate = 0.5 mL/min.

*(S)-tert-Butyl N-(diphenylmethylene)-(3-chlorophenyl)alaninate* (**4j’**; R=3-Cl-C_6_H_4_): Under the same reaction conditions for **4j** except that catalyst **1f** was replaced with **1i**, enantiomer **4j’** (1.6 g, 92% yield) was obtained as light yellow oil, and used for synthesis of **13**. 97% ee (Lit 91% ee [36], Lit 92% ee [37,38]); [α${]}_{D}^{29}$ −223.4° (*c* = 1.0, CH_2_Cl_2_); [Lit [α${]}_{D}^{20}$ −16.3° (*c* = 0.2, CHCl_3_) [30]].

*(R)-tert-Butyl N-(diphenylmethylene)-(3-bromophenyl)alaninate* (**4k**; R=3-Br-C_6_H_4_): Under the same reaction conditions for **4a** except that 3,5-dichlorophenyl bromide was replaced with 3-bromobenzyl bromide, **4k** was obtained as colorless liquid. 98% yield, 95% ee (Lit 92% ee [30]); [α${]}_{D}^{29}$ 185.4° (*c* = 1.0, CH_2_Cl_2_); ^1^H-NMR (400 MHz, CDCl_3_): δ 7.56 (d, *J* = 7.0 Hz, 2H), 7.36–7.30 (m, 7H), 7.18 (s, 1H), 7.07–7.03 (m, 2H), 6.67 (s, 2H), 4.12–4.09 (m, 1H), 3.21–3.10 (m, 2H), 1.45 (s, 9H); ^13^C-NMR (400 MHz, CDCl_3_): δ 170.7, 170.4, 140.7, 139.3, 136.2, 132.7, 130.2, 129.6, 129.2, 128.7, 128.6, 128.4, 128.2, 128.0, 127.5, 122.1, 81.4, 67.4, 39.1, 18.0; HRMS (ESI, positive): Calcd. for C_26_H_26_BrNaNO_2_ [M + Na]^+^ 486.1039, found: 486.1040. HPLC analysis: Daicel Chiralcel OD-H, *n*-hexane/isopropanol = 98:2, flow rate = 0.5 mL/min.

*(S)-tert-Butyl N-(diphenylmethylene)-(3-bromophenyl)alaninate* (**4k’**; R=3-Br-C_6_H_4_): Under the same reaction conditions for **4k** except that catalyst **1f** was replaced with **1i**, enantiomer **4k’** was obtained as colorless liquid. 93% yield, 93% ee; [α${]}_{D}^{29}$ −188.2° (*c* = 1.0, CH_2_Cl_2_).

*(R)-tert-Butyl N-(diphenylmethylene)-(4-bromophenyl)alaninate* (**4l**; R=4-Br-C_6_H_4_): Under the same reaction conditions for **4a** except that 3,5-dichlorophenyl bromide was replaced with 4-bromobenzyl bromide, **4l** was obtained as colorless liquid. 95% yield; 95% ee; [α${]}_{D}^{29}$ 127.2° (*c* = 1.0, CH_2_Cl_2_); ^1^H-NMR (400 MHz, CDCl_3_): δ 7.58 (s, 1H), 7.56 (d, *J* = 1.5 Hz, 2H), 7.40–7.29 (m, 8H), 6.93 (d, *J* = 8.3 Hz, 2H), 6.67 (d, *J* = 6.4 Hz, 2H), 4.09 (dd, *J* = 9.0, 4.4 Hz, 1H), 3.19–3.08 (m, 2H), 1.44 (s, 9H); ^13^C-NMR (300 MHz CDCl_3_): δ 170.6, 170.5, 139.3, 137.4, 136.2, 131.6, 130.2, 128.7, 128.3, 128.1, 128.0, 127.6, 120.0, 81.3, 67.5, 38.9, 28.0; HRMS (ESI, positive): Calcd. for C_26_H_27_BrNO_2_ [M + H]^+^ 464.1220, found: 464.1220. HPLC analysis: Daicel Chiralcel OD-H, *n*-hexane/isopropanol = 98:2, flow rate = 0.5 mL/min.

*(S)-tert-Butyl N-(diphenylmethylene)-(4-bromophenyl)alaninate* (**4l’**; R=4-Br-C_6_H_4_): Under the same reaction conditions for **4l** except that catalyst **1f** was replaced with **1i**, enantiomer **4l’** was obtained as colorless liquid. 95% yield; 95% ee (Lit 80% ee [32]); [α${]}_{D}^{29}$ −134.4° (*c* = 1.0, CH_2_Cl_2_); [Lit [α${]}_{D}^{27}$ −110.1° (*c* = 1.09, CHCl_3_) [32]].

*(R)-tert-Butyl N-(diphenylmethylene)-(3-iodophenyl)alaninate* (**4m**; R=3-I-C_6_H_4_): Under the same reaction conditions for **4a** except that 3,5-dichlorophenyl bromide was replaced with 3-iodobenzyl bromide, **4m** was obtained as colorless liquid. 82% yield, 96% ee; [α${]}_{D}^{29}$ 235.6° (*c* = 1.0, CH_2_Cl_2_); ^1^H-NMR (400 MHz, CDCl_3_): δ 7.56 (d, *J* = 7.2 Hz, 2H), 7.49 (d, *J* = 7.8 Hz, 1H), 7.38–7.25 (m, 7H), 7.05 (d, *J* = 7.6 Hz, 1H), 6.92 (t, *J* = 7.7 Hz, 1H), 6.65 (d, *J* = 5.3 Hz, 2H), 4.09 (dd, *J* = 4.8, 4.4 Hz, 1H), 3.18–3.07 (m, 2H), 1.45 (s, 9H); ^13^C-NMR (100 MHz, CDCl_3_): δ 170.7, 170.4, 140.8, 139.3, 138.6, 136.2, 135.2, 130.2, 129.8, 129.2, 128.7, 128.3, 128.2, 127.9, 127.6, 94.1, 81.4, 67.4, 39.0, 28.0; HRMS: Calcd. for C_26_H_27_INO_2_^+^ [M + H]^+^ 512.1081, found: 512.1083. HPLC analysis: Daicel Chiralcel OD-H, *n*-hexane/isopropanol = 95:5, flow rate = 0.5 mL/min.

*(S)-tert-Butyl N-(diphenylmethylene)-(3-iodophenyl)alaninate* (**4m’**; R=3-I-C_6_H_4_): Under the same reaction conditions for **4m** except that catalyst **1f** was replaced with **1i**, enantiomer **4m’** was obtained as colorless liquid. 86% yield; 94% ee (Lit 95% ee [39]); [α${]}_{D}^{29}$ −191.4° (*c* = 1.0, CH_2_Cl_2_).

*(R)-tert-Butyl N-(diphenylmethylene)-(4-nitrophenyl)alaninate* (**4n**; R=4-NO_2_-C_6_H_4_): Under the same reaction conditions for **4a** except that 3,5-dichlorophenyl bromide was replaced with 4-nitrobenzyl bromide, **4n** was obtained as light yellow solid. M.p. 129–131 °C; 99% yield; 95% ee (Lit 90% ee [30]); [α${]}_{D}^{29}$ 184.8° (*c* = 1.0, CH_2_Cl_2_); ^1^H-NMR (400 MHz, CDCl_3_): δ 8.06 (dd, *J* = 7.0, 1.8 Hz, 2H), 7.58 (s, 1H), 7.56 (d, *J* = 1.5 Hz, 1H), 7.41–7.36 (m, 2H), 7.34–7.29 (m, 4H), 7.26 (d, *J* = 2.5 Hz, 1H), 7.24 (s, 1H), 6.71 (d, *J* = 6.8 Hz, 2H), 4.18 (dd, *J* = 8.1, 5.2 Hz, 1H), 3.35–3.25 (m, 2H), 1.45 (s, 9H); ^13^C-NMR (100 MHz, CDCl_3_): δ 170.9, 170.0, 146.5, 146.4, 139.0, 135.9, 130.6, 130.4, 128.7, 128.6, 128.3, 128.0, 127.4, 123.2, 81.6, 66.9, 39.3, 28.0; HRMS (ESI, positive): Calcd. for C_26_H_27_N_2_O_4_ [M + H]^+^ 431.1965, found: 431.1962. HPLC analysis: Daicel Chiralcel IA, *n*-hexane/isopropanol = 94:6, flow rate = 0.5 mL/min.

*(S)-tert-Butyl N-(diphenylmethylene)-(4-nitrophenyl)alaninate* (**4n’**; R=4-NO_2_-C_6_H_4_): Under the same reaction conditions for **4n** except that catalyst **1f** was replaced with **1i**, enantiomer **4n’** was obtained as light yellow solid. 96% yield; 94% ee (Lit 99% ee [39]); [α${]}_{D}^{29}$ −165.4° (*c* = 1.0, CH_2_Cl_2_).

*(R)-tert-Butyl 2-((diphenylmethylene)amino)-3-(1-naphthyl)propanoate* (**4o**; R=α-naphthyl): Under the same reaction conditions for **4a** except that 3,5-dichlorophenyl bromide was replaced with 1-(bromomethyl)naphthalene, **4o** was obtained as colorless liquid. 81% yield; 96% ee (Lit 96% ee [40], Lit 99% ee [33]); [α${]}_{D}^{29}$ 331.6° (*c* = 1.0, CH_2_Cl_2_); [Lit [α${]}_{D}^{27}$ 343.7° (*c* = 1.19, CHCl_3_) [33]]; ^1^H-NMR (400 MHz, CDCl_3_): δ 7.78 (d, *J* = 8.0 Hz, 1H), 7.72–7.67 (m, 2H), 7.51 (d, *J* =7.6 Hz, 2H), 7.38 (t, *J* = 7.4 Hz, 1H), 7.33–7.22 (m, 6H), 7.11 (t, *J* = 7.5 Hz, 1H), 6.95 (t, *J* = 7.5 Hz, 2H), 6.24 (s, 2H), 4.32 (dd, *J* = 9.5, 4.0 Hz, 1H), 3.80 (dd, *J* = 13.7, 4.0 Hz, 1H), 3.50 (dd, *J* = 13.7, 9.6 Hz, 1H), 1.46 (s, 9H); ^13^C-NMR (75 MHz, CDCl_3_): δ 171.0, 170.2, 139.3, 135.8, 134.1, 133.6, 132.2, 130.0, 128.6, 128.4, 128.2, 128.2, 127.8, 127.6, 127.2, 127.0, 125.6, 125.2, 125.2, 123.6, 81.1, 66.5, 36.6, 28.0; HRMS (ESI, positive): Calcd. for C_30_H_29_NNaO_2_^+^ [M + Na]^+^ 458.2091, found: 458.2091. HPLC analysis: Daicel Chiralcel AD-H, *n*-hexane/isopropanol = 97:3, flow rate = 0.5 mL/min.

*(S)-tert-Butyl 2-((diphenylmethylene)amino)-3-(1-naphthyl)propanoate* (**4o’**; R=α-naphthyl): Under the same reaction conditions for **4o** except that catalyst **1f** was replaced with **1i**, enantiomer **4o’** was obtained as colorless liquid. 85% yield; 97% ee (Lit 98% ee [41]); [α${]}_{D}^{29}$ −295.3° (*c* = 0.9, CH_2_Cl_2_).

*(S)-3-Chloro phenylalanine hydrochloride* (**5**): A mixture of **4j’** (1.2 g, 2.8 mmol) and 6 M HCl (6 mL) was heated at 100 °C for 3 h, and then cooled to ambient temperature, resulting in white precipitates. **5** (525 mg, 92% yield) was obtained by suction filtration as white solid, and was used for the next step without further purification. M.p. 264–266 °C; ^1^H-NMR (300 MHz, DMSO-*d*_6_): δ 3.18 (d, *J* = 6.2 Hz, 2H), 4.17 (s, 1H), 7.26–7.39 (m, 4H), 8.62 (s, 3H), 13.88 (s, 1H); ^13^C-NMR (75 MHz, DMSO-*d*_6_): δ 35.1, 52.9, 127.2, 128.5, 129.5, 130.4, 133.1, 137.7, 170.2; HRMS (ESI, positive): calcd. for C_9_H_11_ClNO_2_ [M + H]^+^ 200.0473, found: 200.0474.

*(9H-Fluoren-9-yl)methyl (S)-(3-chloro)phenylalanine* (**6**): To an ice-cold solution of **5** (525 mg, 2.6 mmol), dioxane (3 mL) and 10% Na_2_CO_3_ aqueous solution (6 mL) was added dropwise a solution of Fmoc-Cl (673 mg, 2.6 mmol) in dioxane (3 mL). The mixture was stirred at 0 °C for 4 h, and then warmed to ambient temperature with the stirring continued for an additional 18 h. The reaction was quenched by adding 2 M HCl (5 mL) and H_2_O (40 mL). The resulting mixture was extracted with EtOAc (2 × 60 mL), and the combined extracts were washed with brine (2 × 30 mL) and dried. The crude product was purified by flash column chromatography (eluting with hexane/EtOAc, 20:1) to give **6** (644 mg, 59% yield) as white solid. M.p. 123–125 °C; ^1^H-NMR (300 MHz, DMSO-*d*_6_): δ 2.88 (q, *J* = 7.7 Hz, 1H), 3.17 (s, 1H), 4.09–4.17 (m, 3H), 4.25 (q, *J* = 4.8 Hz, 1H), 7.20–7.39 (m, 9H), 7.61 (d, *J* = 7.4 Hz, 2H), 7.87 (d, *J* = 7.5 Hz, 2H); ^13^C-NMR (75 MHz, DMSO-*d*_6_): δ 36.5, 46.6, 55.9, 65.6, 120.2, 125.2, 125.4, 126.3, 127.1, 127.7, 128.0, 129.2, 129.9, 132.7, 140.6, 140.7, 141.2, 143.8, 143.9, 155.9, 173.9; HRMS (ESI, positive): calcd. for C_24_H_20_ClNO_4_Na [M + Na]^+^ 444.0973, found: 444.0967.

*(9H-Fluoren-9-yl)methyl(S)-(3-(3-chlorophenyl)-1-(methylamino)-1-oxopropan-2-yl)carbamate* (**7**): Methyl-amine hydrochloride (202 mg, 3 mmol) and *N,N*-diisopropylethylamine (DIEA, 388 mg, 3 mmol) was added successively to an ice-cold stirred solution of the substituted **6** (624 mg, 1.5 mmol), HOBt (405 mg, 3 mmol) and HBTU (1.1 g, 3 mmol) in DMF (6 mL) at 0 °C. The reaction mixture was stirred for 30 min at the same temperature, and then allowed to warm to ambient temperature while the stirring continued for an additional 12 h. The solvents and volatiles were removed under reduced pressure, and the residue was dissolved in EtOAc (150 mL) and then washed with saturated NaHCO_3_ solution (50 mL) and brine (2 × 50 mL) and finally dried (anhydrous Na_2_SO_4_). After the solvent was concentrated, the crude product was crystallized from EtOAc to give **7** (504 mg, 77% yield) as white solid. M.p. 179–181 °C; 96% ee; [α${]}_{D}^{21}$ −0.6° (*c* = 0.7, CH_2_Cl_2_); ^1^H-NMR (300 MHz, DMSO-*d*_6_): δ 2.57 (d, *J* = 4.5 Hz, 3H), 2.76–2.98 (m, 4H), 3.16 (s, 1H), 4.46 (d, *J* = 5.0 Hz, 1H), 7.14–7.29 (m, 5H), 7.38 (d, *J* = 1.7 Hz, 2H), 7.76 (d, *J* = 8.2 Hz, 2H), 7.91 (d, *J* = 4.3 Hz, 2H), 8.12 (d, *J* = 7.8 Hz, 1H); ^13^C-NMR (75 MHz, DMSO-*d*_6_): δ 26.0, 37.6, 47.0, 56.6, 66.1, 120.6, 125.7, 125.8, 126.8, 127.5, 128.1, 128.4, 129.6, 130.3, 133.1, 141.0, 141.1, 141.4, 144.2, 144.2, 156.3, 172.1; HRMS (ESI, positive): calcd. for C_25_H_24_ClN_2_O_3_ [M + H]^+^ 435.1470, found: 435.1470. HPLC analysis: Daicel Chiralcel OD-H, *n*-hexane/isopropanol = 85:15, flow rate = 1.0 mL/min.

*(S)-2-Amino-3-chloro-N-methylpropanamides* (**8**): To a stirred solution of **7** (480 mg, 1.1 mmol) in DMF (4 mL) was added piperidine (2 mL) at room temperature. The reaction mixture was stirred at ambient temperature and under nitrogen atmosphere for 2 h. The solvent and volatiles were removed under reduced pressure, and the residue was purified by flash column chromatography (eluting with DCM/MeOH, 20:1) to afford **8** (210 mg, 90% yield) as light yellow liquid; ^1^H-NMR (300 MHz, DMSO-*d*_6_): δ 7.91 (d, *J* = 4.5 Hz, 1H), 7.32–7.23 (m, 3H), 7.16 (m, 1H), 3.42 (dd, *J* = 8.1, 5.2 Hz, 1H), 2.92 (dd, *J* = 13.4, 5.1 Hz, 1H), 2.77 (s, 2H), 2.65 (dd, *J* = 13.4, 5.1 Hz, 1H), 2.58 (d, *J* = 4.7 Hz, 3H); ^13^C-NMR (75 MHz, DMSO-*d*_6_): δ 174.3, 141.4, 132.9, 130.0, 129.3, 128.2, 126.3, 56.1, 40.5, 25.6; HRMS (ESI, positive): calcd. for C_10_H_14_ClN_2_O [M + H]^+^ 213.0789, found: 213.0789.

*(9H-Fluoren-9-yl)methyl((S)-1-(((S)-3-(3-chlorophenyl)-1-(methylamino)-1-oxopropan-2-yl)amino)-3-(4-carbamoylphenyl)-1-oxopropan-2-yl)carbamate* (**10**): HOBt (126 mg, 0.9 mmol) and HBTU (354 mg, 0.9 mmol) were added to a stirred solution of **9** (0.52 mmol) in DMF (6 mL) at rt. After the mixture was cooled to 0 °C, **8** (165 mg, 0.8 mmol) and DIEA (1 mmol) were introduced. After the whole reaction mixture was stirred at rt for 12 h, the solvents and volatiles were removed under reduced pressure. The solid residue was crystallized from dichloromethane to give **10** (350 mg, 51% yield) as white solid. M.p. 238–240 °C; ^1^H-NMR (300 MHz, DMSO-*d*_6_): δ 2.57 (d, *J* = 4.3 Hz, 3H), 2.68–2.88 (m, 2H), 2.95–3.01 (m, 2H), 4.00–4.31 (m, 4H), 4.44–4.51 (m, 1H), 7.16–7.42 (m, 12H), 7.60 (t, *J* = 7.57 Hz, 3H), 7.78–7.93 (m, 6H), 8.24 (d, *J* = 8.2 Hz, 1H); ^13^C-NMR (75 MHz, DMSO-*d*_6_): δ 25.6, 37.4, 37.7, 46.6, 53.9, 56.0, 65.8, 120.2, 125.3, 125.4, 126.5, 127.2, 127.4, 127.7, 128.1, 129.1, 130.0, 132.4, 132.8, 140.3, 140.8, 141.6, 143.8, 143.9, 155.8, 167.9, 171.0, 171.3; HRMS (ESI, positive): calcd. for C_35_H_33_ClN_4_O_3_Na [M + Na]^+^ 647.2032, found: 647.2035.

*4-((S)-2-Amino-3-(((S)-3-(3-chlorophenyl)-1-(methylamino)-1-oxopropan-2-yl)amino)-3-oxopropyl)benzamide* (**11**): Piperidine (2 mL) was added to a stirred solution of **10** (330 mg, 0.5 mmol) dissolved in DMF (4 mL), and the mixture was stirred at rt for 2 h. After the completion of the reaction, the solvent and volatiles were removed under reduced pressure, and the solid residue was crystallized from EtOAc to give **11** as light yellow solid (200 mg, 95% yield). M.p. 237–239 °C; ^1^H-NMR (300 MHz, DMSO-*d*_6_): δ 2.54 (t, *J* = 10.5 Hz, 3H), 2.79–3.39 (m, 3H), 4.46 (s, 1H), 7.12–7.31 (m, 6H), 7.77 (d, *J* = 7.8 Hz, 2H), 7.99 (t, *J* = 9.1 Hz, 2H), 8.20 (d, *J* = 5.8 Hz, 1H); ^13^C-NMR (75 MHz, DMSO-*d*_6_): δ 25.6, 37.6, 53.3, 56.2, 126.4, 127.4, 128.1, 129.1, 129.2, 129.9, 132.2, 132.7, 140.4, 142.2, 167.8, 171.0, 174.0; HRMS (ESI, positive): calcd. for C_20_H_23_ClN_4_O_3_Na [M + Na]^+^ 245.1351, found: 245.1350.

*(2S)-3-(3-Chlorophenyl)-2-((2S)-2-(2-cyclohexyl-2-phenylacetamido)-3-phenylpropan-amido)-N-methyl-propanamide* (**13**): HOBt (114 mg, 0.8 mmol) and HBTU (320 mg, 0.8 mmol) were added to a stirred solution of **12** (0.8 mmol) in DMF (4 mL) at rt. After the mixture was cooled to 0 °C, **11** (170 mg, 0.4 mmol) was added, followed by addition of DIEA (1.2 mmol). The reaction mixture was stirred at ambient temperature for 12 h, the solvent and volatiles were evaporated under reduced pressure, and then the solid residue was crystallized from EtOAc to generate **13** (160 mg, 63% yield) as white solid. M.p. 243–245 °C; ^1^H-NMR (300 MHz, DMSO-*d*_6_): δ 0.56–0.64 (m, 3H), 0.81–0.93 (m, 6H), 1.06–1.59 (m, 3H), 2.55 (d, *J* = 4.5 Hz, 3H), 2.68–3.06 (m, 3H), 3.19 (d, *J* = 10.8 Hz, 1H), 4.39–4.44 (m, 2H), 6.96 (d, *J* = 8.4 Hz, 1H), 7.18–7.33 (m, 7H), 7.52 (d, *J* = 8.1 Hz, 1H), 7.77–7.94 (m, 3H), 8.21–8.25 (m, 3H); ^13^C-NMR (75 MHz, DMSO-*d*_6_): δ 24.9, 25.2, 25.9, 26.0, 30.8, 37.7, 39.1, 42.7, 54.1, 57.3, 121.8, 126.8, 127.5, 128.6, 128.7, 129.3, 129.7, 130.6, 132.2, 132.5, 132.6, 140.8, 141.3, 141.7, 168.1, 171.1, 171.2, 173.0; HRMS (ESI, positive): calcd. for C_34_H_40_ClN_4_O_4_ [M + H]^+^ 603.2733, found: 603.2730.

The ^1^H and ^13^C-NMR spectra of the compounds are available in Supplementary Materials.

## 4. Conclusions

In summary, we have developed a practical method for synthesizing both enantiomers of unnatural α-amino acid derivatives by asymmetric α-alkylation of *N*-(dibenzylidene)glycine *tert*-butyl ester (**2**) with substituted benzyl bromides and 1-(bromomethyl)naphthalene under the catalysis of *O*-allyl-*N*-(9-anthracenmethyl) cinchodium bromide (**1f**) and *O*-allyl-*N*-(9-anthracenmethyl) bromide (**1i**), respectively. A series of both (*R*)- and (*S*)-enantiomers of unnatural α-amino acid derivatives were obtained in good to excellent yields and with excellent enantioselectivity, and the procedure is simple, mild and scalable. Furthermore, the stereochemistry of the products is fully predictable and controlled: the cinchonine-type phase transfer catalyst **1f** resulted in all (*R*)-α-amino acid derivatives, whereas the cinchonidine-type phase transfer catalyst **1i** afforded the (*S*)-α-amino acid derivatives. Both resulting enantiomers of the substituted α-phenylalanine derivatives have been used for synthesizing new allosteric antagonists for β_2_AR.

## Figures and Tables

**Table 1:**
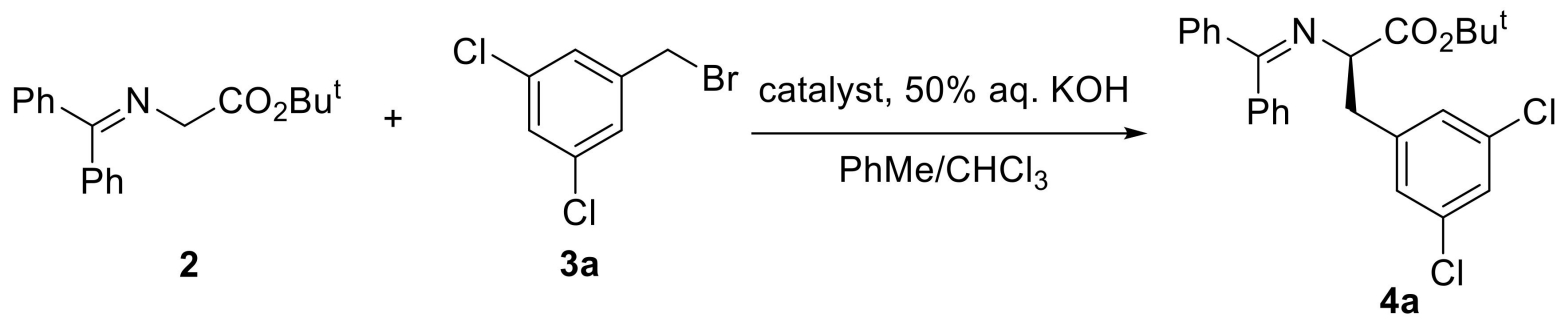
Optimization of the reaction conditions.

Entry *^a^*	1	Temp	Time/h	Yield *^b^*	ee *^c^*
1	**1a**	rt	24	81%	88%
2	**1b**	rt	24	77%	81%
3	**1c**	rt	24	93%	77%
4	**1d**	rt	24	97%	82%
5	**1e**	rt	24	72%	54%
6	**1f**	rt	24	94%	94%
7	**1g**	rt	24	74%	78%
8	**1h**	rt	24	99%	81%
9	**1f**	−20 °C	48	97%	96%
10	**1f**	−40 °C	72	95%	97%
11	**1f**	−60 °C	72	87%	92%
12*^d^*	**1f**	−40 °C	72	89%	96%
13*^e^*	**1f**	−40 °C	60	99%	96%

*^a^* Reactions were performed with **2** (0.1 mmol), **3a** (0.5 mmol), base (0.5 mmol) and **1** (0.01 mmol) in toluene/CHCl_3_. *^b^* Isolated yield. *^c^* Enantiomeric excess was determined by HPLC analysis using a chiral column with *n*-hexane–isopropanol as eluent. *^d^* Reactions were performed with **2** (0.1 mmol), **3a** (0.5 mmol), base (0.5 mmol) and **1** (0.005 mmol). *^e^* Reactions were performed with **2** (0.1 mmol), **3a** (0.5 mmol), base (0.5 mmol) and **1** (0.02 mmol).

**Table 2:**
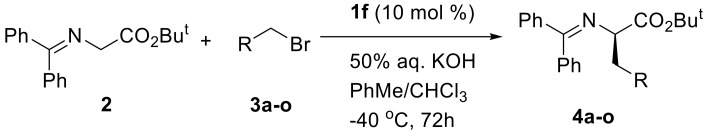
Asymmetric alkylation of **2** with **3a-o** under the catalysis of **1f**.

Entry *^a^*	R	Product	Yield *^b^*	ee *^c^*
1	3,5-Cl_2_C_6_H_3_	**4a**	95%	97% (*R*)
2	3,5-F_2_C_6_H_3_	**4b**	95%	94% (*R*)
3	3,5-Br_2_C_6_H_3_	**4c**	95%	93% (*R*)
4	3-Cl,5-FC_6_H_3_	**4d**	93%	97% (*R*)
5	2-Cl,6-FC_6_H_3_	**4e**	68%	98% (*R*)
6	3-Cl,4-FC_6_H_3_	**4f**	86%	97% (*R*)
7	3-Br,5-FC_6_H_3_	**4g**	83%	96% (*R*)
8	3,5-(MeO)_2_C_6_H_3_	**4h**	85%	96% (*R*)
9	3-FC_6_H_4_	**4i**	77%	95% (*R*)
10	3-ClC_6_H_4_	**4j**	76%	96% (*R*)
11	3-BrC_6_H_4_	**4k**	98%	95% (*R*)
12	4-BrC_6_H_4_	**4l**	95%	95% (*R*)
13	3-IC_6_H_4_	**4m**	82%	96% (*R*)
14	4-NO_2_C_6_H_4_	**4n**	99%	95% (*R*)
15	1-naphthyl	**4o**	81%	96% (*R*)

*^a^* Reactions were performed with **2** (0.1 mmol), alkylation reagent (0.5 mmol), 50% KOH (0.5 mmol) and **1f** (0.01 mmol) in toluene/CHCl_3_ at −40 °C. *^b^* Isolated yield. *^c^* Enantiomeric excess was determined by HPLC analysis using a chiral column with *n*-hexane–isopropanol as eluent.

**Table 3:**
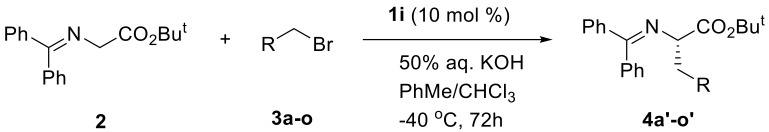
Asymmetric alkylation of **2** with **3a-o** under the catalysis of **1i**.

Entry *^a^*	R	Product	Yield*^b^*	ee *^c^*
1	3,5-Cl_2_C_6_H_3_	**4a’**	98%	97% (*S*)
2	3,5-F_2_C_6_H_3_	**4b’**	99%	94% (*S*)
3	3,5-Br_2_C_6_H_3_	**4c’**	83%	95% (*S*)
4	3-Cl,5-FC_6_H_3_	**4d’**	97%	98% (*S*)
5	2-Cl,6-FC_6_H_3_	**4e’**	74%	99% (*S*)
6	3-Cl,4-FC_6_H_3_	**4f’**	88%	97% (*S*)
7	3-Br,5-FC_6_H_3_	**4g’**	86%	98% (*S*)
8	3,5-(MeO)_2_C_6_H_3_	**4h’**	97%	98% (*S*)
9	3-FC_6_H_4_	**4i’**	82%	95% (*S*)
10	3-ClC_6_H_4_	**4j’**	71%	97% (*S*)
11	3-BrC_6_H_4_	**4k’**	93%	93% (*S*)
12	4-BrC_6_H_4_	**4l’**	95%	94% (*S*)
13	3-IC_6_H_4_	**4m’**	86%	94% (*S*)
14	4-NO_2_C_6_H_4_	**4n’**	96%	94% (*S*)
15	1-naphthyl	**4o’**	85%	97% (*S*)

*^a^* Reactions were performed with **2** (0.1 mmol), alkylation reagent (0.5 mmol), 50% KOH (0.5 mmol) and **1h** (0.01 mmol) in toluene/CHCl_3_ at −40 °C. *^b^* Isolated yield. *^c^* Enantiomeric excess was determined by HPLC analysis using a chiral column with *n*-hexane–isopropanol as eluent.

## Supplementary Materials

The supplementary materials containing NMR spectra and HPLC chromatograms can be accessed online.
