# Supplementary material for: Synthesis of Both Enantiomers of Chiral Phenylalanine Derivatives Catalyzed by Cinchona Alkaloid Quaternary Ammonium Salts as Asymmetric Phase Transfer Catalysts
Source: Molecules. 2018 Jun 12;23(6):1421. doi: 10.3390/molecules23061421 (PMC6099405; doi:10.3390/molecules23061421)

Supplementary Material
for

Synthesis of Both *Enantiomers of* Chiral Phenylalanine Derivatives Catalyzed by Cinchona Alkaloid Quaternary Ammonium Salts as Asymmetric Phase Transfer Catalysts

Lei Jin, Shuai Zhao, Xin Chen*

School of Pharmaceutical & Life Sciences, Changzhou University, Changzhou, Jiangsu 213164, China; 15102307@smail.cczu.edu.cn (L.J.); 1138971580@qq.com (S.Z.)

* Correspondence: xinchen@cczu.edu.cn (X.C.); Tel.: +86-519-86334597; Fax: +86-519-86334598

Received: date; Accepted: date; Published: date

Abstract: A practical synthesis of both enantiomers of unnatural phenylalanine derivatives by using two pseudoenantiomeric phase transfer catalysts is described. Through asymmetric α-alkylation of glycine Schiff base with substituted benzyl bromides and 1-(bromomethyl)naphthalene under the catalysis of *O*-allyl-*N*-(9-anthracenmethyl) cinchoninium bromide (**1f**) and *O*-allyl-*N*-(9-anthracenmethyl) cinchonidium bromide (**1i**), respectively, a series of both (*R*)- and (*S*)-*enantiomers of* unnatural α-amino acid derivatives were obtained in excellent yields and enantioselectivity. The synthetic method is simple and scalable, and the stereochemistry of the products is fully predictable and controlled: the cinchonine-type phase transfer catalyst **1f** resulted in (*R*)-α-amino acid derivatives, and the cinchonidine-type phase transfer catalyst **1i** afforded (*S*)-α-amino acid derivatives,

Keywords: both enantiomers, unnatural α-amino acids derivatives, phase transfer catalysts, asymmetric α-alkylation, glycine Schiff base

Contents

NMR spectra and HPLC chromatogram of **4a** and **4a'** 3
NMR spectra and HPLC chromatogram of **4b** and **4b'** 6
NMR spectra and HPLC chromatogram of **4c** and **4c'** 8
NMR spectra and HPLC chromatogram of **4d** and **4d'** 11
NMR spectra and HPLC chromatogram of **4e** and **4e'** 13
NMR spectra and HPLC chromatogram of **4f** and **4f'** 15
NMR spectra and HPLC chromatogram of **4g** and **4g'** 17
NMR spectra and HPLC chromatogram of **4h** and **4h'** 19
NMR spectra and HPLC chromatogram of **4i** and **4i'** 21
NMR spectra and HPLC chromatogram of **4j** and **4j'** 23
NMR spectra and HPLC chromatogram of **4k** and **4k'** 26
NMR spectra and HPLC chromatogram of **4l** and **4l'** 28
NMR spectra and HPLC chromatogram of **4m** and **4m'** 30
NMR spectra and HPLC chromatogram of **4n** and **4n'** 32
NMR spectra and HPLC chromatogram of **4o** and **4o'** 34

NMR spectra of **5** 36
NMR spectra of **6** 37
NMR spectra and HPLC chromatogram of **7** 38
NMR spectra of **8** 40
NMR spectra of **10** 41
NMR spectra of **11** 42
NMR spectra of **13** 43

NMR spectra and HPLC chromatogram of **4a** and **4a′**

^{1}H and ^{13}C NMR spectra of **4a** and **4a′**

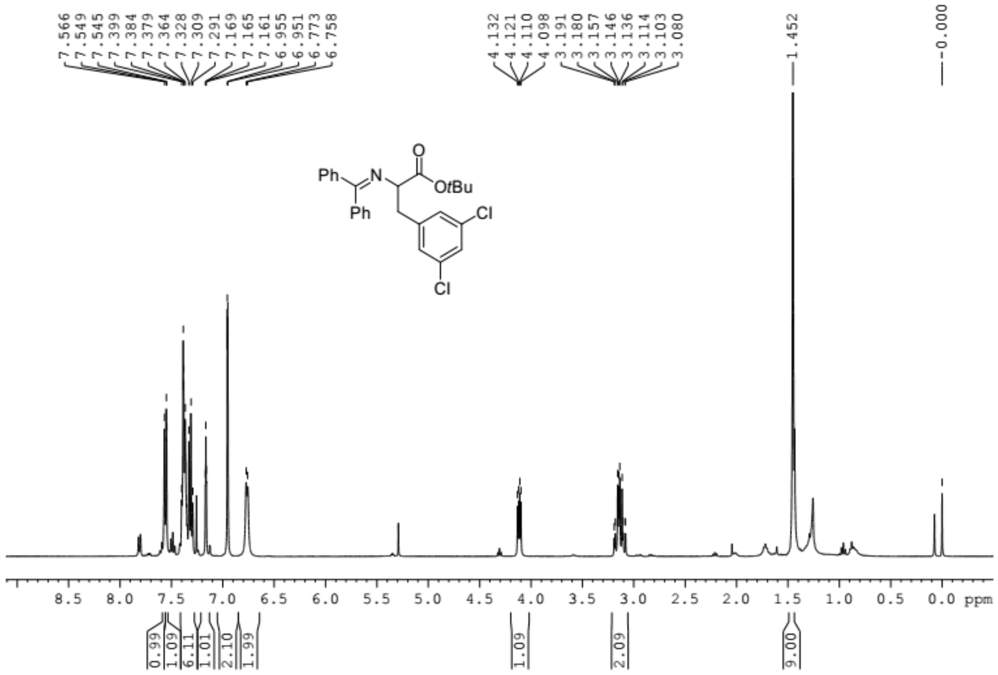

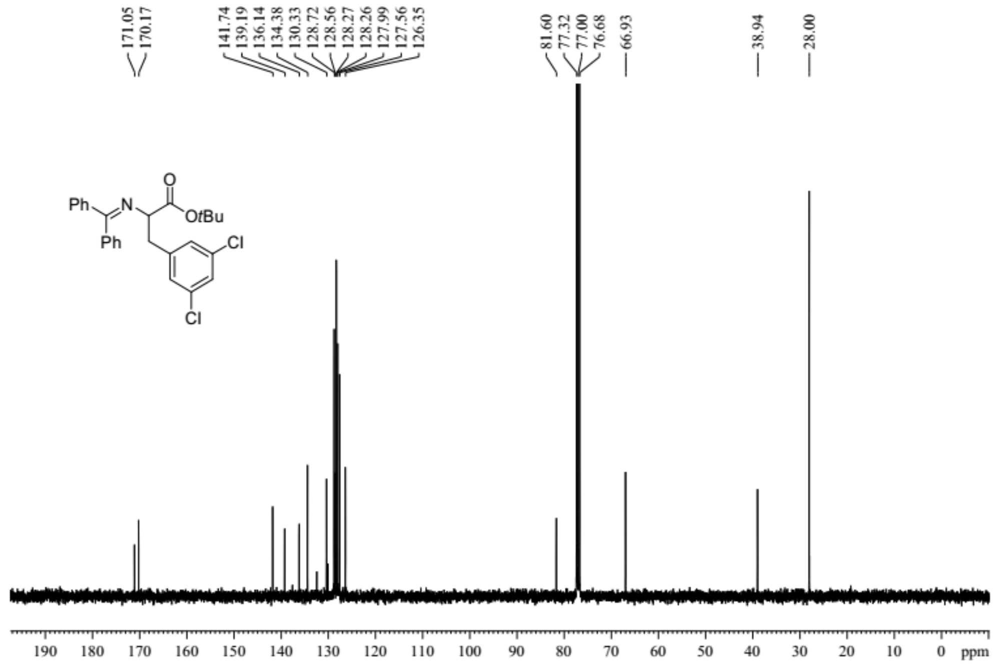

HPLC chromatogram of *rac*-**4a**

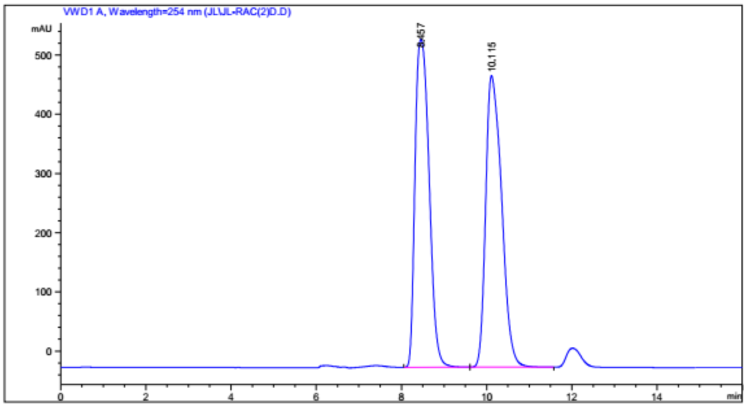

峰 #	保留时间 [min]	类型	峰宽 [min]	峰面积 [mAU*s]	峰高 [mAU]	峰面积 %
1	8.457	BB	0.3753	1.27014e4	554.03979	49.8460
2	10.115	BB	0.4208	1.27799e4	492.45120	50.1540

HPLC chromatogram of **4a**

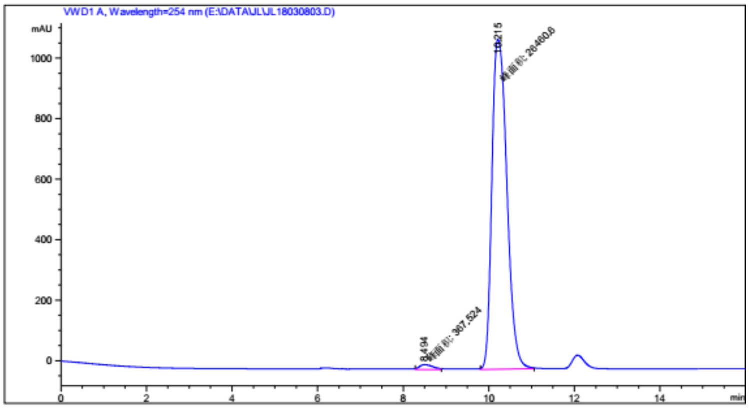

峰 #	保留时间 [min]	类型	峰宽 [min]	峰面积 [mAU*s]	峰高 [mAU]	峰面积 %
1	8.494	MM	0.3748	367.52402	16.34514	1.3699
2	10.215	MM	0.4047	2.64606e4	1089.68823	98.6301

HPLC chromatogram of *rac*-**4a′**

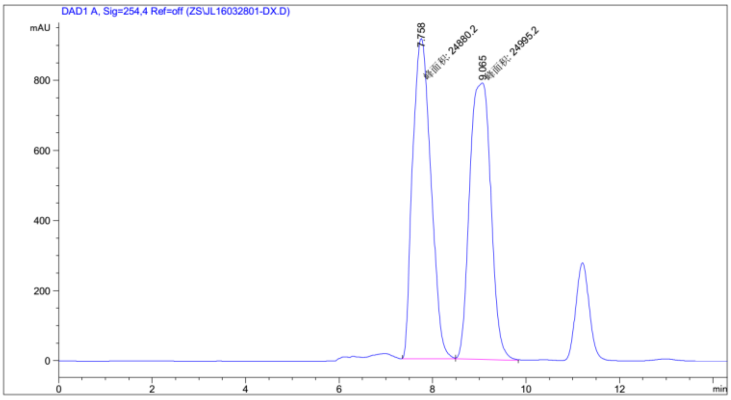

峰 #	保留时间 [min]	类型	峰宽 [min]	峰面积 [mAU*s]	峰高 [mAU]	峰面积 %
1	7.758	MM	0.4536	2.48802e4	914.18732	49.8848
2	9.065	MM	0.5278	2.49952e4	789.28033	50.1152

HPLC chromatogram of **4a′**

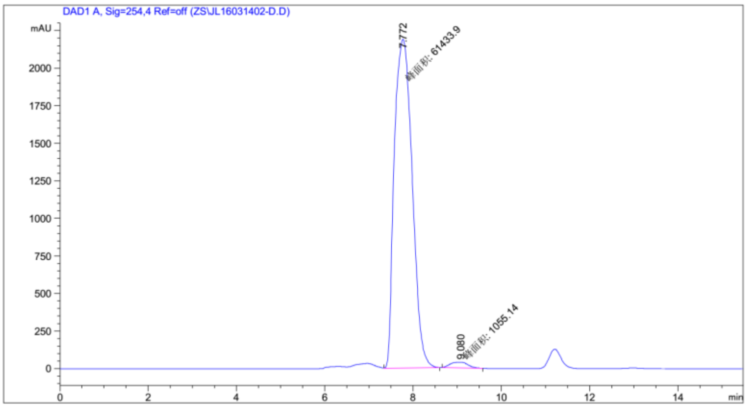

峰 #	保留时间 [min]	类型	峰宽 [min]	峰面积 [mAU*s]	峰高 [mAU]	峰面积 %
1	7.772	MM	0.4677	6.14339e4	2189.04443	98.3115
2	9.080	MM	0.4618	1055.13892	38.08363	1.6885

NMR spectra and HPLC chromatogram of **4b** and **4b′**

^{1}H and ^{13}C NMR spectra of **4b** and **4b′**

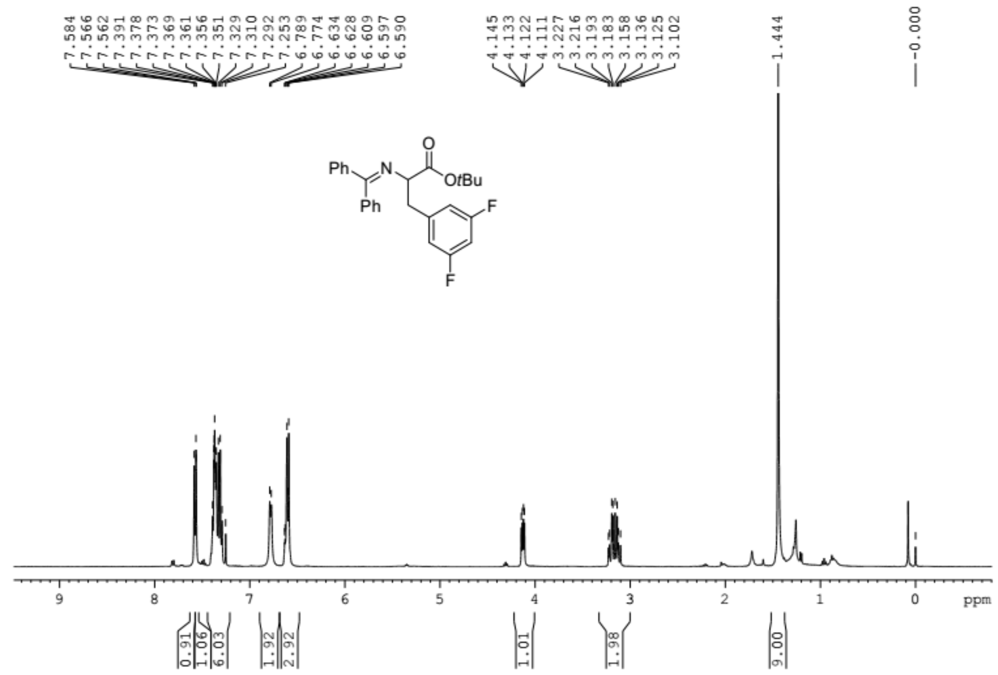

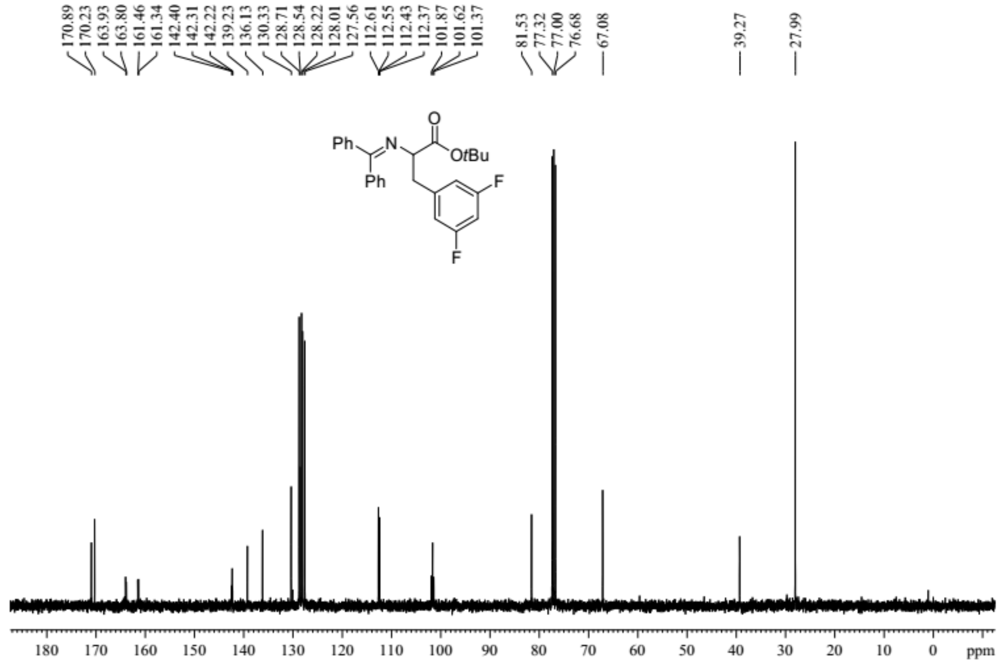

HPLC chromatogram of *rac*-**4b (4b′)**

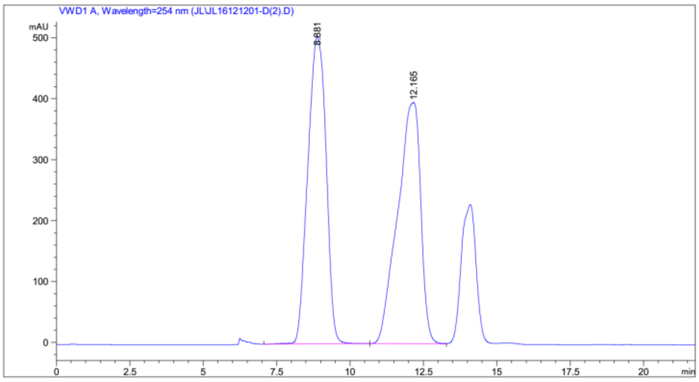

峰 #	保留时间 [min]	类型	峰宽 [min]	峰面积 [mAU*s]	峰高 [mAU]	峰面积 %
1	8.881	BB	0.7036	2.18291e4	503.69077	50.2237
2	12.165	BV	0.8019	2.16346e4	395.98453	49.7763

HPLC chromatogram of **4b′**

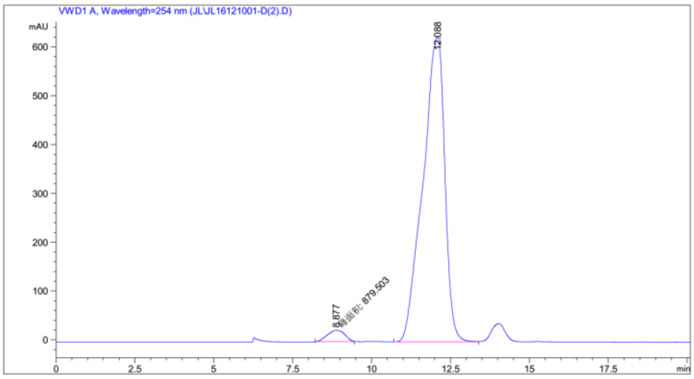

峰 #	保留时间 [min]	类型	峰宽 [min]	峰面积 [mAU*s]	峰高 [mAU]	峰面积 %
1	8.877	MM	0.6386	879.50299	22.95321	2.8481
2	12.088	BV	0.7042	3.00010e4	624.98010	97.1519

HPLC chromatogram of **4b**

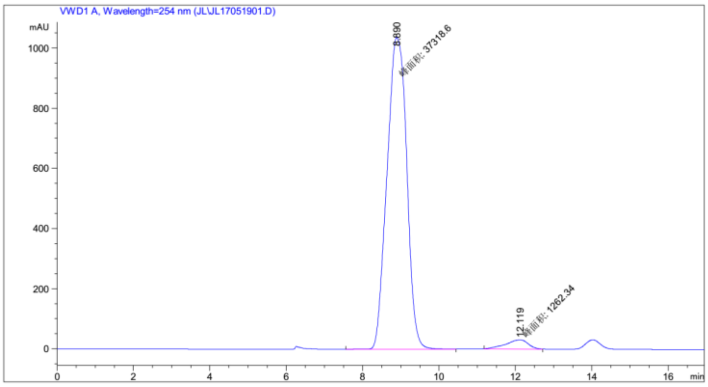

峰 #	保留时间 [min]	类型	峰宽 [min]	峰面积 [mAU*s]	峰高 [mAU]	峰面积 %
1	8.890	MM	0.5998	3.73186e4	1036.89526	96.7281
2	12.119	MM	0.6832	1262.34119	30.79416	3.2719

NMR spectra and HPLC chromatogram of **4c** and **4c′**

^{1}H and ^{13}C NMR spectra of **4c** and **4c′**

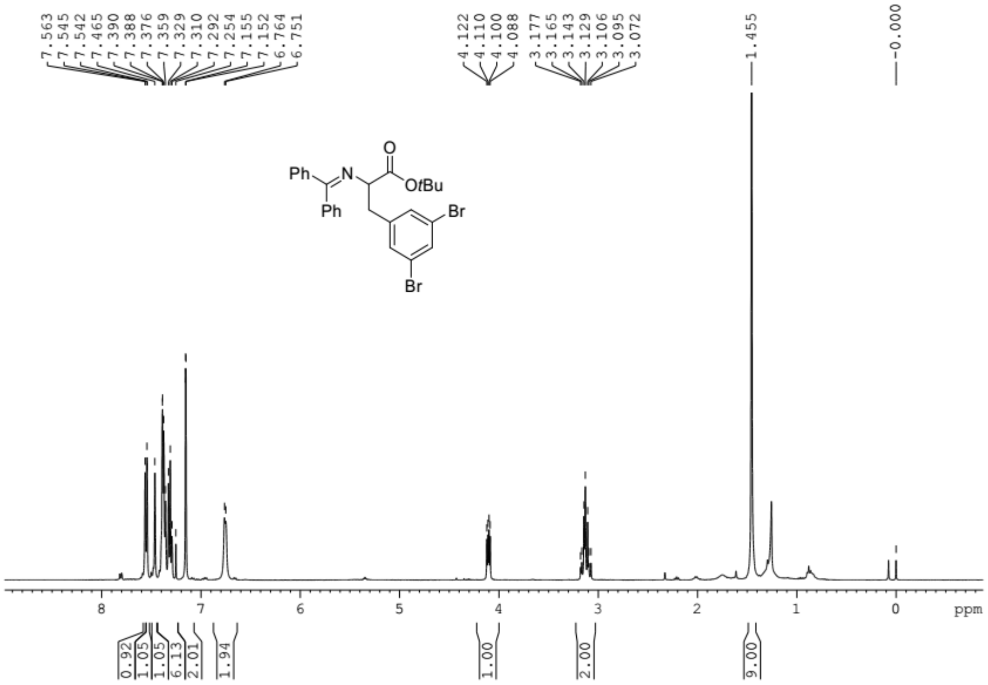

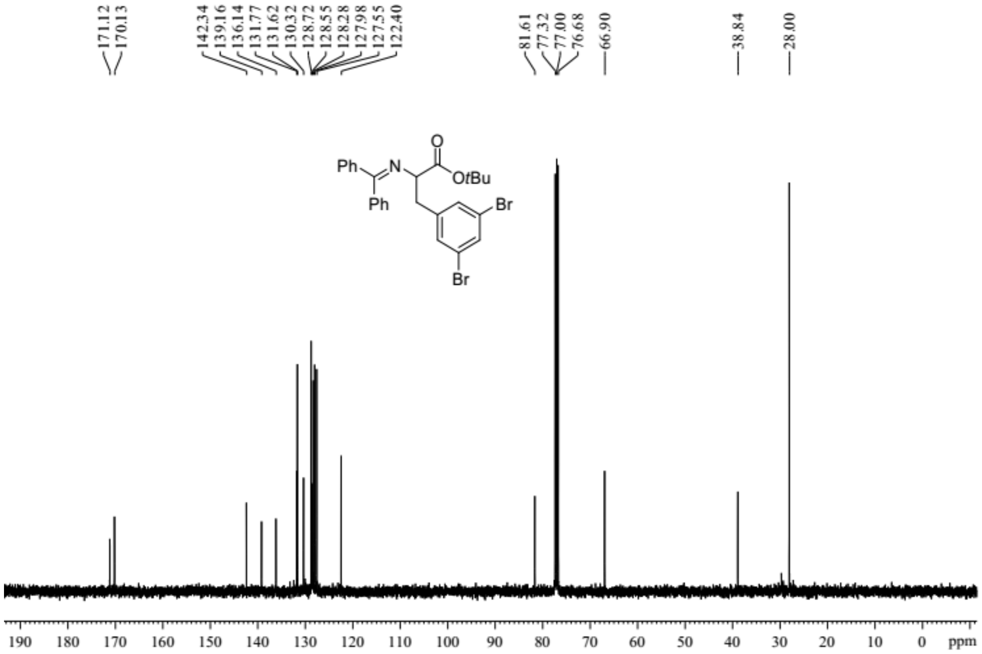

HPLC chromatogram of *rac*-**4c**

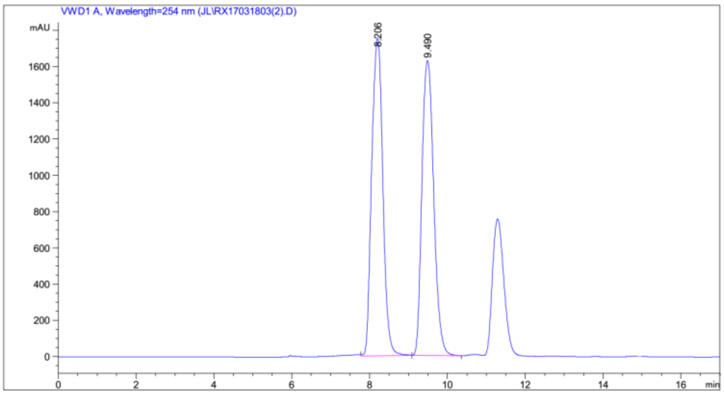

峰<br>＃	保留时间<br>[min]	类型	峰宽<br>[min]	峰面积<br>[mAU*s]	峰高<br>[mAU]	峰面积<br>%
1	8.206	VB	0.3246	3.47315e4	1751.23987	50.3585
2	9.490	BB	0.3345	3.42369e4	1625.74329	49.6415

HPLC chromatogram of **4c**

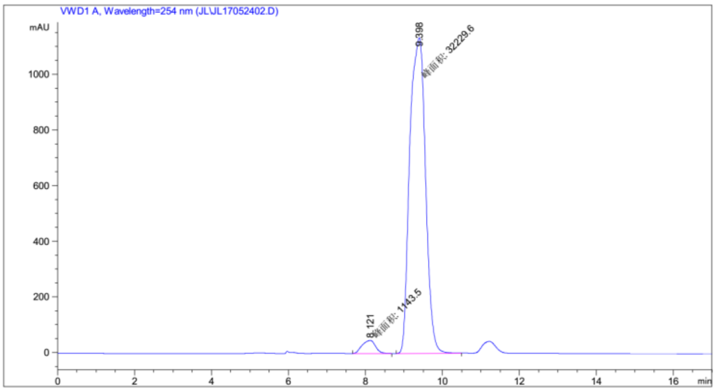

峰<br>＃	保留时间<br>[min]	类型	峰宽<br>[min]	峰面积<br>[mAU*s]	峰高<br>[mAU]	峰面积<br>%
1	8.121	MM	0.4046	1143.49902	47.10197	3.4264
2	9.398	MM	0.4735	3.22296e4	1134.37952	96.5736

HPLC chromatogram of *rac*-**4c′**

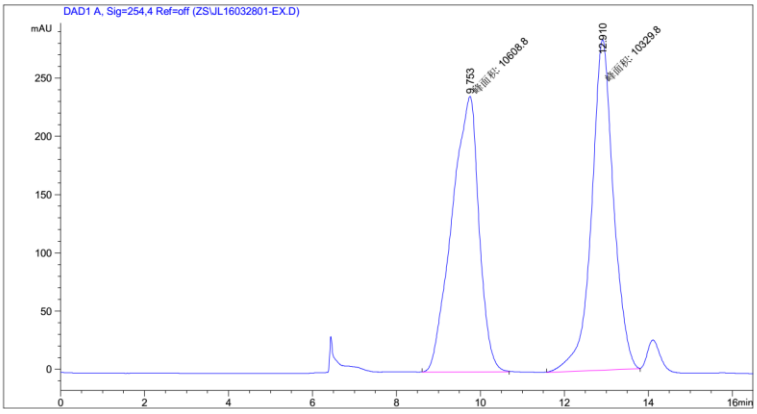

峰 #	保留时间 [min]	类型	峰宽 [min]	峰面积 [mAU*s]	峰高 [mAU]	峰面积 %
1	9.753	MM	0.7476	1.06088e4	236.51517	50.6663
2	12.910	MM	0.6068	1.03298e4	283.74463	49.3337

HPLC chromatogram of **4c′**

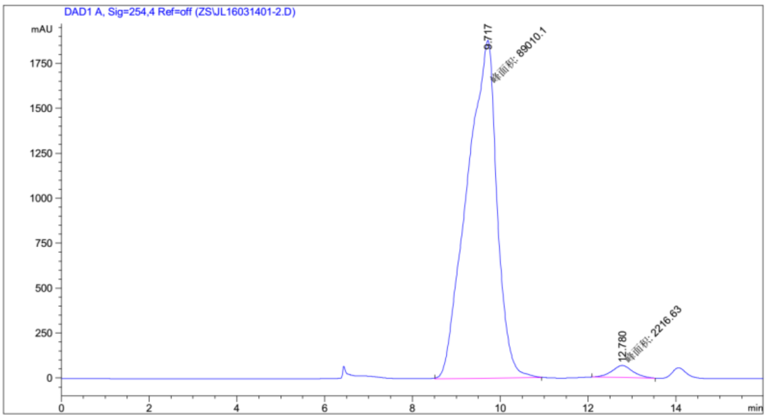

峰 #	保留时间 [min]	类型	峰宽 [min]	峰面积 [mAU*s]	峰高 [mAU]	峰面积 %
1	9.717	MM	0.7898	8.90101e4	1878.31470	97.5702
2	12.780	MM	0.5531	2216.63037	66.79173	2.4298

NMR spectra and HPLC chromatogram of **4d** and **4d′**

^{1}H and ^{13}C NMR spectra of **4d** and **4d′**

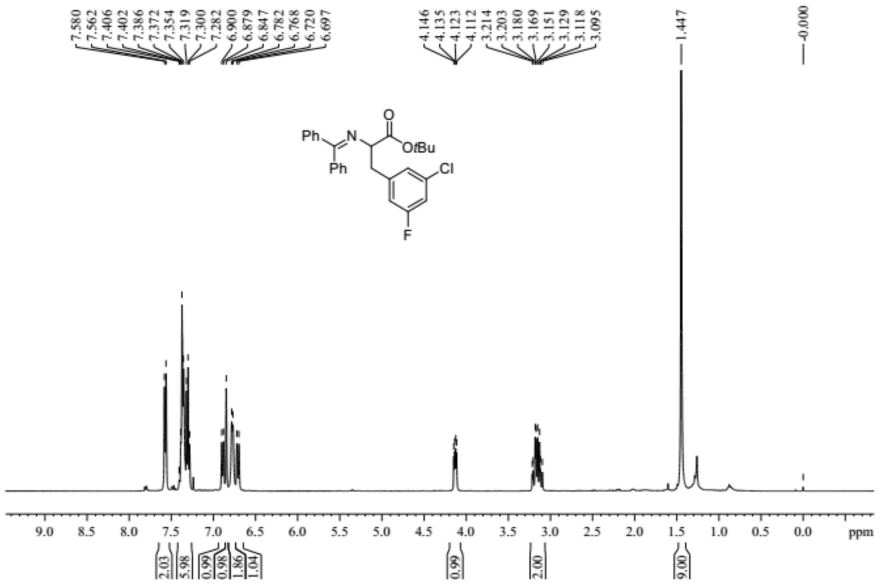

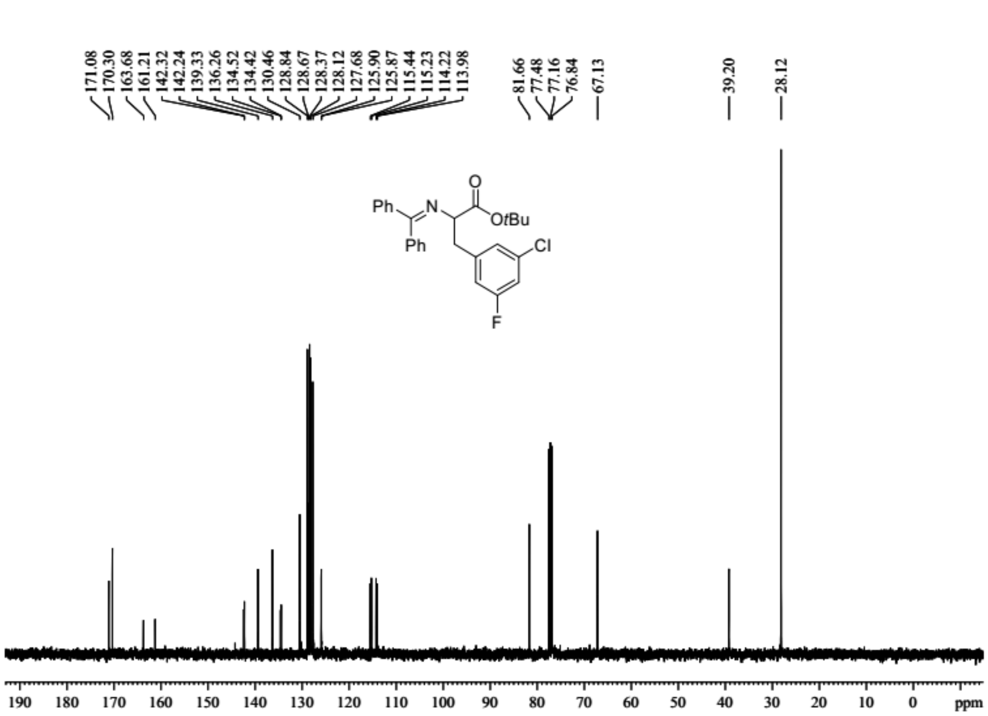

HPLC chromatogram of *rac*-**4d (4d’)**

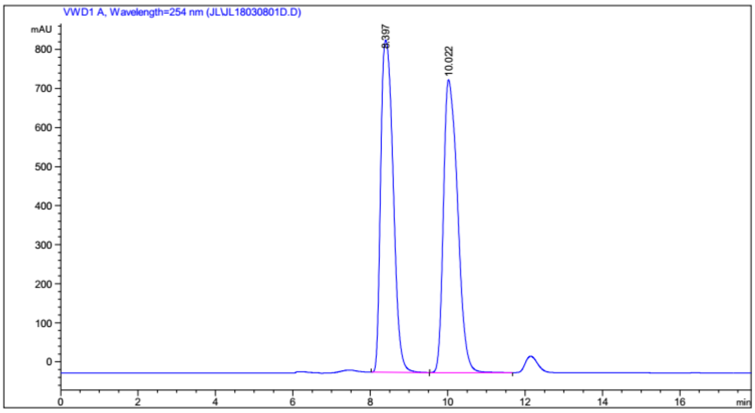

峰 #	保留时间 [min]	类型	峰宽 [min]	峰面积 [mAU*s]	峰高 [mAU]	峰面积 %
1	8.397	BB	0.3635	1.89676e4	851.13269	49.9479
2	10.022	BB	0.4092	1.90072e4	750.51227	50.0521

HPLC chromatogram of **4d**

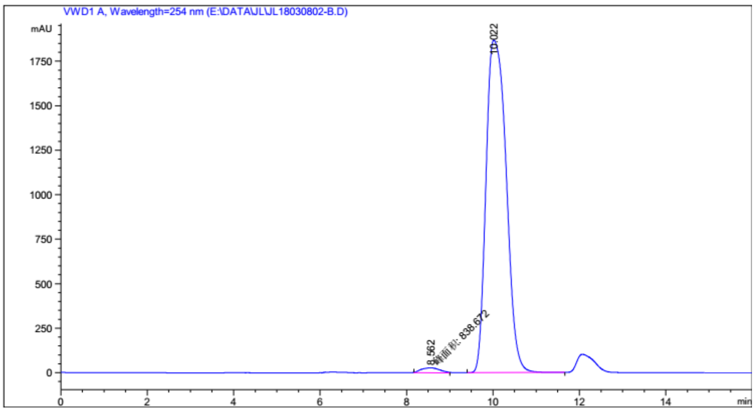

峰 #	保留时间 [min]	类型	峰宽 [min]	峰面积 [mAU*s]	峰高 [mAU]	峰面积 %
1	8.562	MM	0.5045	838.67169	27.70378	1.3681
2	10.022	BB	0.5247	6.04646e4	1868.60950	98.6319

HPLC chromatogram of **4d’**

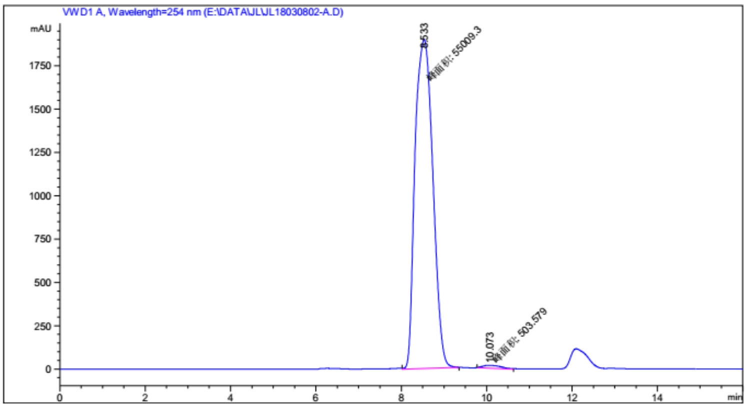

峰 #	保留时间 [min]	类型	峰宽 [min]	峰面积 [mAU*s]	峰高 [mAU]	峰面积 %
1	8.533	MM	0.4831	5.50093e4	1897.59363	99.0929
2	10.073	MM	0.5107	503.57877	16.43492	0.9071

NMR spectra and HPLC chromatogram of **4e** and **4e′**

^{1}H and ^{13}C NMR spectra of **4e** and **4e′**

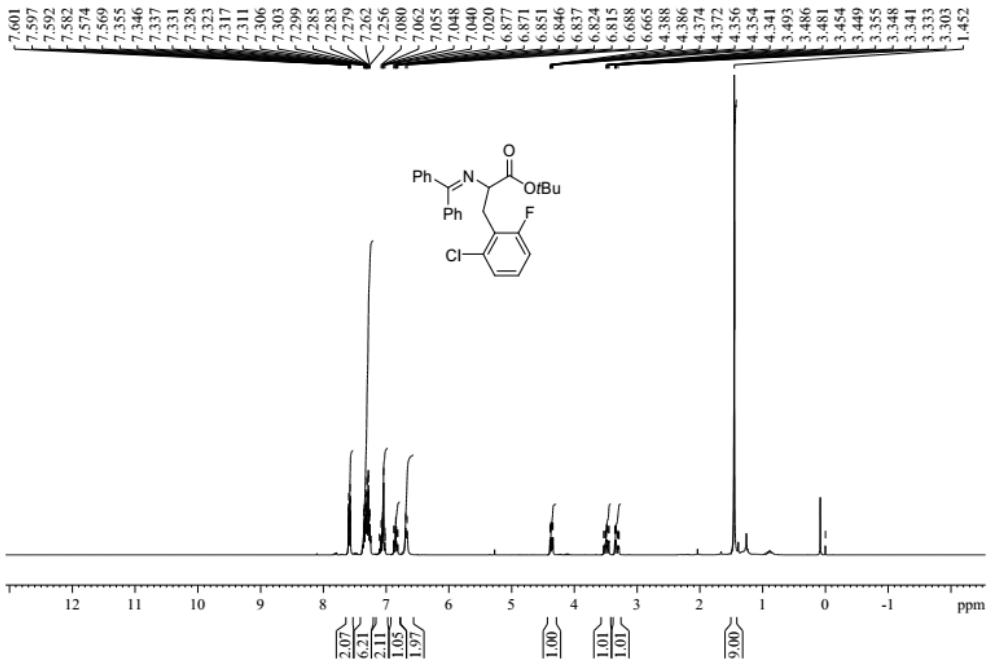

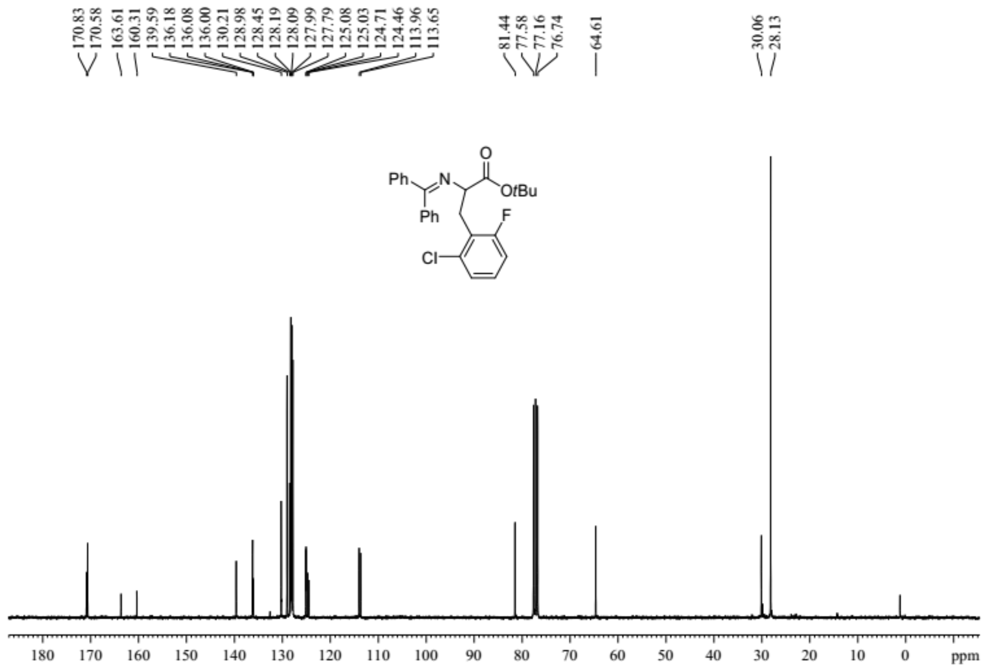

HPLC chromatogram of *rac*-**4e (4e′)**

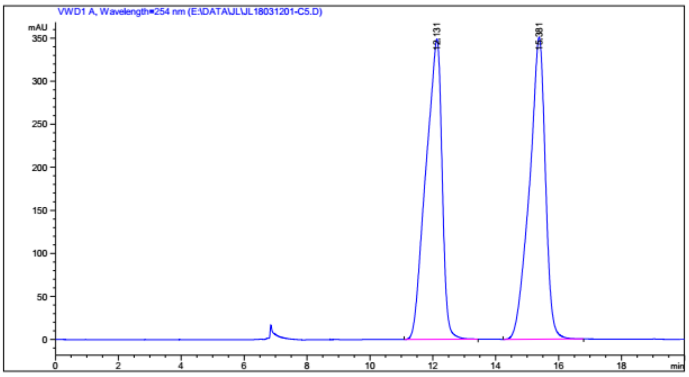

峰 #	保留时间 [min]	类型	峰宽 [min]	峰面积 [mAU*s]	峰高 [mAU]	峰面积 %
1	12.131	BB	0.4995	1.24429e4	348.58240	50.0565
2	15.381	BB	0.5121	1.24148e4	350.68979	49.9435

HPLC chromatogram of **4e**

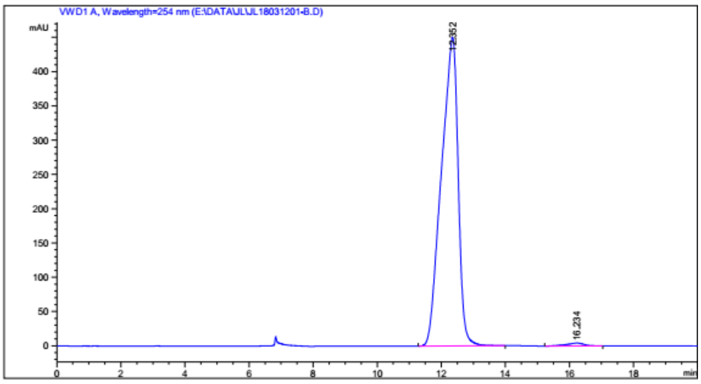

峰 #	保留时间 [min]	类型	峰宽 [min]	峰面积 [mAU*s]	峰高 [mAU]	峰面积 %
1	12.352	BB	0.5092	1.63160e4	449.82431	98.9803
2	16.234	BB	0.5348	168.08727	4.27285	1.0197

HPLC chromatogram of **4e′**

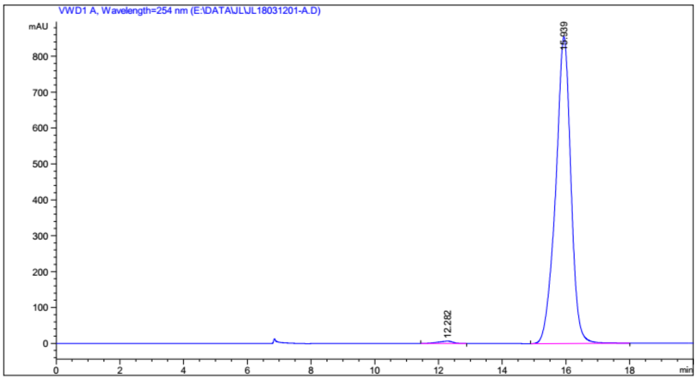

峰 #	保留时间 [min]	类型	峰宽 [min]	峰面积 [mAU*s]	峰高 [mAU]	峰面积 %
1	12.282	BB	0.4427	207.53677	6.54725	0.7250
2	15.939	BB	0.4929	2.84198e4	854.58905	99.2750

NMR spectra and HPLC chromatogram of **4f** and **4f′**

^{1}H and ^{13}C NMR spectra of **4f** and **4f′**

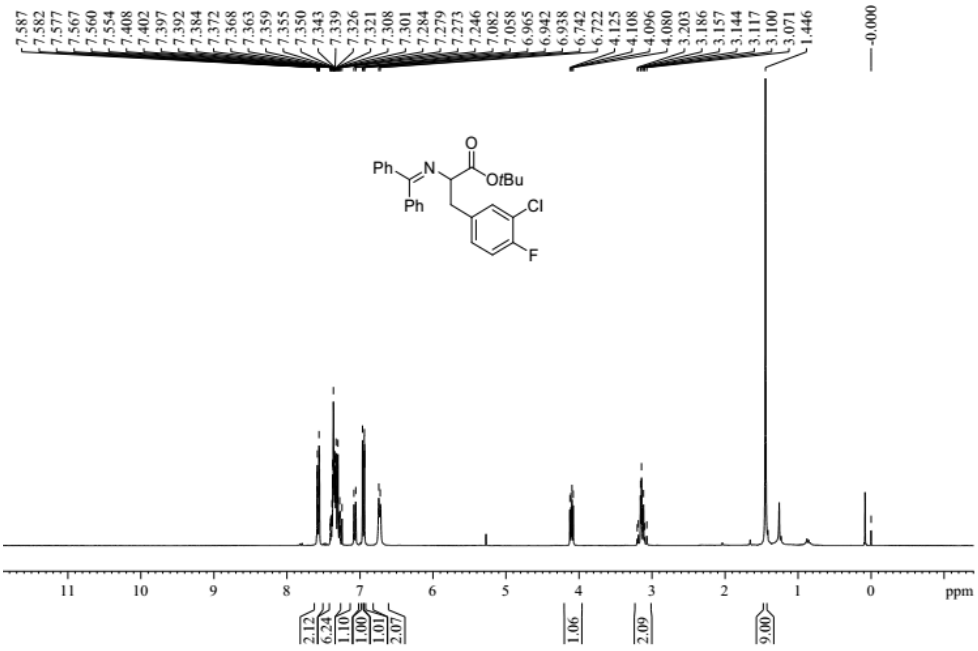

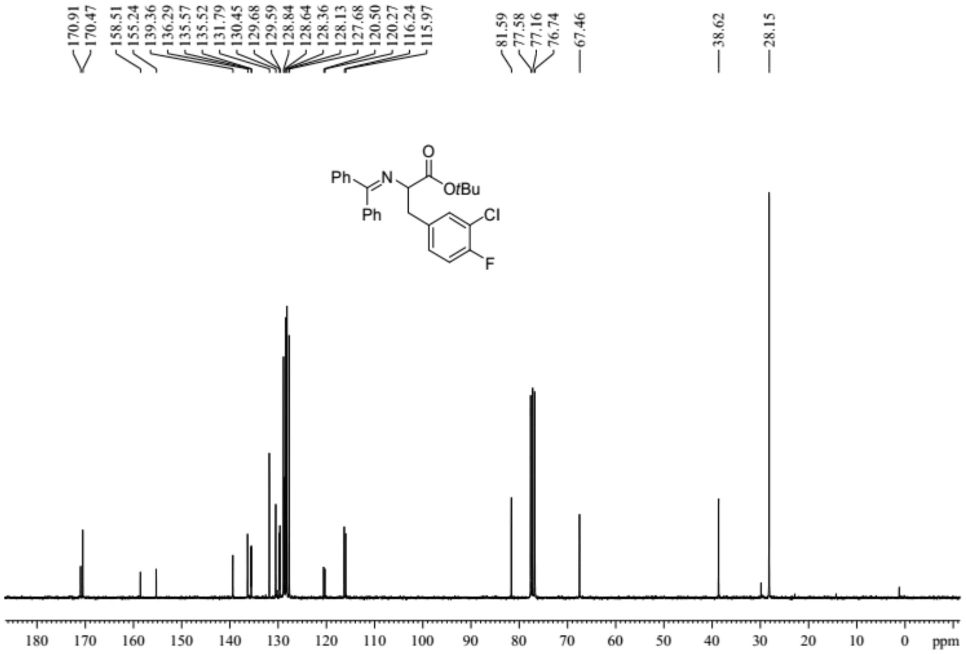

HPLC chromatogram of *rac*-**4f (4f′)**

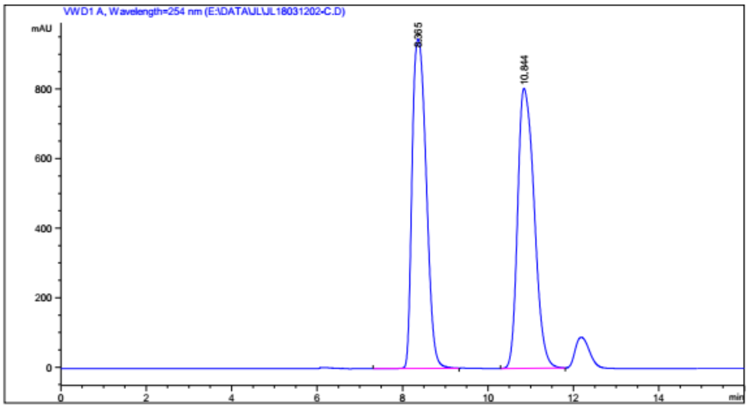

峰 #	保留时间 [min]	类型	峰宽 [min]	峰面积 [mAU*s]	峰高 [mAU]	峰面积 %
1	8.365	BB	0.3713	2.16139e4	946.90161	49.9357
2	10.844	BB	0.4321	2.16695e4	805.00104	50.0643

HPLC chromatogram of **4f**

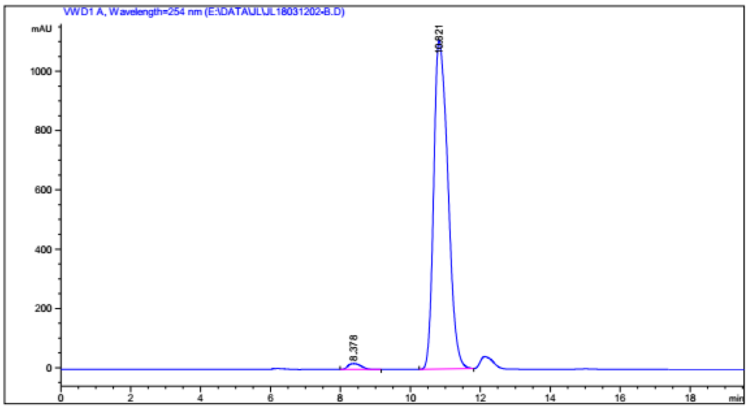

峰 #	保留时间 [min]	类型	峰宽 [min]	峰面积 [mAU*s]	峰高 [mAU]	峰面积 %
1	8.378	BB	0.4216	495.67624	19.51971	1.5592
2	10.821	BB	0.4572	3.12955e4	1109.90698	98.4408

HPLC chromatogram of **4f′**

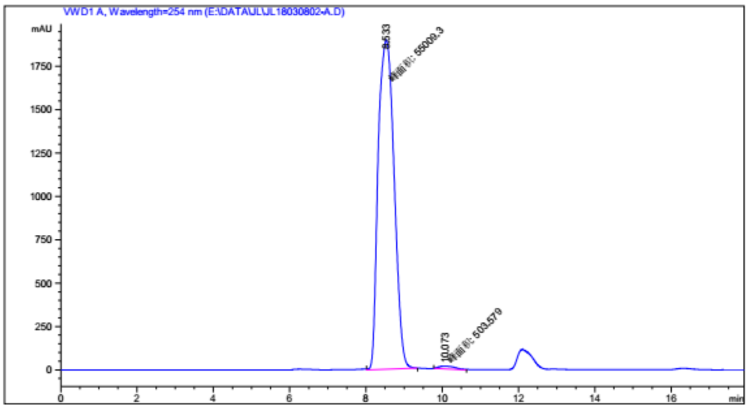

峰 #	保留时间 [min]	类型	峰宽 [min]	峰面积 [mAU*s]	峰高 [mAU]	峰面积 %
1	8.533	MM	0.4831	5.50093e4	1897.59363	99.0929
2	10.073	MM	0.5107	503.57877	16.43492	0.9071

NMR spectra and HPLC chromatogram of **4g** and **4g′**

^{1}H and ^{13}C NMR spectra of **4g** and **4g′**

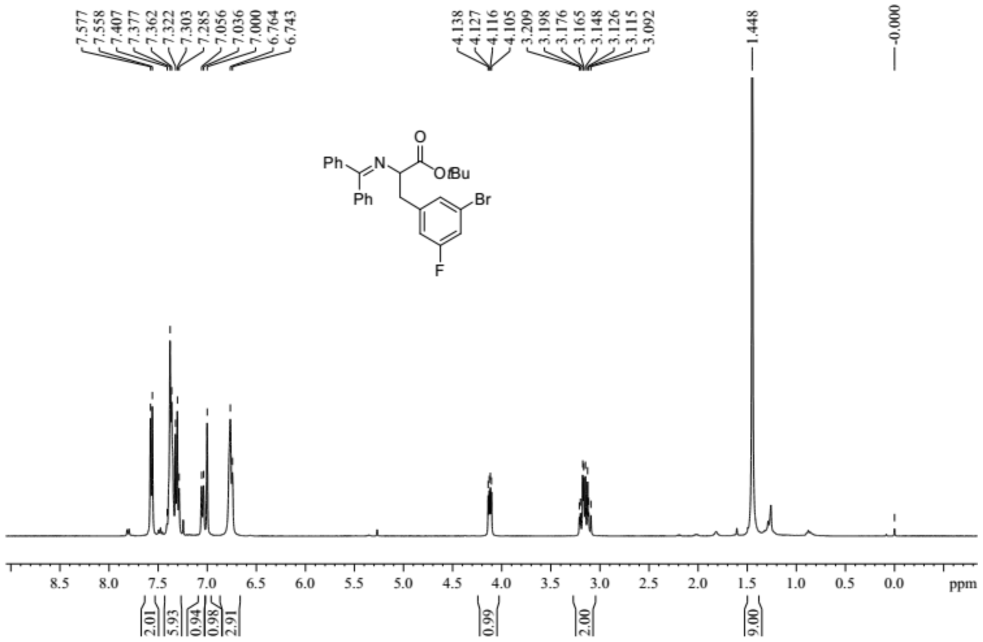

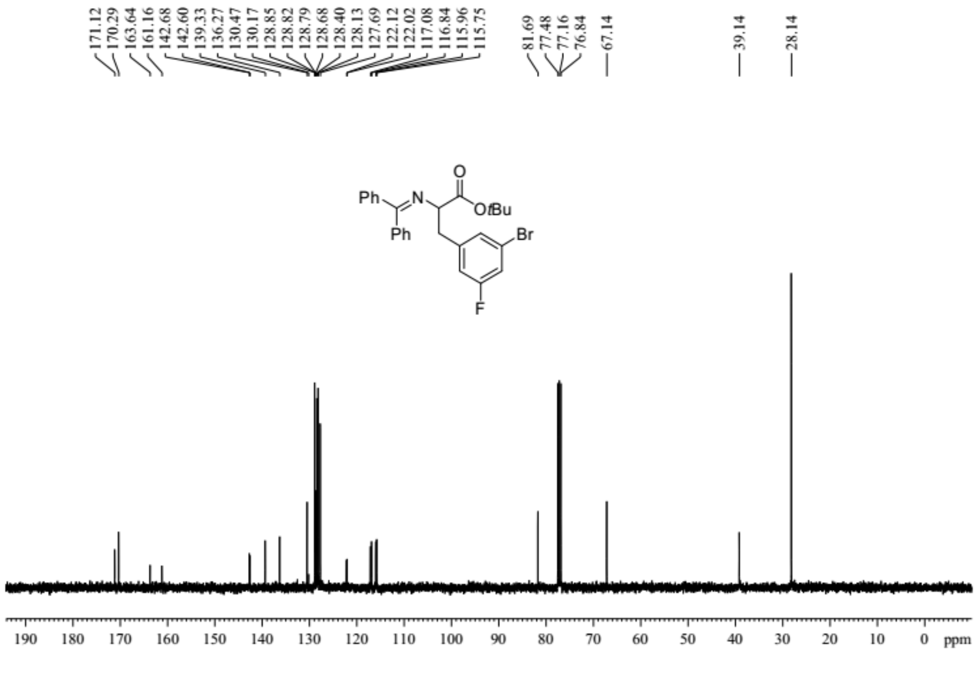

HPLC chromatogram of *rac*-**4g (4g′)**

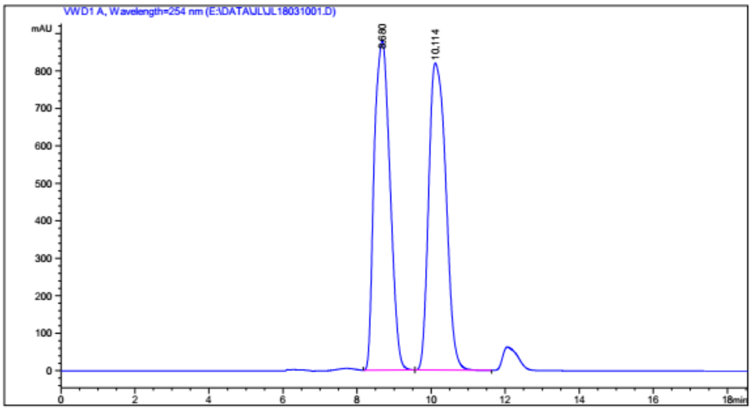

峰 #	保留时间 [min]	类型	峰宽 [min]	峰面积 [mAU*s]	峰高 [mAU]	峰面积 %
1	8.680	BB	0.4854	2.59925e4	881.44440	49.6593
2	10.114	BB	0.5238	2.63492e4	819.41278	50.3407

HPLC chromatogram of **4g**

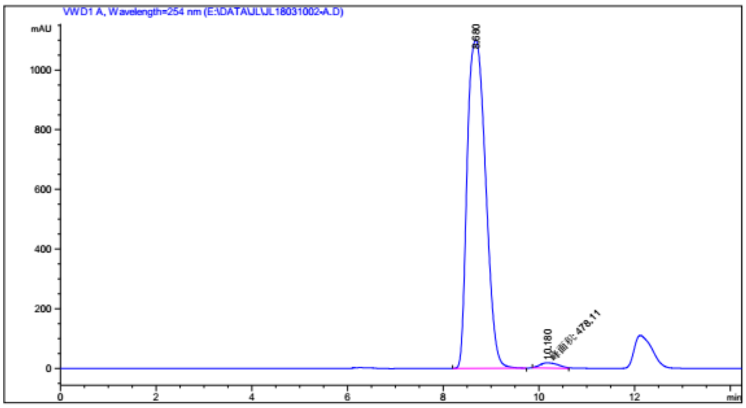

峰 #	保留时间 [min]	类型	峰宽 [min]	峰面积 [mAU*s]	峰高 [mAU]	峰面积 %
1	8.680	BB	0.4327	2.92482e4	1099.68555	98.3916
2	10.180	MM	0.4507	478.10956	17.67984	1.6084

HPLC chromatogram of **4g′**

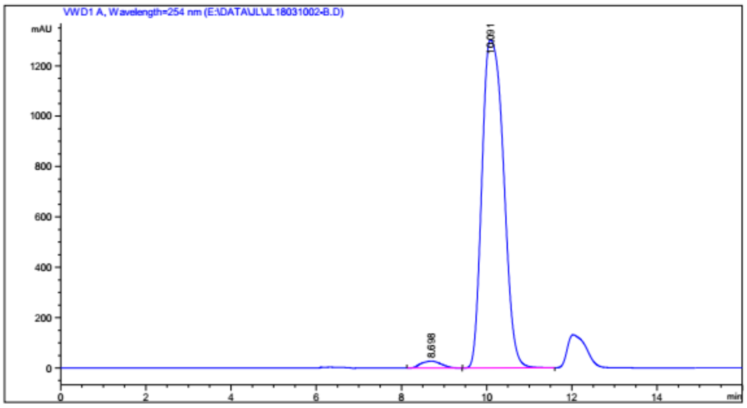

峰 #	保留时间 [min]	类型	峰宽 [min]	峰面积 [mAU*s]	峰高 [mAU]	峰面积 %
1	8.698	BB	0.4743	898.62427	27.62494	1.9271
2	10.091	BB	0.5696	4.57313e4	1303.56702	98.0729

NMR spectra and HPLC chromatogram of **4h** and **4h′**

^{1}H and ^{13}C NMR spectra of **4h** and **4h′**

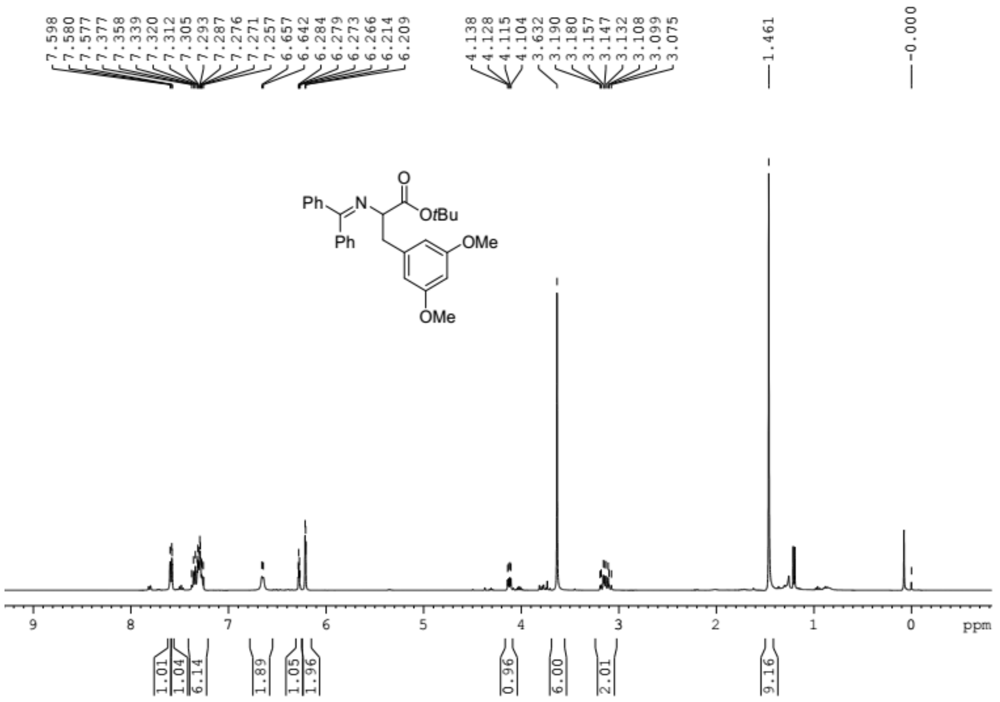

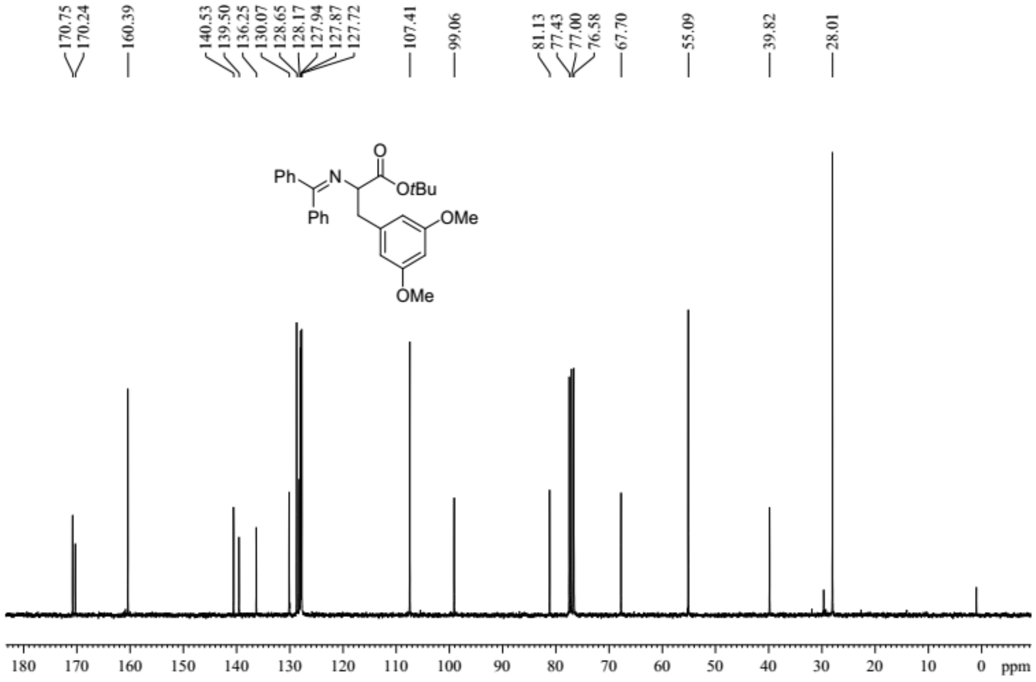

HPLC chromatogram of *rac*-**4h (4h’)**

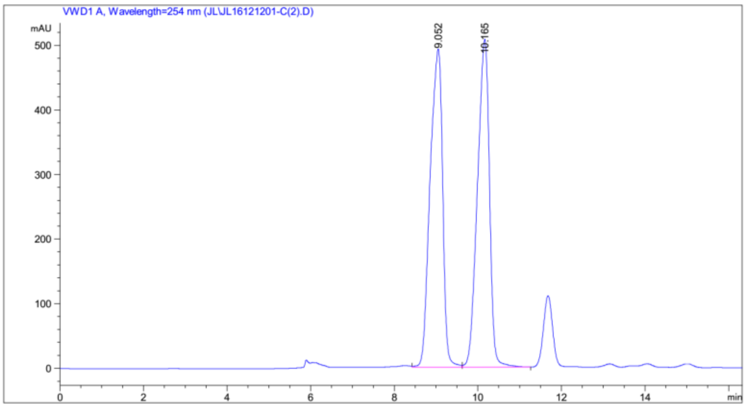

峰 #	保留时间 [min]	类型	峰宽 [min]	峰面积 [mAU*s]	峰高 [mAU]	峰面积 %
1	9.052	VV	0.3466	1.02141e4	492.87582	50.2016
2	10.165	VB	0.3188	1.01321e4	507.64380	49.7984

HPLC chromatogram of **4h**

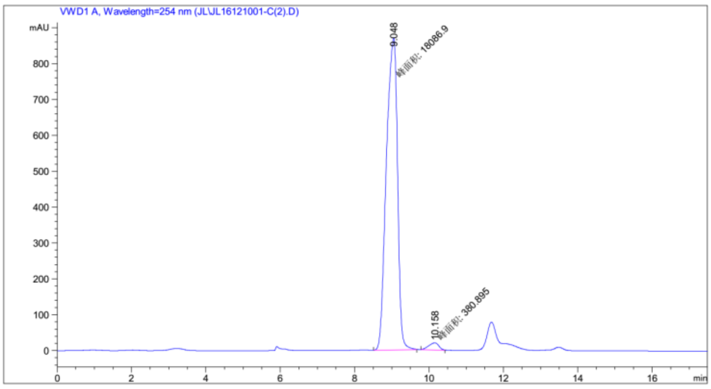

峰 #	保留时间 [min]	类型	峰宽 [min]	峰面积 [mAU*s]	峰高 [mAU]	峰面积 %
1	9.048	MM	0.3465	1.80869e4	869.90308	97.9375
2	10.158	MM	0.3171	380.89493	20.02122	2.0625

HPLC chromatogram of **4h’**

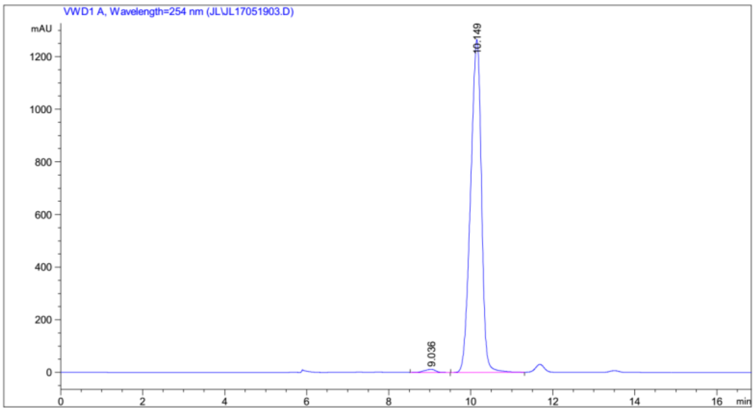

峰 #	保留时间 [min]	类型	峰宽 [min]	峰面积 [mAU*s]	峰高 [mAU]	峰面积 %
1	9.036	BB	0.3164	224.44653	11.65545	0.9551
2	10.149	BV	0.2889	2.32746e4	1264.93433	99.0449

NMR spectra and HPLC chromatogram of **4i** and **4i'**

^{1}H and ^{13}C NMR spectra of **4i** and **4i'**

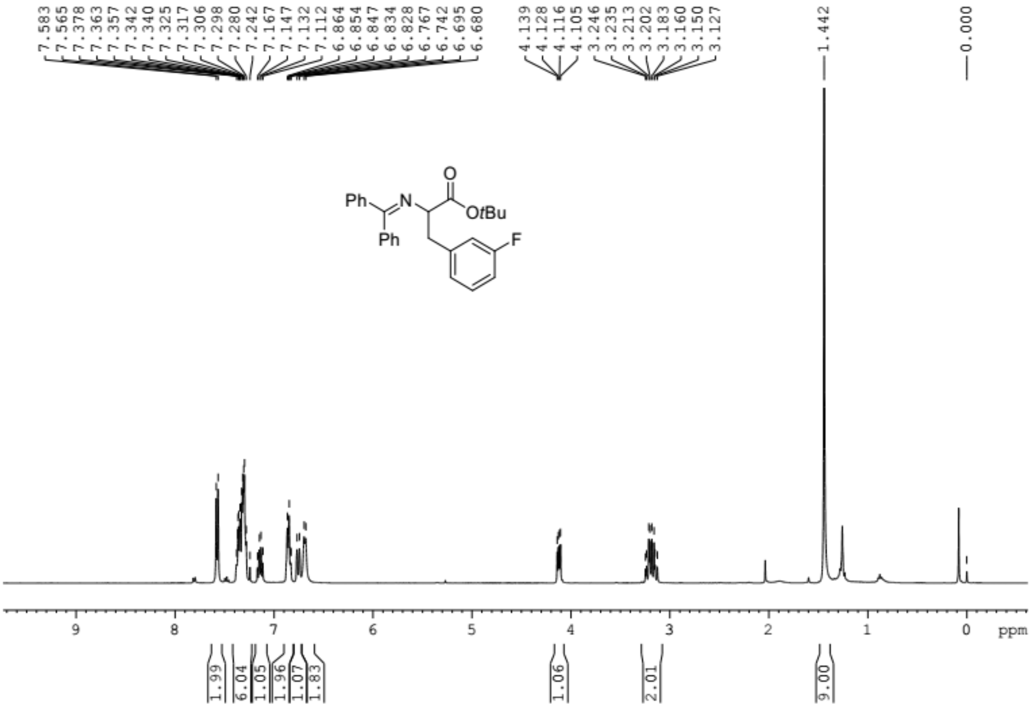

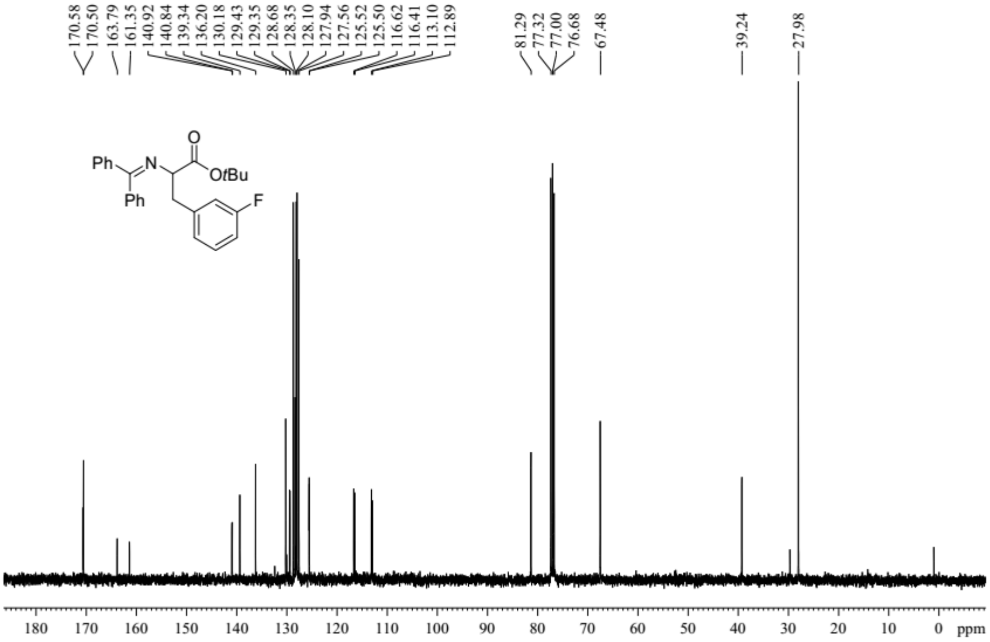

HPLC chromatogram of *rac*-**4i (4i’)**

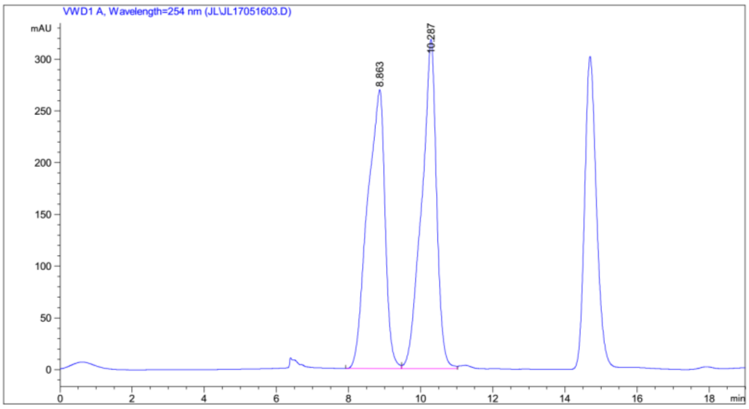

峰 #	保留时间 [min]	类型	峰宽 [min]	峰面积 [mAU*s]	峰高 [mAU]	峰面积 %
1	8.863	BV	0.4611	9102.69434	269.42279	49.9121
2	10.287	VV	0.4004	9134.73828	318.25516	50.0879

HPLC chromatogram of **4i**

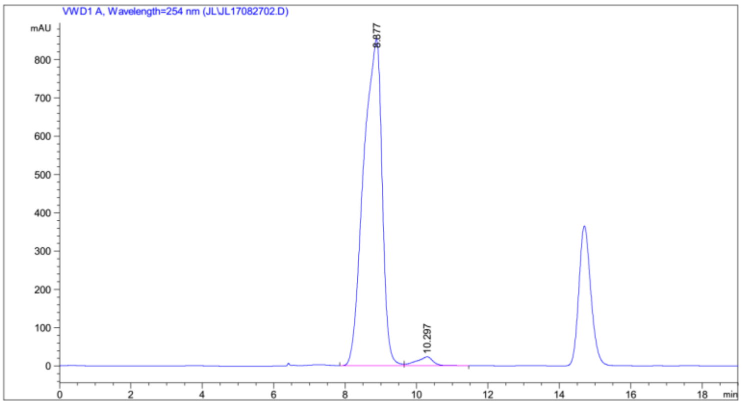

峰 #	保留时间 [min]	类型	峰宽 [min]	峰面积 [mAU*s]	峰高 [mAU]	峰面积 %
1	8.877	BV	0.4844	3.07185e4	852.67920	97.6025
2	10.297	VV	0.4454	754.58209	23.35627	2.3975

HPLC chromatogram of **4i’**

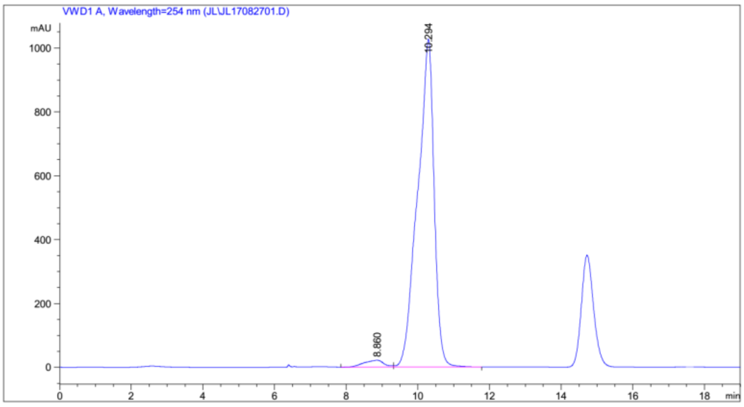

峰 #	保留时间 [min]	类型	峰宽 [min]	峰面积 [mAU*s]	峰高 [mAU]	峰面积 %
1	8.860	BV	0.5257	838.35797	21.73728	2.4940
2	10.294	VB	0.4394	3.27762e4	1026.64282	97.5060

NMR spectra and HPLC chromatogram of **4j** and **4j′**

^{1}H and ^{13}C NMR spectra of **4j** and **4j′**

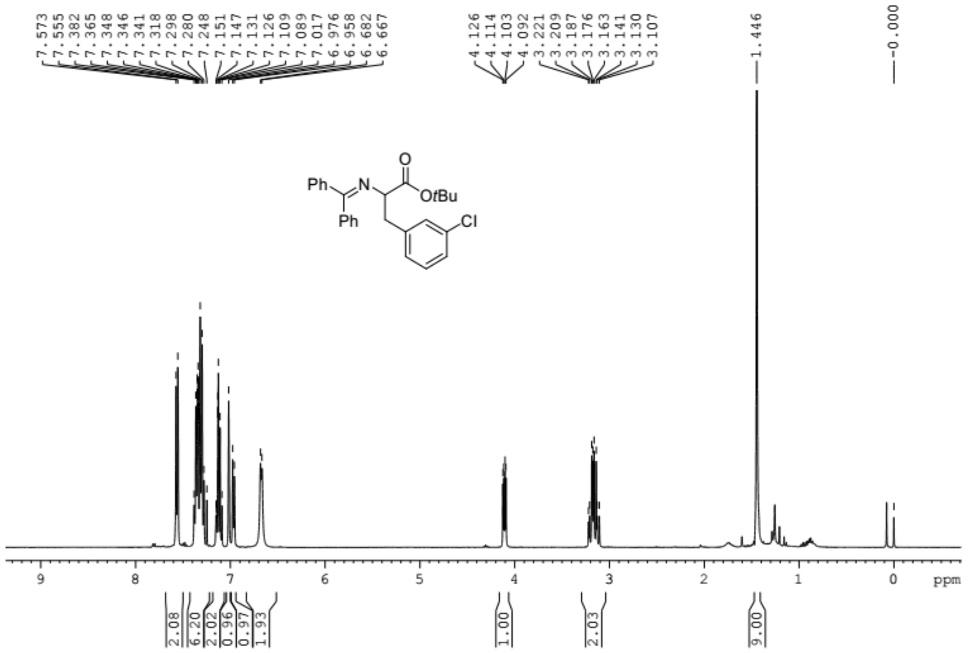

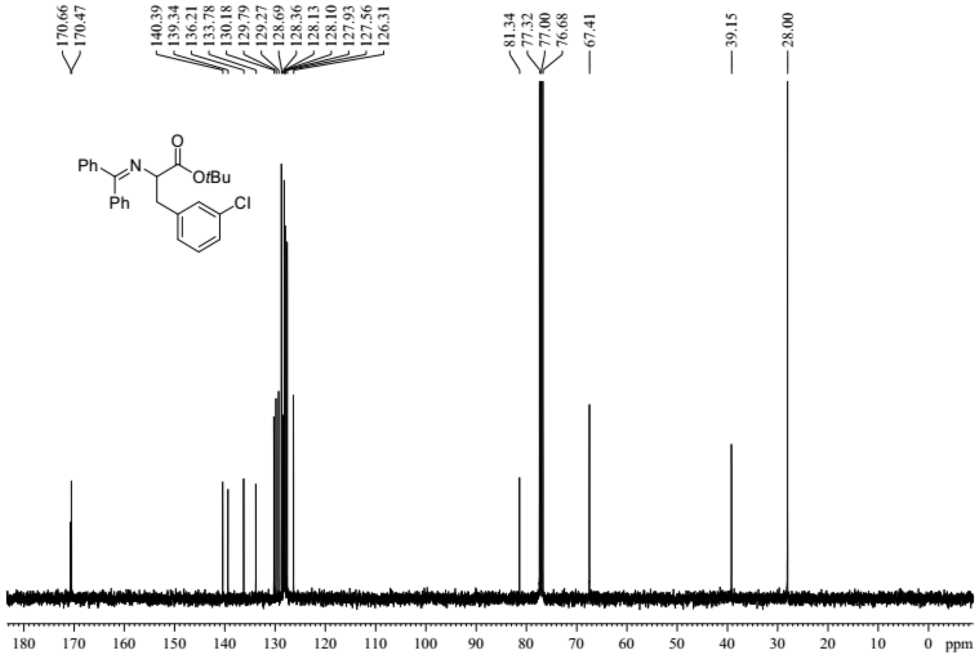

HPLC chromatogram of *rac*-**4j**

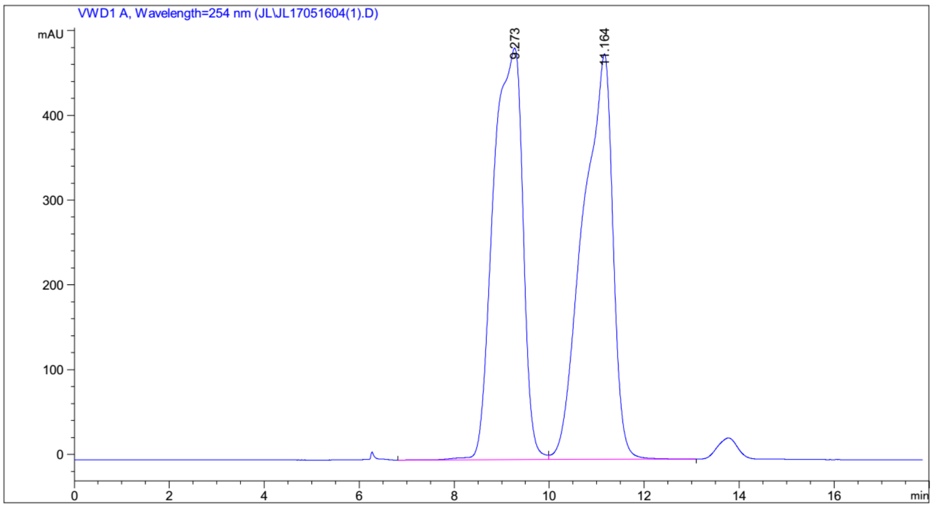

峰 #	保留时间 [min]	类型	峰宽 [min]	峰面积 [mAU*s]	峰高 [mAU]	峰面积 %
1	9.273	BV	0.5766	2.09055e4	485.47672	49.9838
2	11.164	VB	0.5873	2.09190e4	478.37338	50.0162

HPLC chromatogram of **4j**

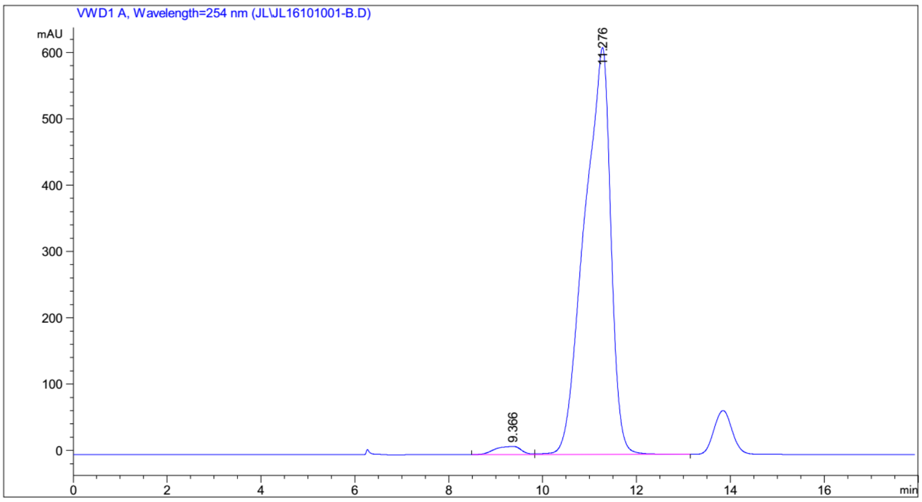

峰 #	保留时间 [min]	类型	峰宽 [min]	峰面积 [mAU*s]	峰高 [mAU]	峰面积 %
1	9.366	BV	0.5233	467.99289	12.23717	1.9181
2	11.276	VB	0.5309	2.39302e4	613.37714	98.0819

HPLC chromatogram of *rac*-**4j'**

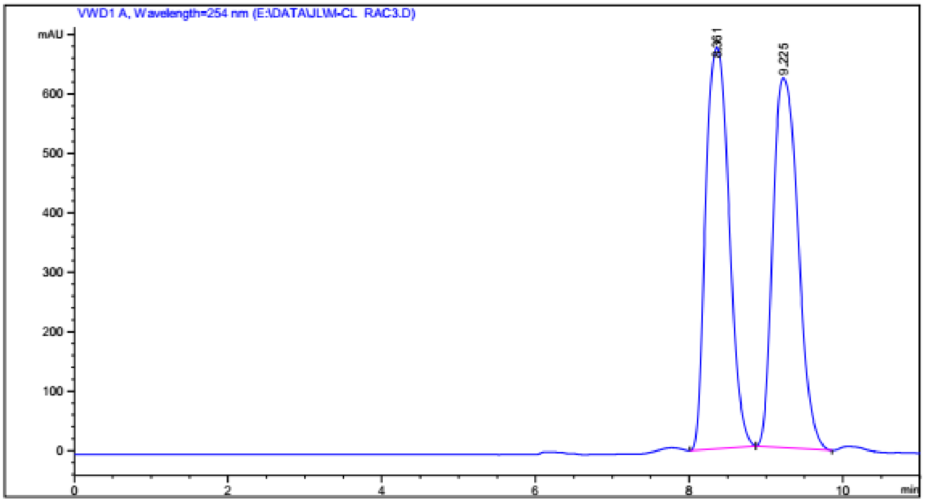

峰 #	保留时间 [min]	类型	峰宽 [min]	峰面积 [mAU*s]	峰高 [mAU]	峰面积 %
1	8.361	BB	0.3448	1.42203e4	675.20050	50.1295
2	9.225	BB	0.3661	1.41469e4	621.65082	49.8705

HPLC chromatogram of **4j'**

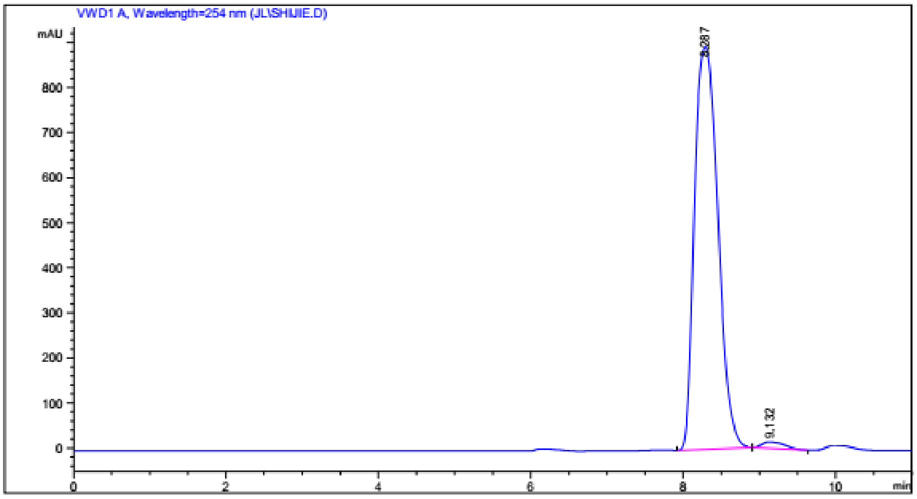

峰 #	保留时间 [min]	类型	峰宽 [min]	峰面积 [mAU*s]	峰高 [mAU]	峰面积 %
1	8.287	BB	0.3497	1.90647e4	892.75867	98.4652
2	9.132	BB	0.3496	297.15601	14.16903	1.5348

NMR spectra and HPLC chromatogram of **4k** and **4k'**

^{1}H and ^{13}C NMR spectra of **4k** and **4k'**

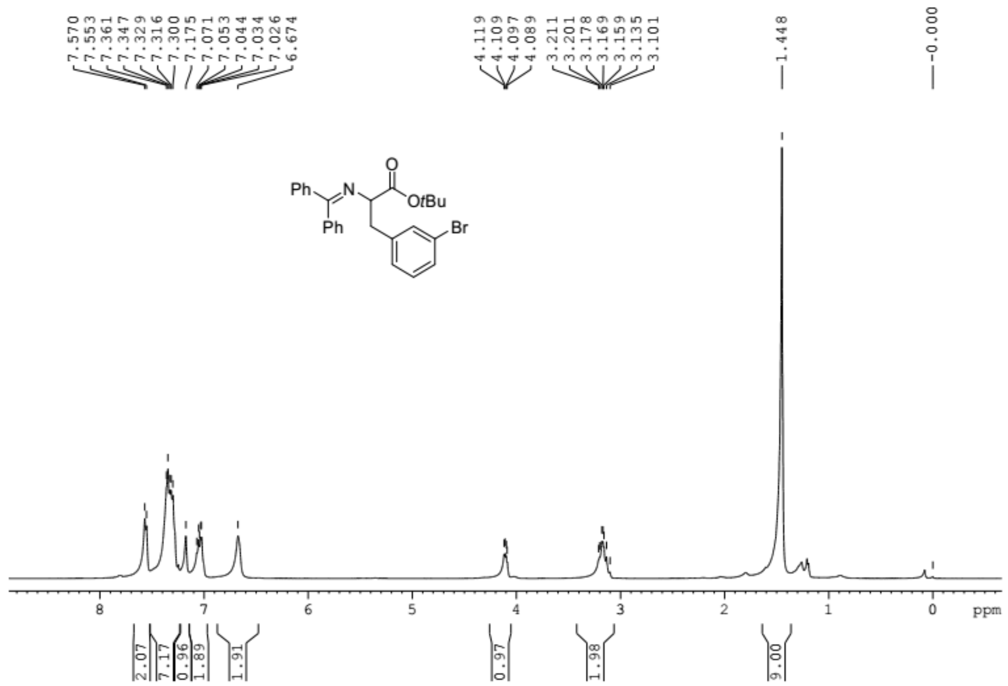

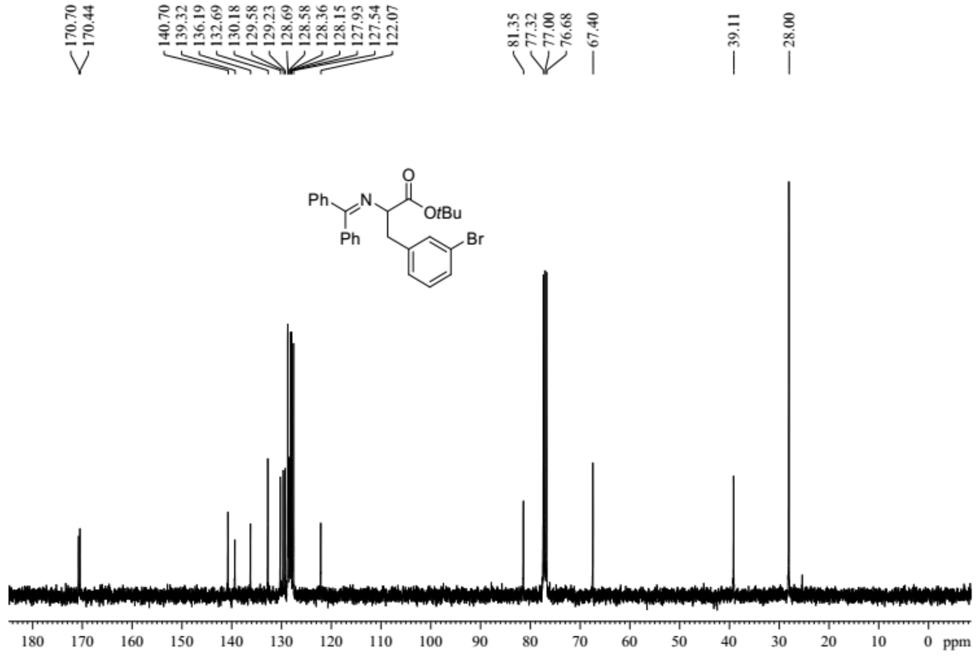

HPLC chromatogram of *rac*-**4k (4k')**

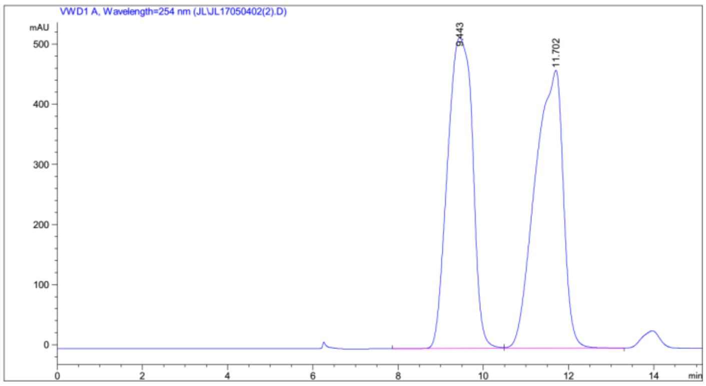

峰 #	保留时间 [min]	类型	峰宽 [min]	峰面积 [mAU*s]	峰高 [mAU]	峰面积 %
1	9.443	BV	0.6759	2.11636e4	516.67102	49.9899
2	11.702	VB	0.6028	2.11721e4	462.24307	50.0101

HPLC chromatogram of **4k**

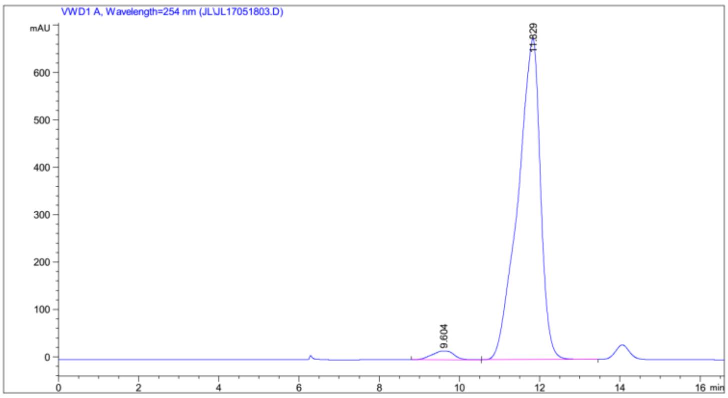

峰 #	保留时间 [min]	类型	峰宽 [min]	峰面积 [mAU*s]	峰高 [mAU]	峰面积 %
1	9.604	BB	0.5685	689.75122	18.45819	2.5488
2	11.829	BB	0.5854	2.63718e4	677.08789	97.4512

HPLC chromatogram of **4k'**

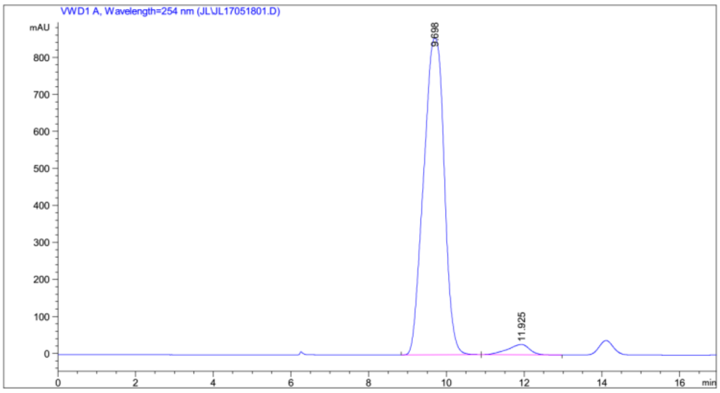

峰 #	保留时间 [min]	类型	峰宽 [min]	峰面积 [mAU*s]	峰高 [mAU]	峰面积 %
1	9.698	BB	0.5579	3.14106e4	855.63013	96.6341
2	11.925	BB	0.5824	1094.09119	27.83064	3.3659

NMR spectra and HPLC chromatogram of **4l** and **4l′**

^{1}H and ^{13}C NMR spectra of **4l** and **4l′**

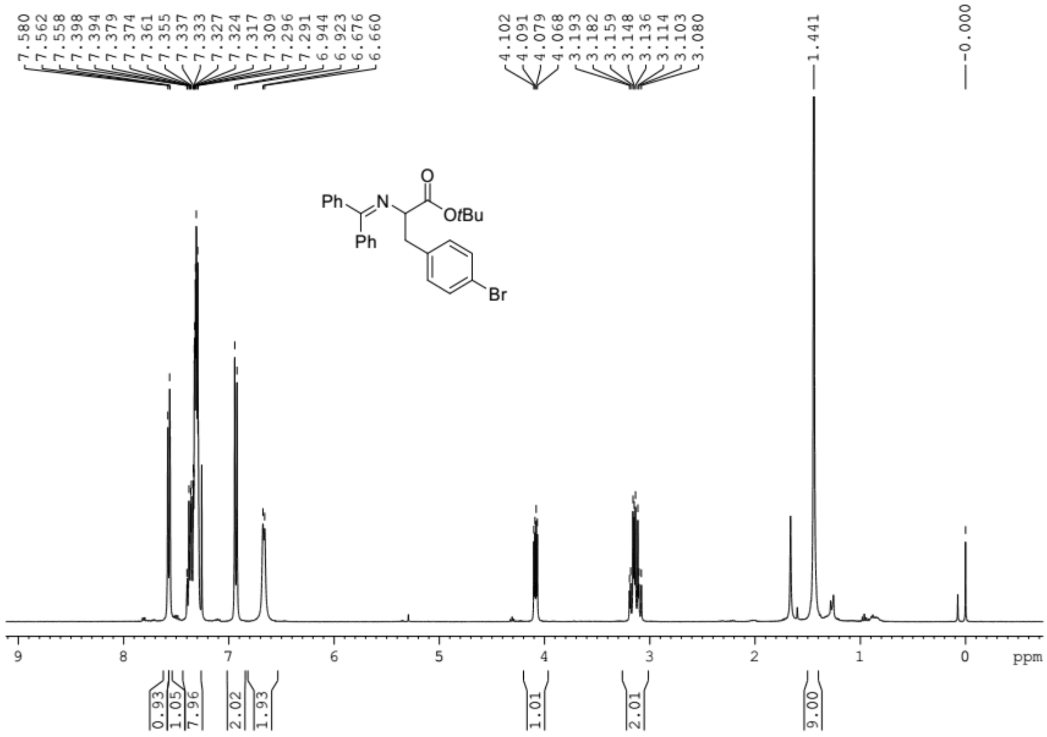

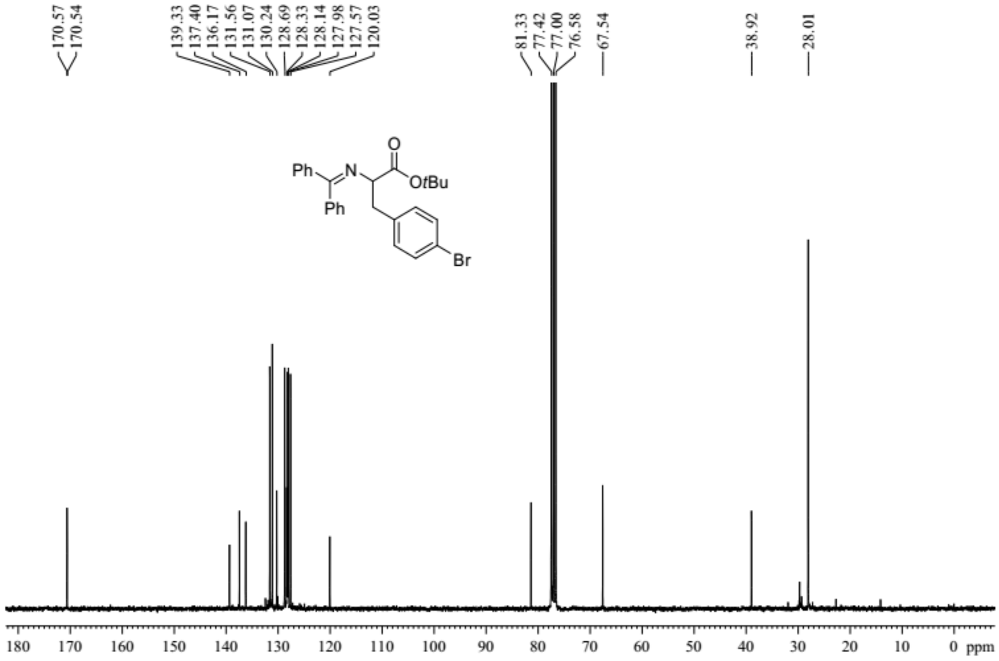

HPLC chromatogram of *rac*-**4l (4l')**

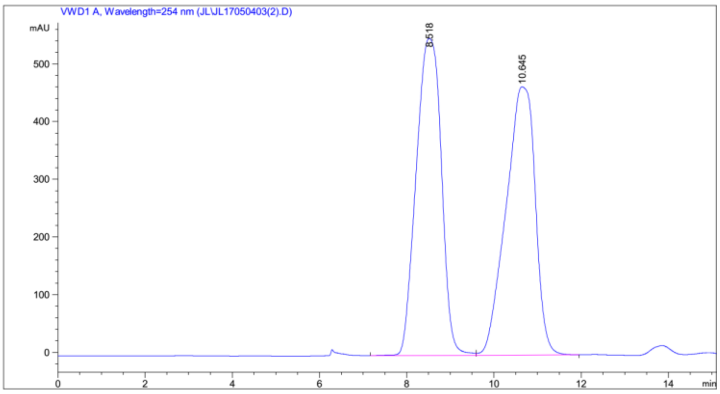

峰 #	保留时间 [min]	类型	峰宽 [min]	峰面积 [mAU*s]	峰高 [mAU]	峰面积 %
1	8.518	BV	0.6448	2.20682e4	549.42438	50.3824
2	10.645	VB	0.6936	2.17332e4	465.13022	49.6176

HPLC chromatogram of **4l**

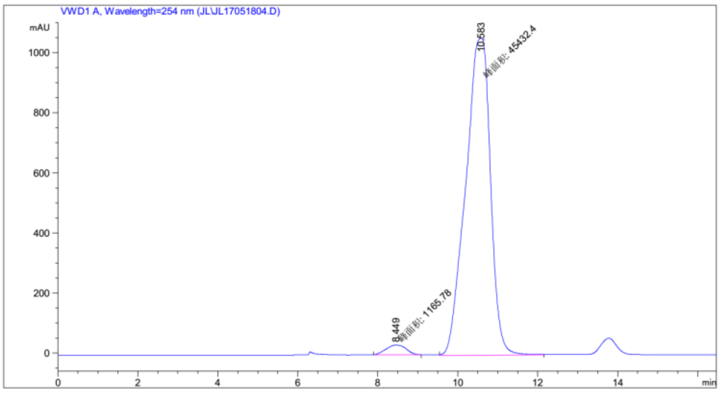

峰 #	保留时间 [min]	类型	峰宽 [min]	峰面积 [mAU*s]	峰高 [mAU]	峰面积 %
1	8.449	MM	0.5908	1165.78491	32.88894	2.5018
2	10.583	MM	0.7173	4.54324e4	1055.66943	97.4982

HPLC chromatogram of **4l'**

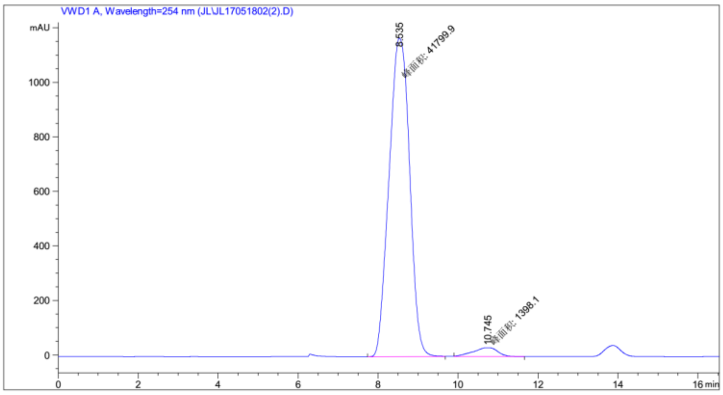

峰 #	保留时间 [min]	类型	峰宽 [min]	峰面积 [mAU*s]	峰高 [mAU]	峰面积 %
1	8.535	MM	0.5960	4.17999e4	1168.88110	96.7635
2	10.745	MM	0.7238	1398.09924	32.19222	3.2365

NMR spectra and HPLC chromatogram of **4m** and **4m'**

^{1}H and ^{13}C NMR spectra of **4m** and **4m'**

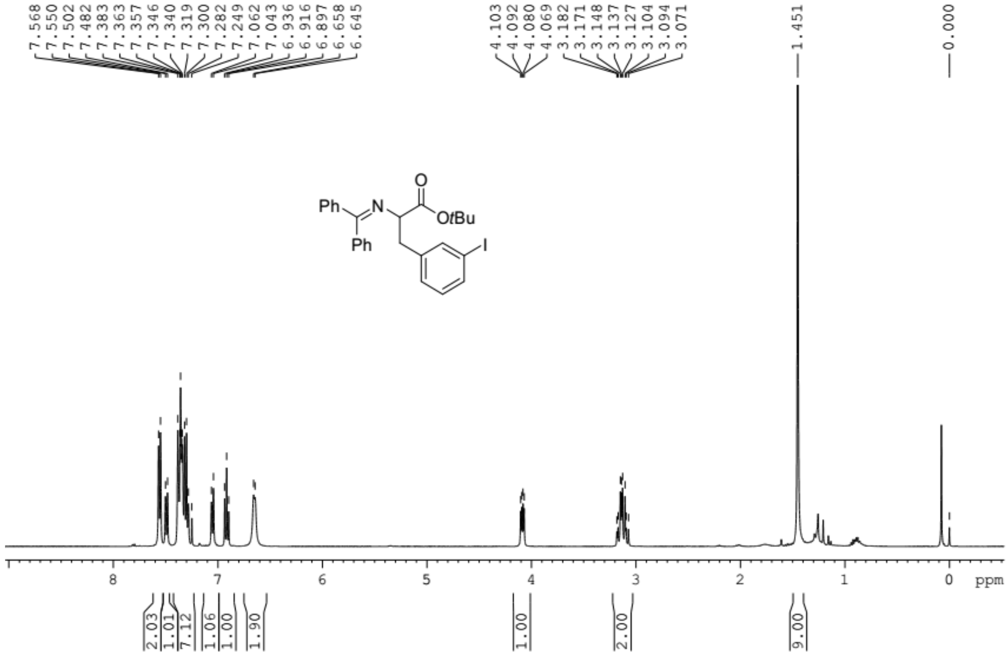

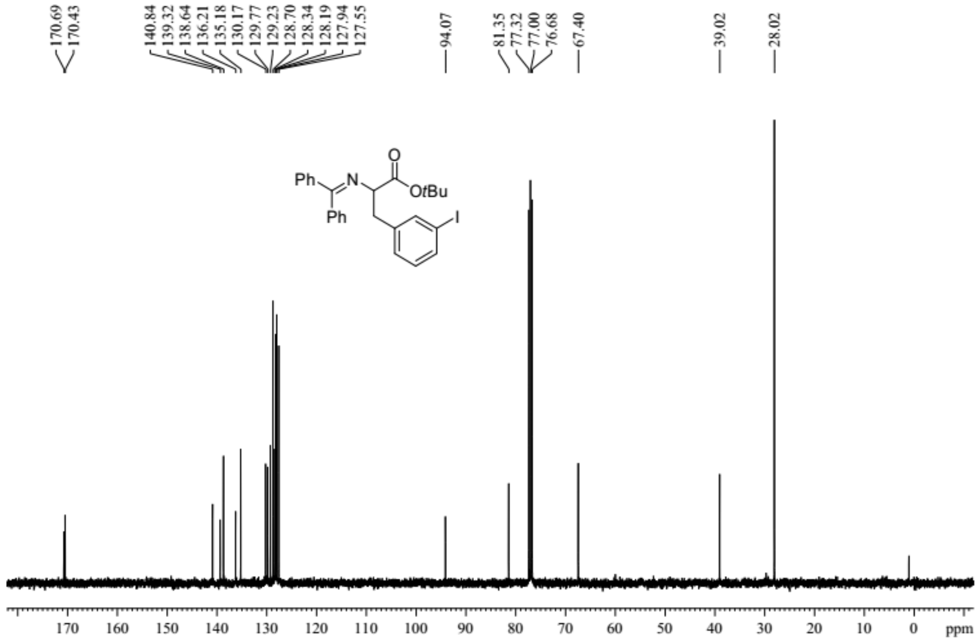

HPLC chromatogram of *rac*-**4m (4m′)**

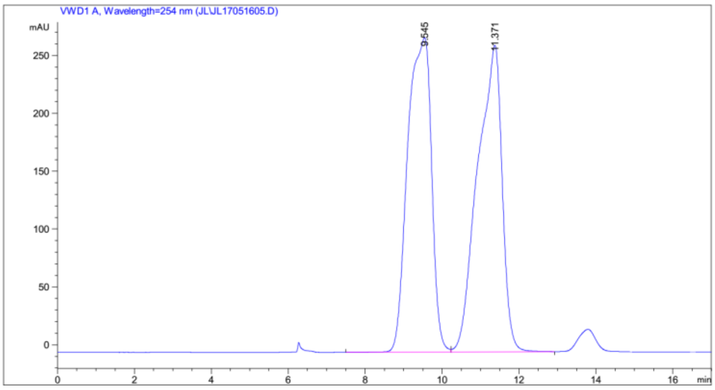

峰 #	保留时间 [min]	类型	峰宽 [min]	峰面积 [mAU*s]	峰高 [mAU]	峰面积 %
1	9.545	BV	0.5767	1.17008e4	271.68500	49.9704
2	11.371	VB	0.5974	1.17146e4	264.91830	50.0296

HPLC chromatogram of **4m**

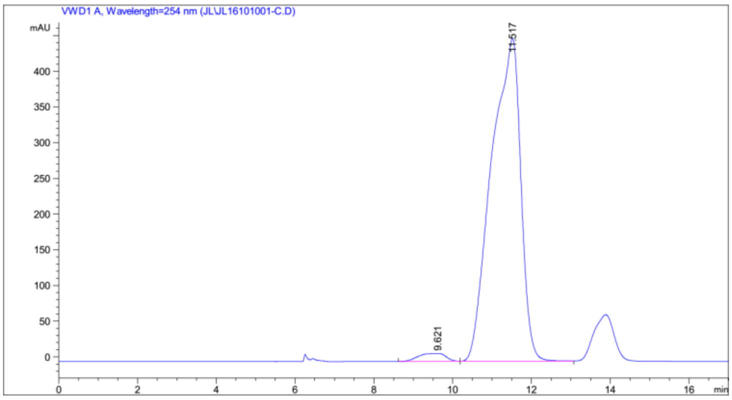

峰 #	保留时间 [min]	类型	峰宽 [min]	峰面积 [mAU*s]	峰高 [mAU]	峰面积 %
1	9.621	BV	0.6358	528.56335	11.15546	2.2168
2	11.517	VB	0.6909	2.33148e4	451.78198	97.7832

HPLC chromatogram of **4m′**

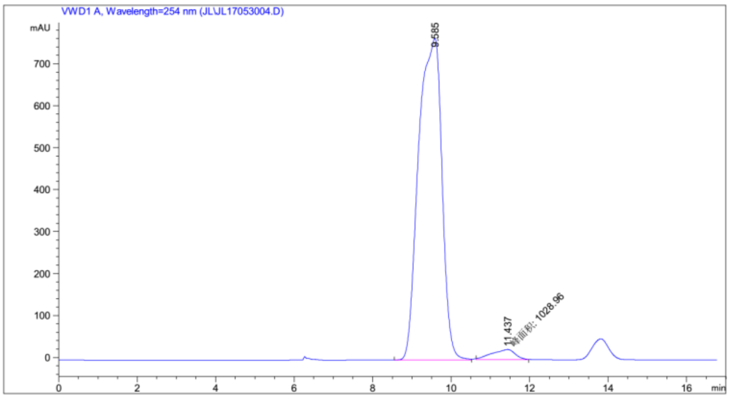

峰 #	保留时间 [min]	类型	峰宽 [min]	峰面积 [mAU*s]	峰高 [mAU]	峰面积 %
1	9.585	BV	0.5521	3.15561e4	765.85803	96.8422
2	11.437	MM	0.6992	1028.95996	24.52723	3.1578

NMR spectra and HPLC chromatogram of **4n** and **4n′**

^{1}H and ^{13}C NMR spectra of **4n** and **4n′**

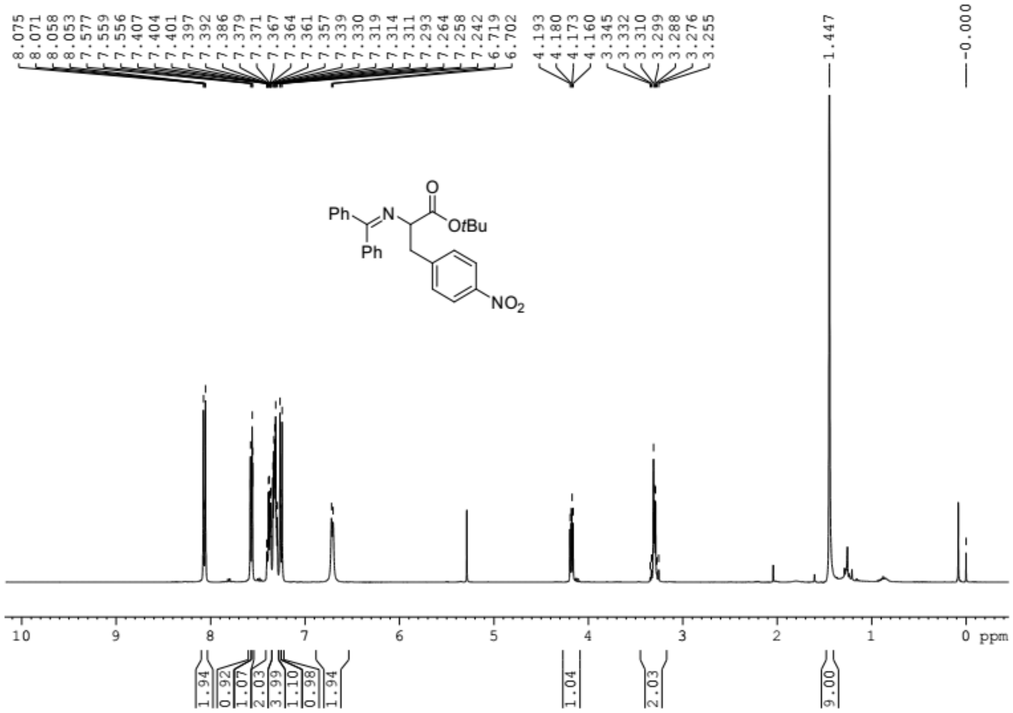

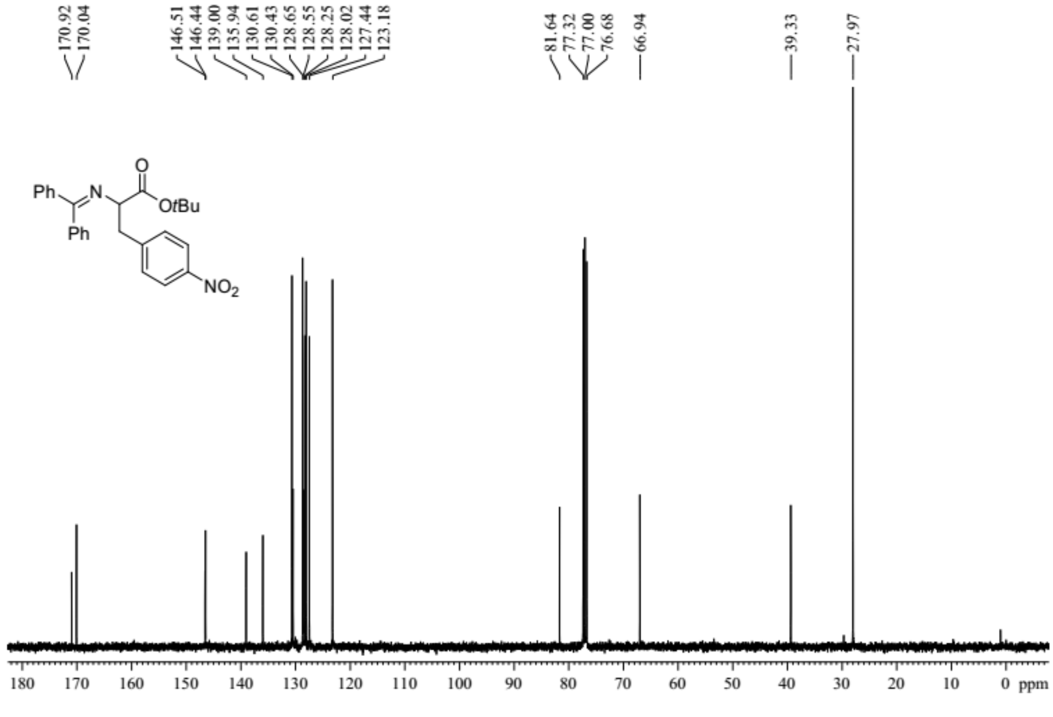

HPLC chromatogram of *rac*-**4n (4n')**

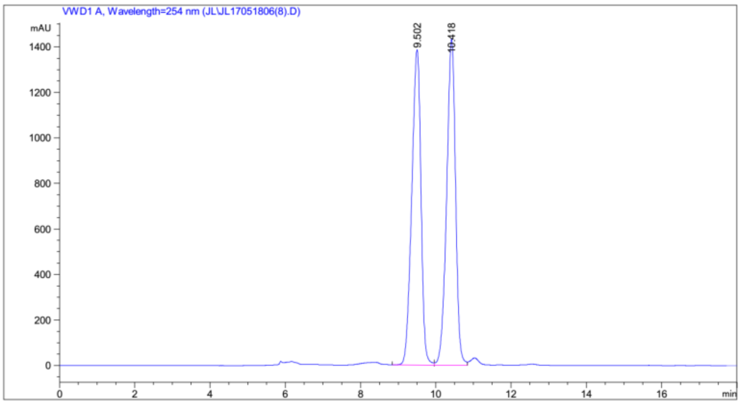

峰 #	保留时间 [min]	类型	峰宽 [min]	峰面积 [mAU*s]	峰高 [mAU]	峰面积 %
1	9.502	BV	0.2621	2.35259e4	1386.28088	49.9362
2	10.418	VV	0.2532	2.35860e4	1433.11316	50.0638

HPLC chromatogram of **4n**

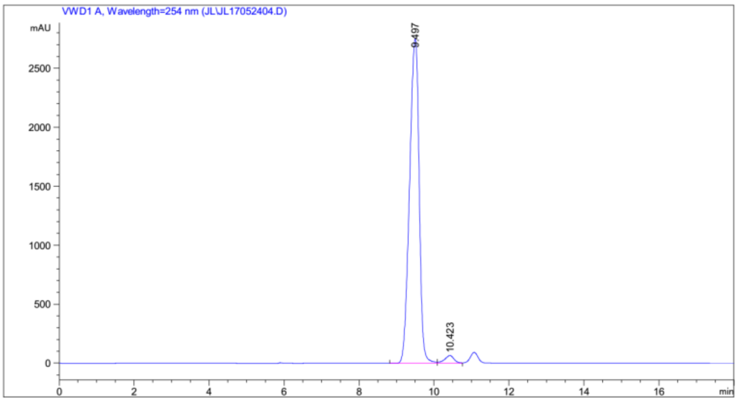

峰 #	保留时间 [min]	类型	峰宽 [min]	峰面积 [mAU*s]	峰高 [mAU]	峰面积 %
1	9.497	BV	0.2765	4.87463e4	2752.07056	97.6909
2	10.423	VV	0.2626	1152.19336	65.32389	2.3091

HPLC chromatogram of **4n'**

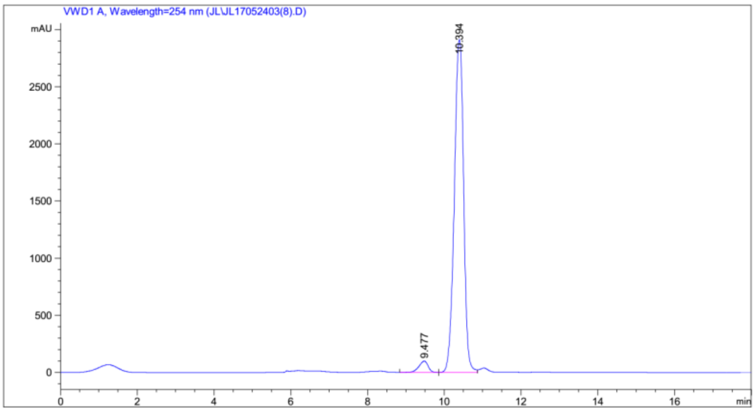

峰 #	保留时间 [min]	类型	峰宽 [min]	峰面积 [mAU*s]	峰高 [mAU]	峰面积 %
1	9.477	BB	0.2557	1626.01794	98.26515	3.2221
2	10.394	BV	0.2597	4.88388e4	2912.57715	96.7779

NMR spectra and HPLC chromatogram of **4o** and **4o′**

^{1}H and ^{13}C NMR spectra of **4o** and **4o′**

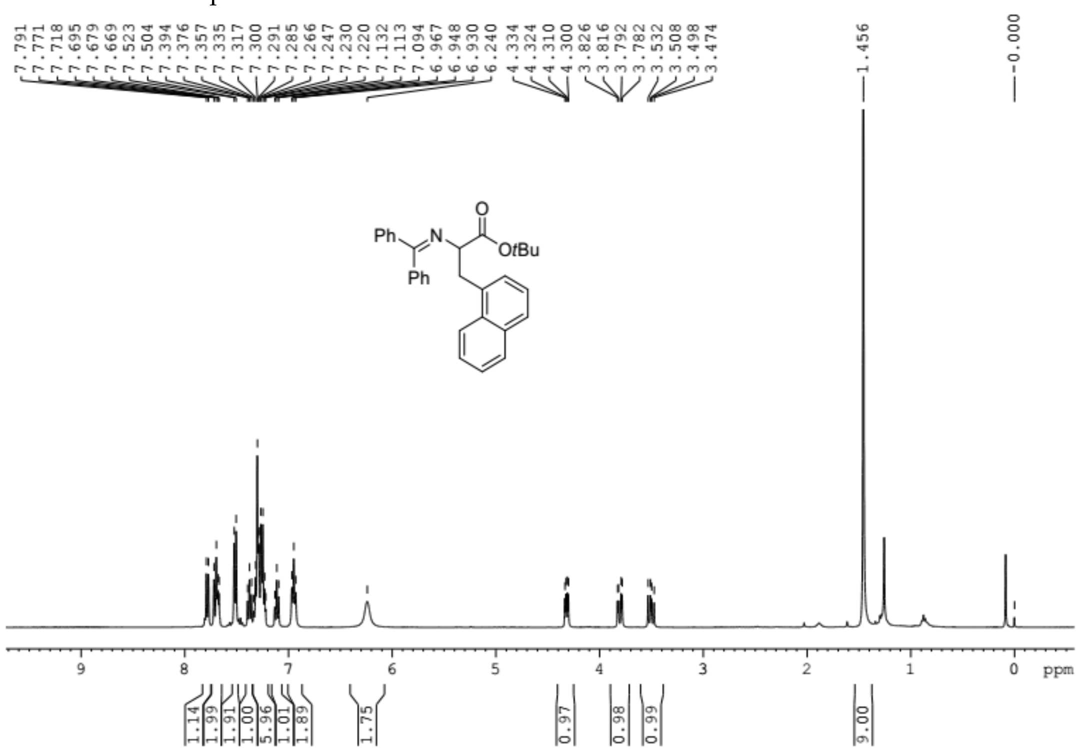

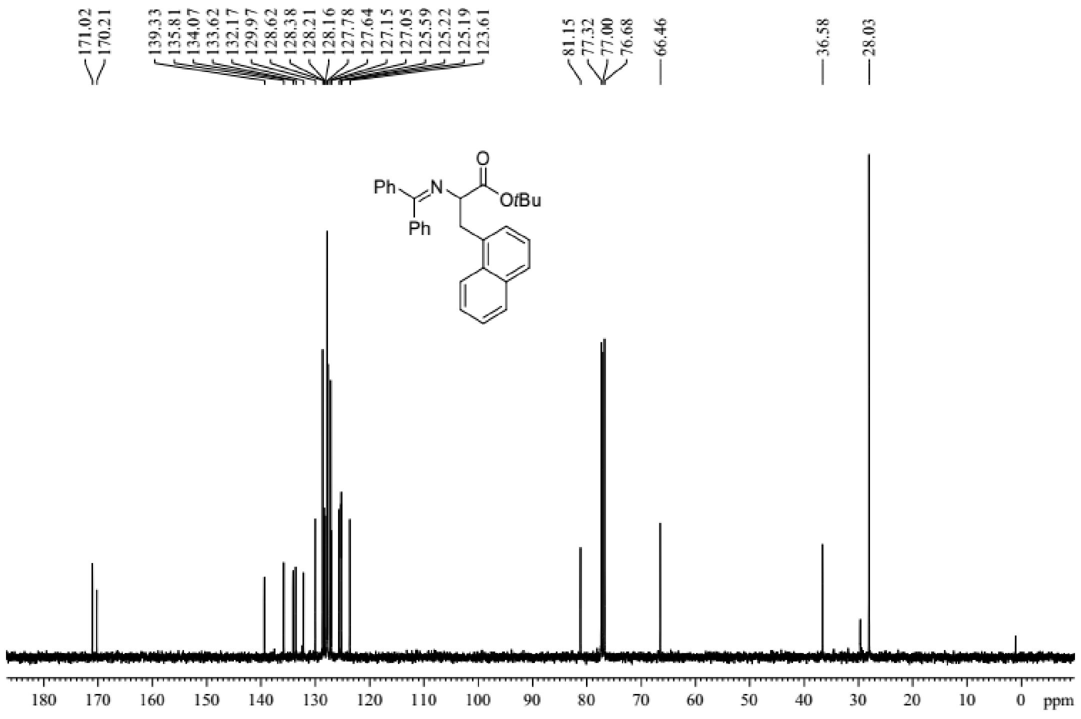

HPLC chromatogram of *rac*-**4o (4o')**

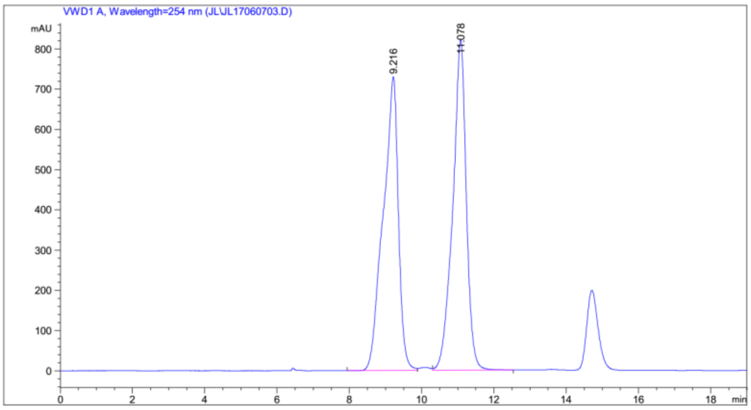

峰 #	保留时间 [min]	类型	峰宽 [min]	峰面积 [mAU*s]	峰高 [mAU]	峰面积 %
1	9.216	BV	0.3994	2.12554e4	729.92163	49.8020
2	11.078	VB	0.3740	2.14245e4	821.79059	50.1980

HPLC chromatogram of **4o**

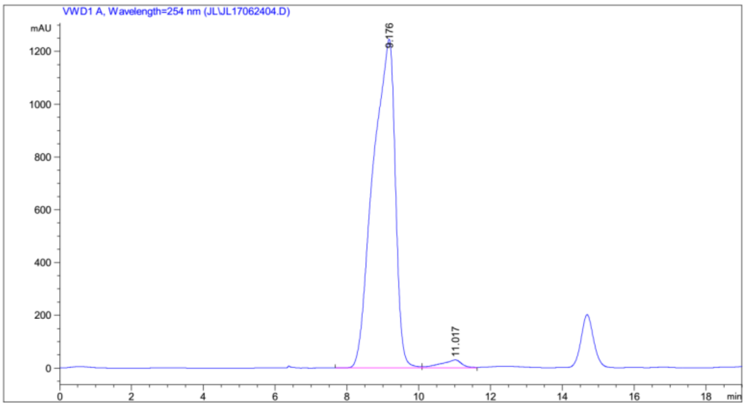

峰 #	保留时间 [min]	类型	峰宽 [min]	峰面积 [mAU*s]	峰高 [mAU]	峰面积 %
1	9.176	BV	0.5754	5.33437e4	1245.51160	97.7920
2	11.017	VV	0.5371	1204.40552	30.16798	2.2080

HPLC chromatogram of **4o;**

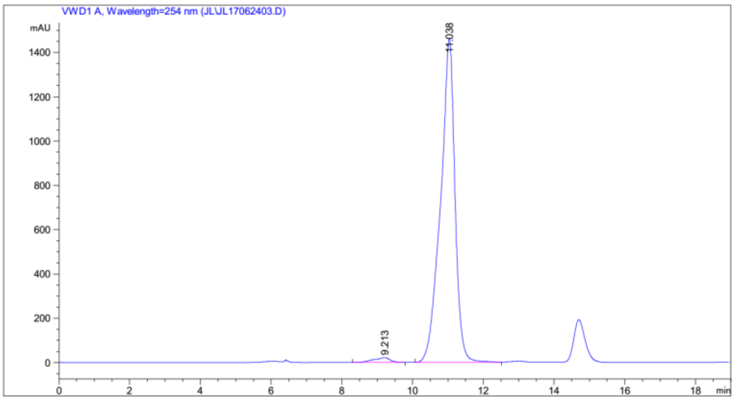

峰 #	保留时间 [min]	类型	峰宽 [min]	峰面积 [mAU*s]	峰高 [mAU]	峰面积 %
1	9.213	BV	0.4411	708.93121	21.60378	1.6816
2	11.038	VV	0.3980	4.14482e4	1460.83630	98.3184

NMR spectra of **5**

^{1}H and ^{13}C NMR spectra of **5**

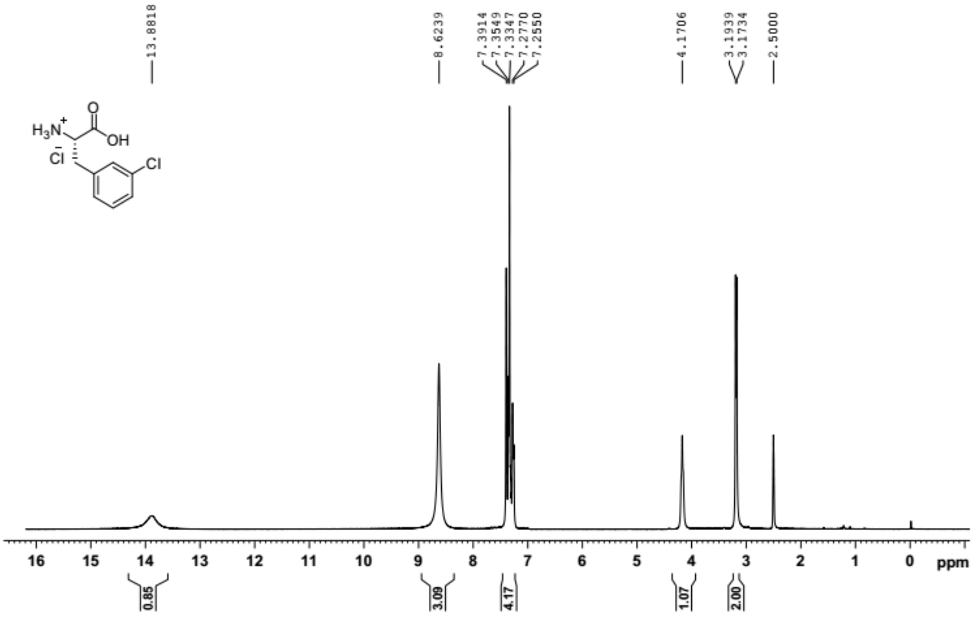

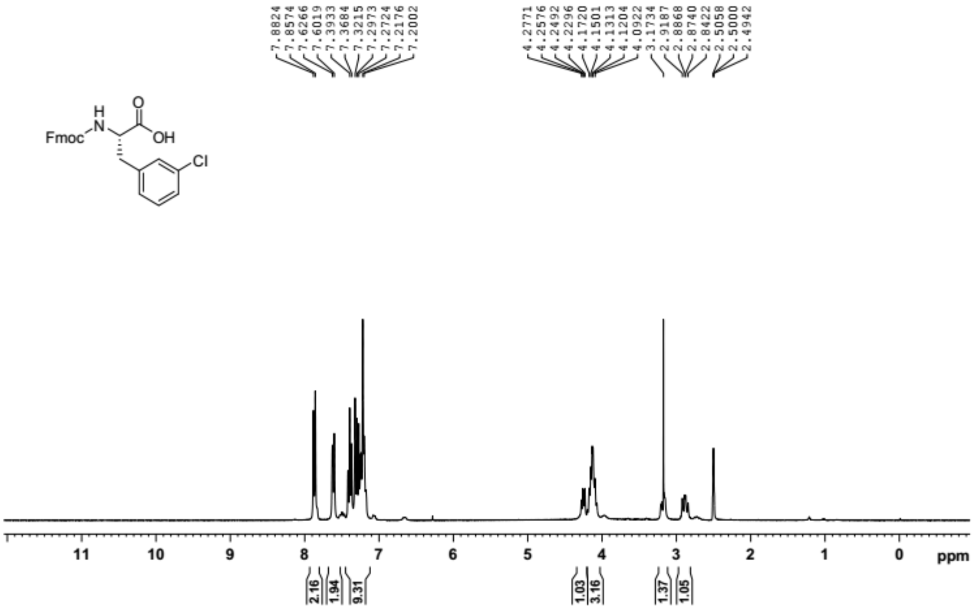

NMR spectra of **6**

^{1}H and ^{13}C NMR spectra of **6**

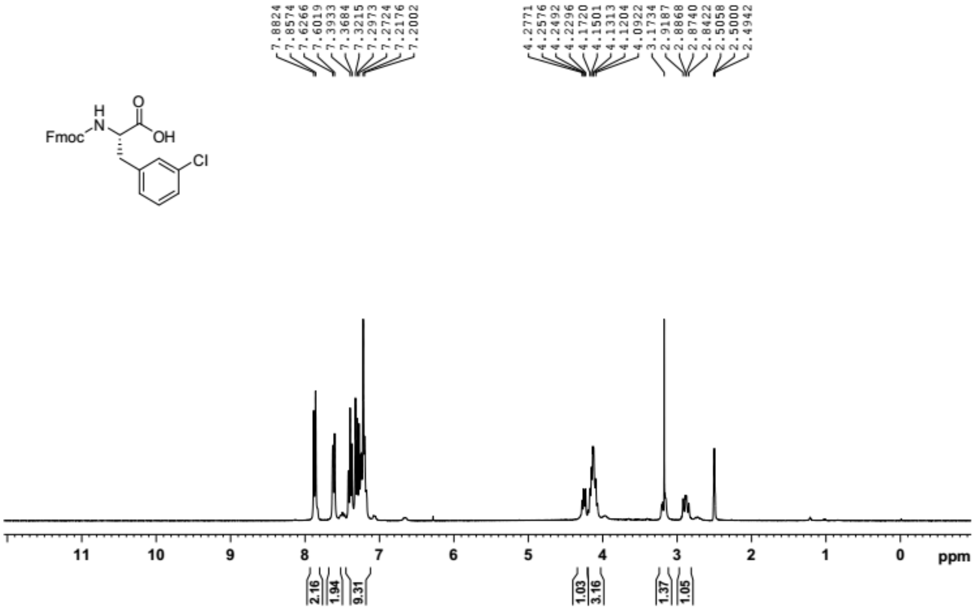

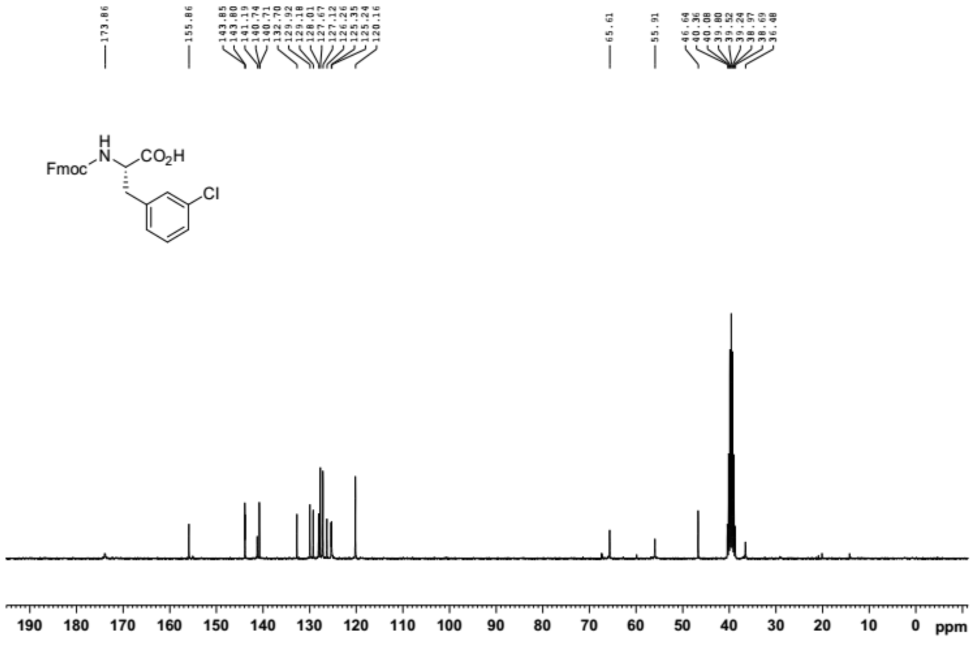

NMR spectra and HPLC chromatogram of **7**

^{1}H and ^{13}C NMR spectra of **7**

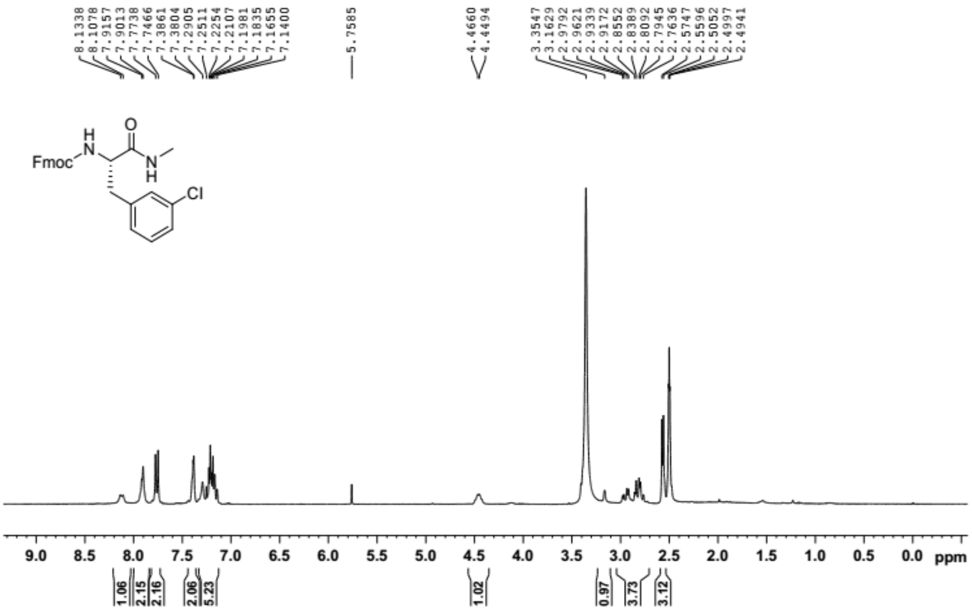

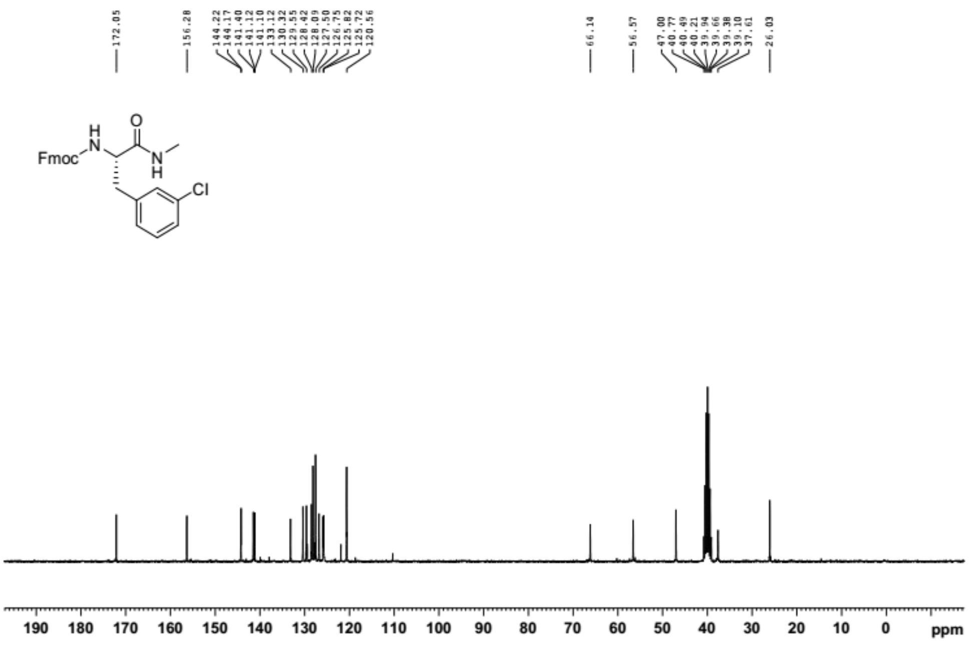

HPLC chromatogram of *rac*-**7**

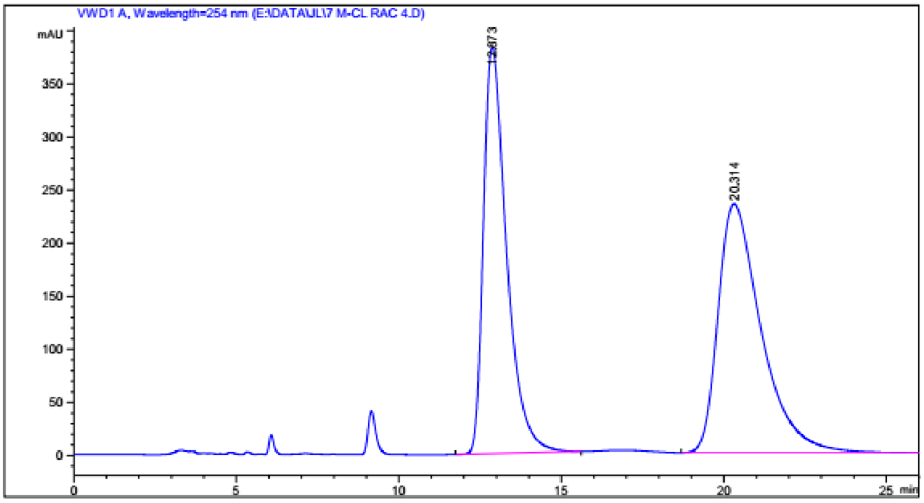

峰 #	保留时间 [min]	类型	峰宽 [min]	峰面积 [mAU*s]	峰高 [mAU]	峰面积 %
1	12.873	BB	0.7446	1.89137e4	382.44073	46.5372
2	20.314	BB	1.3872	2.17284e4	234.47906	53.4628

HPLC chromatogram of **7**

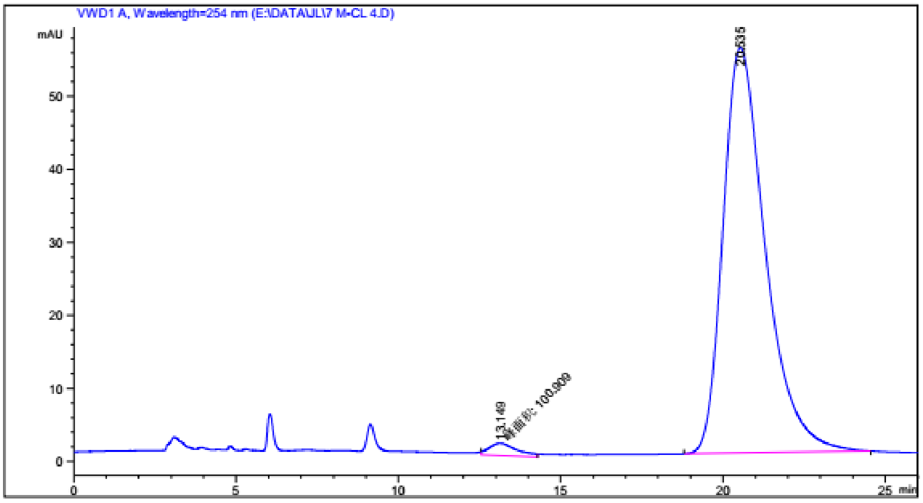

峰 #	保留时间 [min]	类型	峰宽 [min]	峰面积 [mAU*s]	峰高 [mAU]	峰面积 %
1	13.149	MM	1.0039	100.90891	1.67532	1.9623
2	20.535	BB	1.3419	5041.57861	55.53823	98.0377

NMR spectra of **8**

^{1}H and ^{13}C NMR spectra of **8**

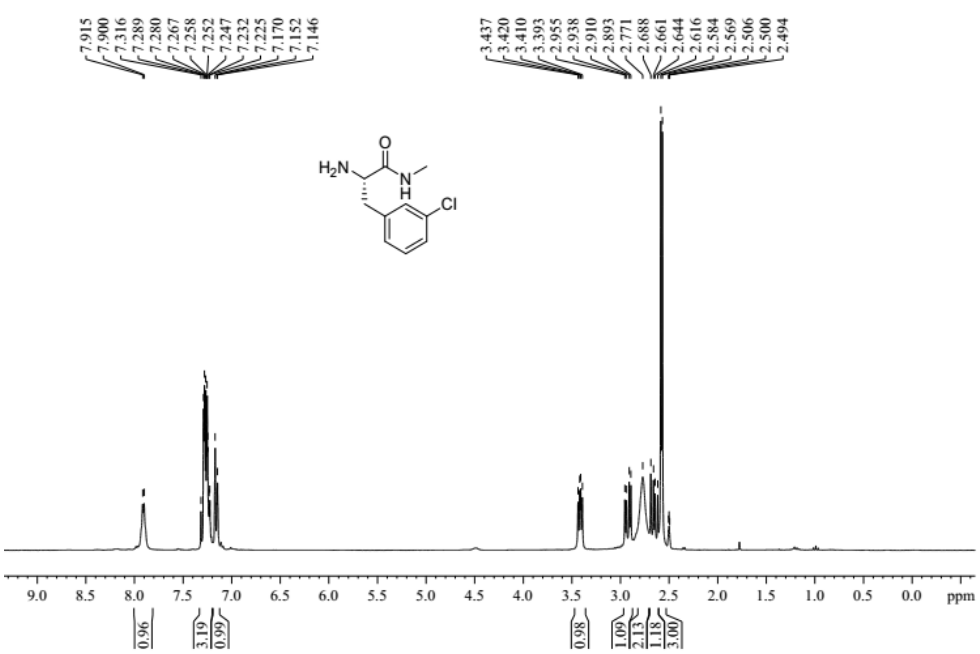

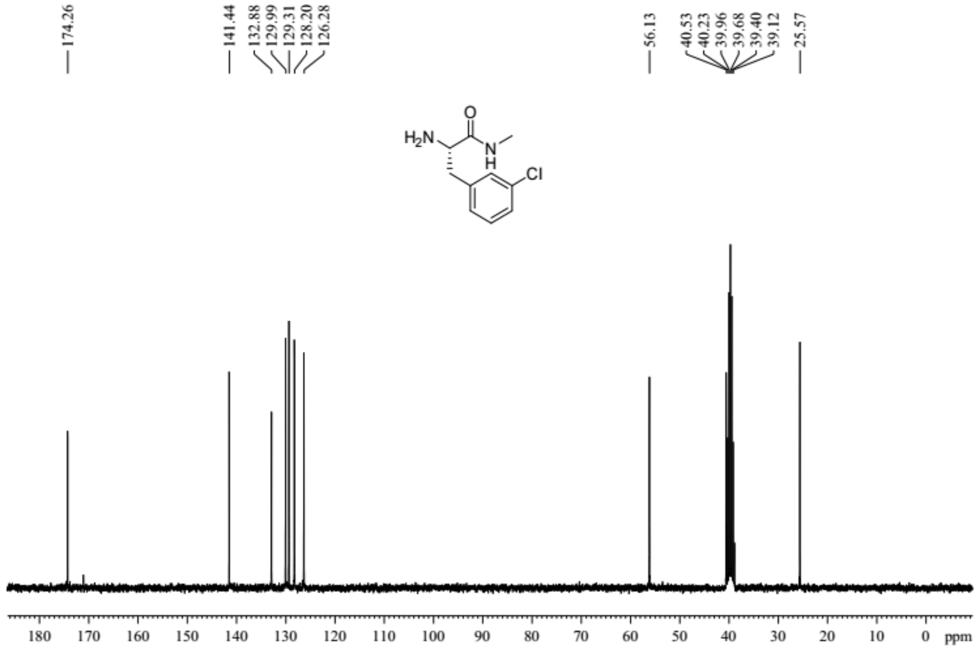

NMR spectra of **10**

^{1}H and ^{13}C NMR spectra of **10**

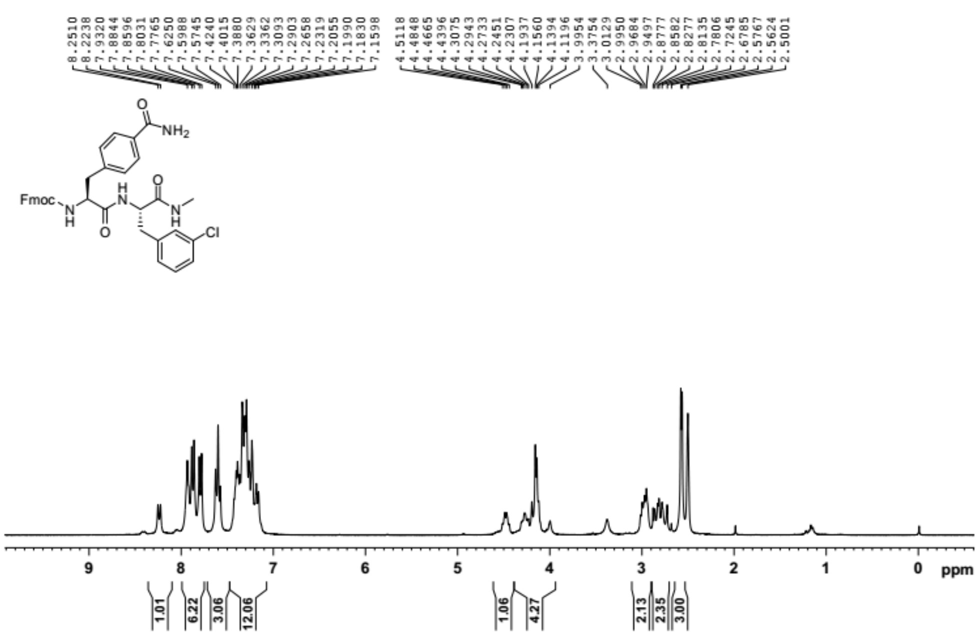

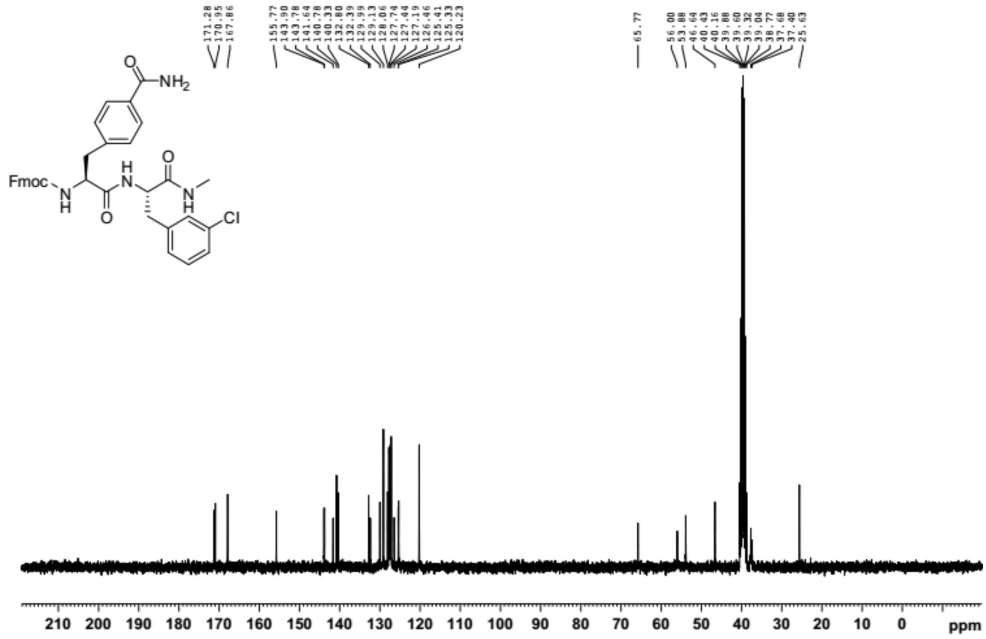

NMR spectra of **11**

^{1}H and ^{13}C NMR spectra of **11**

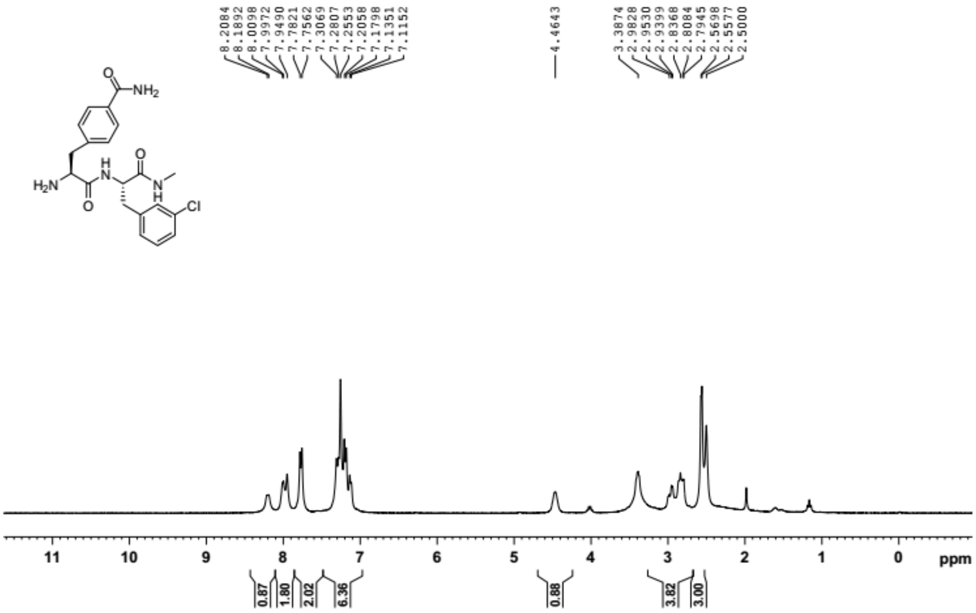

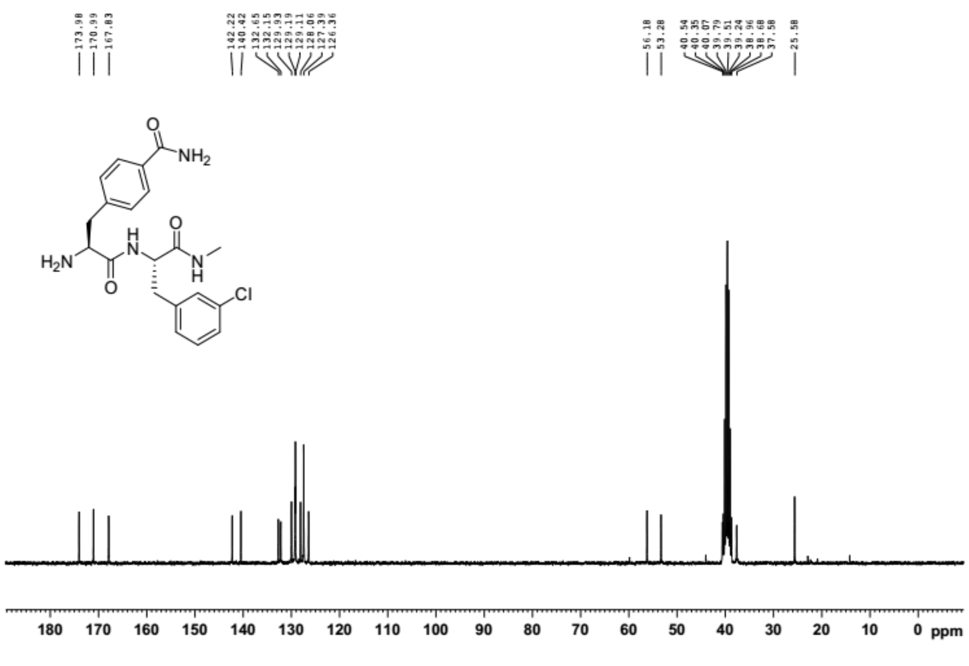

NMR spectra of **13**

^{1}H and ^{13}C NMR spectra of **13**

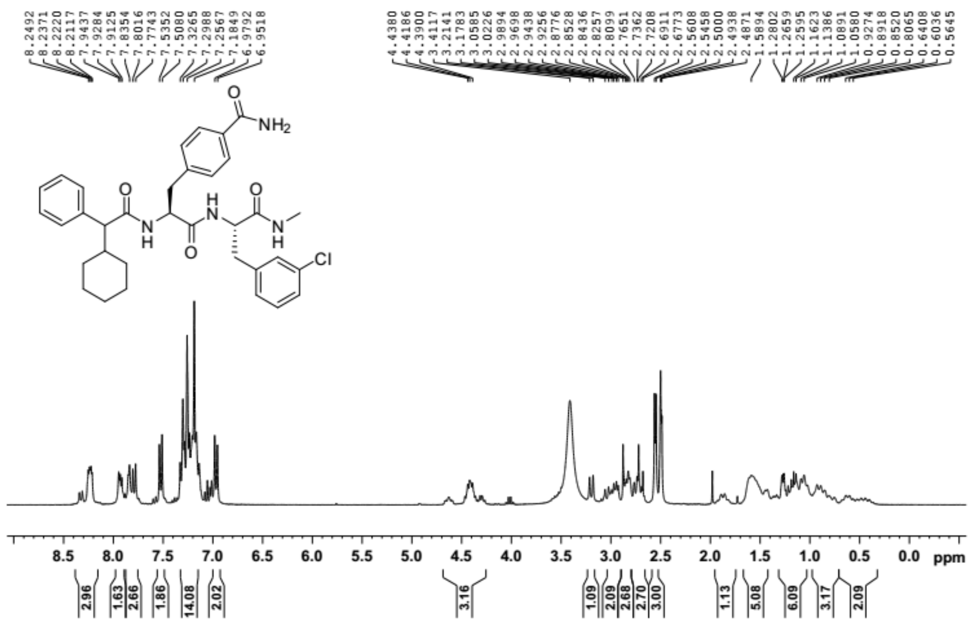

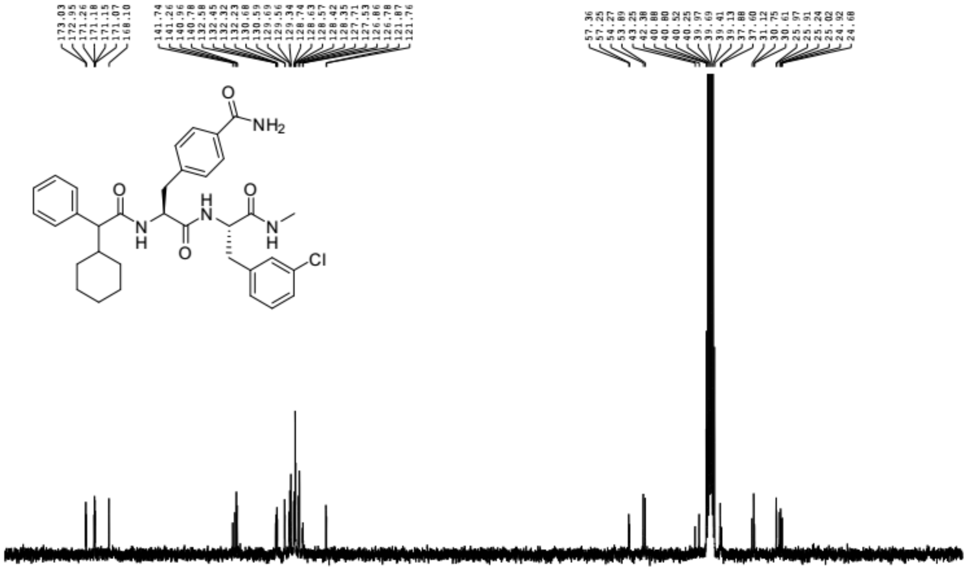

Supplement: Supplementary file 1 [file molecules-23-01421-s001.pdf]
